# Syntaxonomical Remarks on the Garrigues from Apulia (S Italy) and Neighboring Territories

**DOI:** 10.3390/plants13131800

**Published:** 2024-06-29

**Authors:** Valeria Tomaselli, Saverio Sciandrello, Pietro Minissale, Luigi Forte, Emanuele Costanzo, Gianpietro Giusso del Galdo, Francesca Carruggio, Gaetano Pazienza, Salvatore Brullo

**Affiliations:** 1 Department of Biosciences, Biotechnologies and Environment, University of Bari, Via E. Orabona 4, 70126 Bari, Italy; luigi.forte@uniba.it (L.F.); gaetano.pazienza@uniba.it (G.P.); 2 Institute of Atmospheric Pollution Research, National Research Council of Italy (CNR-IIA), Via Amendola 173, 70126 Bari, Italy; costanzo.emanuele@gmail.com; 3 Department of Biological Geological and Environmental Sciences, University of Catania, Via A. Longo 19, 95125 Catania, Italy; s.sciandrello@unict.it (S.S.); p.minissale@unict.it (P.M.); g.giusso@unict.it (G.G.d.G.); salvo.brullo@gmail.com (S.B.); 4 Botanic Garden Museum, University of Bari, Via E. Orabona 4, 70126 Bari, Italy; francesca.carruggio@uniba.it

**Keywords:** shrub vegetation, Puglia, S Italy, *Cisto cretici-Micromerietea julianae*, *Cisto eriocephali-Ericion multiflorae*, *Cytiso spinescentis-Saturejion montanae*, syntaxonomy, phytosociology

## Abstract

In this study, the garrigues occurring in Apulia and neighboring territories (southern Italy) were surveyed in order to clarify their syntaxonomical arrangement. Many contributions previously focused on this vegetation type, often adopting different and sometimes contrasting treatments from both the nomenclature and syntaxonomical aspects. Our investigations are supported by the multivariate analysis of a dataset containing 292 phytosociological relevés, whose resulting cluster dendrogram highlights the hierarchical relationships between the examined plant communities. Overall, twenty-one associations with several sub-associations were recognized. Some of them are already known in the literature, whereas others are described here for the first time. As concerns the syntaxonomical framework, this vegetation is attributable to the class *Cisto cretici-Micromerietea julianae*, which in this territory is represented by the order *Cisto eriocephali-Ericetalia manipuliflorae* and by two alliances: *Cisto eriocephali-Ericion multiflorae*, grouping the more thermophilous associations usually distributed along coastlines and at low altitudes; and *Cytiso spinescentis-Saturejion montanae*, including the mesophilic associations occurring in mountain and sub-mountain belts.

## 1. Introduction

Xerophilous shrub vegetation, often thorny and cushion-like, known as garrigue, phrygana, or batha, is widespread across Mediterranean regions. These plant communities occur from coastal to mountain belts on various substrates, including limestone, marl, clay, vulcanite, granite, schist, gneiss, and sand. As highlighted by [1], as well as by [2,3], in the Central Mediterranean and especially in the Eastern Mediterranean area, there are no clear differences in floristic composition between the basophilous and acidophilous plant communities since most shrub species are indifferent to the occurrence or fewer carbonates in circulating solutions and, accordingly, to the soil pH. Consequently, [1] refers these Central and Eastern Mediterranean shrub communities into *Cisto cretici-Micromerietea julianae* Oberdorfer 1954. Specifically, they consider this class as a geographical vicariant in the Central and Eastern Mediterranean area of the *Rosmarinetea officinalis* Rivas Martinez, T.E. Diaz, F. Prieto, Loidi & Penas 2002 and *Cisto ladaniferi-Lavanduletea stoechadis* Br.-Bl. in Br.-Bl., Molinier & Wagner 1940, both classes having mainly a western Mediterranean distribution. The most recent syntaxonomical arrangements regarding the plant communities previously included in these classes strongly contrast each other without providing any clarification that could be used for reliable classification. Among the authors who dealt with this vegetation type, the authors of [1,4,5,6] can be mentioned as those who treated the higher rank syntaxa previously described in a rather heterogeneous way, especially in their hierarchical position within the various phytosociological classes. Based on our investigations on the plant communities occurring in the Central Mediterranean territories, this shrub vegetation shows a significant floristic affinity regardless of the substrate nature, in contrast to what happens in the western Mediterranean. This ecological pattern had already been highlighted by several authors [1,2,3,7], who deemed it appropriate to consider valid the class *Cisto-Micromerietea julianae* and to treat it as a geographical vicariant of the *Rosmarinetea officinalis* and *Cisto-Lavanduletea*. *Cisto-Micromerietea julianae* can be distinguished into two orders, such as *Cisto-Ericetalia manipuliflorae* Horvatić 1958 for the Central Mediterranean, and *Poterietalia spinosi* Eig 1939, for the Eastern Mediterranean.

With regard to the Apulian region (Southern Italy), these garrigues characterize large parts of the territory, especially in coastal areas but also in hilly, sub-mountain, and mountain belts. This vegetation was investigated by several authors [1,4,8,9,10,11,12,13,14,15,16,17,18,19,20,21], who described several plant communities with different syntaxonomical arrangements. However, there are still many gaps to be filled throughout the whole area; furthermore, some of the above-mentioned contributions have sometimes produced contrasting results, for which clarifications and insights are needed. Thus, a contribution to the *Cisto-Micromerietea julianae* class in Apulia and neighboring areas, on the basis of both published and unpublished phytosociological relevés and supported by multivariate analysis, is provided here, with the aim of (a) increasing the knowledge about this class in the surveyed area, filling existing gaps in numerous plant communities of major phytogeographical interest; (b) clarifying the syntaxonomical position of the identified communities also at higher rank levels; (c) characterizing floristic, ecological, physiognomic–structural, chorological, and nomenclatural aspects of the surveyed syntaxa.

### Study Area

**Figure 1:**
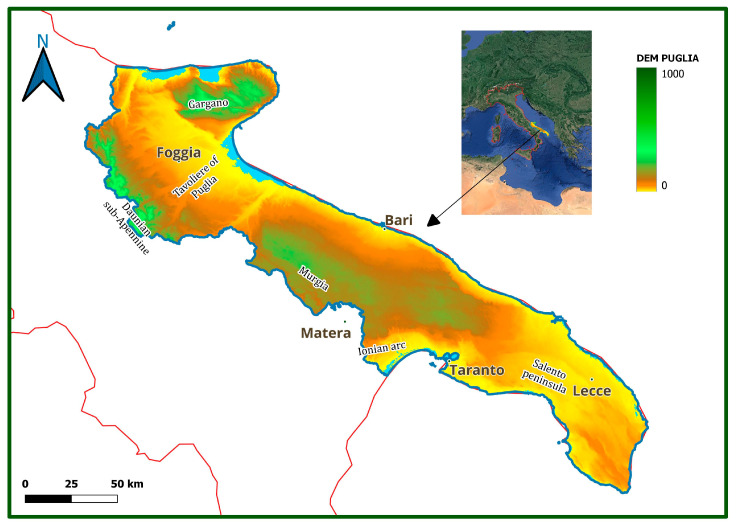
Study area (http://www.sit.puglia.it/portal/portale_cartografie_tecniche_tematiche/Cartografie%20Tematiche/DTM; accessed on 15 April 2024).

Apulia region, localized in the Southeastern part of the Italian peninsula, has a surface area of more than 19,000 km^2^ and about 1000 km of coastline. From North to South, it is possible to identify the following geographical districts: Gargano, Daunian sub-Apennine, Tavoliere of Puglia, Murgia, Ionian arc, and Salento Peninsula (Figure 1).

A peculiarity of this region is the low average altitude, with 53% of its territory being represented by lowlands and 45% by hilly areas [22], with the only exceptions being the Gargano and Sub-Apennine areas, with the top regional altitudes (1055 and 1152 m a.s.l., respectively). Geologically, most of the study area is characterized by Cretaceous limestones and Miocene calcarenites, as well as by alluvial deposits (Pliocene–Pleistocene). Coastal areas include sand dune systems (the most extensive located in the northern part of the region and then along the Ionian arc), alternating with high and low rocky coasts of a limestone or calcarenite nature [22]. The region has a Mediterranean climate, with hot and dry summers and mild winter seasons. The average temperatures are about 15–16 °C, with higher values in the Ionian–Salento area and lower ones in the Daunian sub-Apennine and Gargano. The average annual rainfall values are extremely variable: the rainiest areas are Gargano and Daunian sub-Apennine, where the average yearly values are higher than 800 mm. In the rest of the regional area, the average yearly values range between 500 and 700 mm, with minimum values (less than 500 mm) recorded in the Tavoliere of Puglia and the Ioanian Salento [23]. The bioclimate is mostly Mediterranean Pluviseasonal oceanic, with thermotypes between upper thermomediterranean and lower supramediterranean, and ombrotypes between lower dry and lower subhumid; Salento and coastal areas are weak euoceanic while the inner areas and the coasts of the gulf of Manfredonia are weak semicontinental. Some areas extend up to the Temperate Oceanic, almost exclusively variant submediterranean, with thermotypes between upper thermotemperate and lower supratemperate, and ombrotypes between lower subhumid and lower humid; the vast majority of these areas are weak semicontinental while only very limited parts are weak euoceanic [24]. The largest part of the region (more than 80%) is used for agriculture, while natural and semi-natural areas cover about 14%. As in other arid and semi-arid Mediterranean areas, a large part of the Apulia Region is affected by land degradation and desertification due to the interaction of a set of natural and anthropogenic factors having different temporal and spatial variability [25].

## 2. Results and Discussion

### 2.1. Statistical Analysis

According to the basic statistics implemented on the matrix, the average number of species per relevé is 20.6 (min 6 and max 42) with a median of 19 and a standard deviation of 7.9. The area of relevés is extremely variable, ranging from 8 to 400 sqm (average 61.04), with a median of 50 and a standard deviation of 46.14.

**Figure 2:**
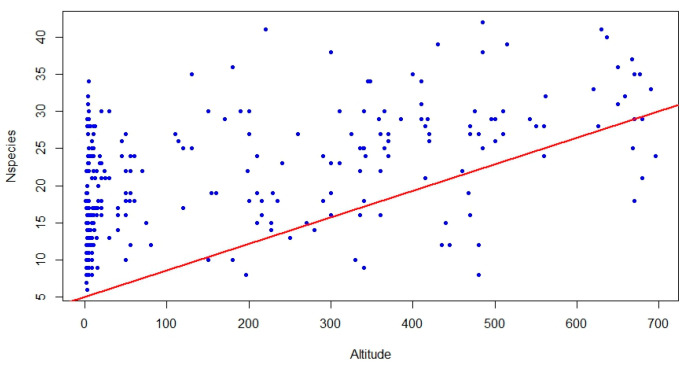
Graphic resulting from the correlation analysis between relevé area and number of species per relevé (species richness).

The correlation analysis between the area of relevés and species richness provided an R^2^ of 0.005 and a *p*-value of 0.45, indicating no significance and, therefore, no correlation, while the correlation between species richness and altitude provided an R^2^ of 0.9 and a *p*-value of 0.00153, revealing a significant positive correlation (Figure 2).

**Figure 3:**
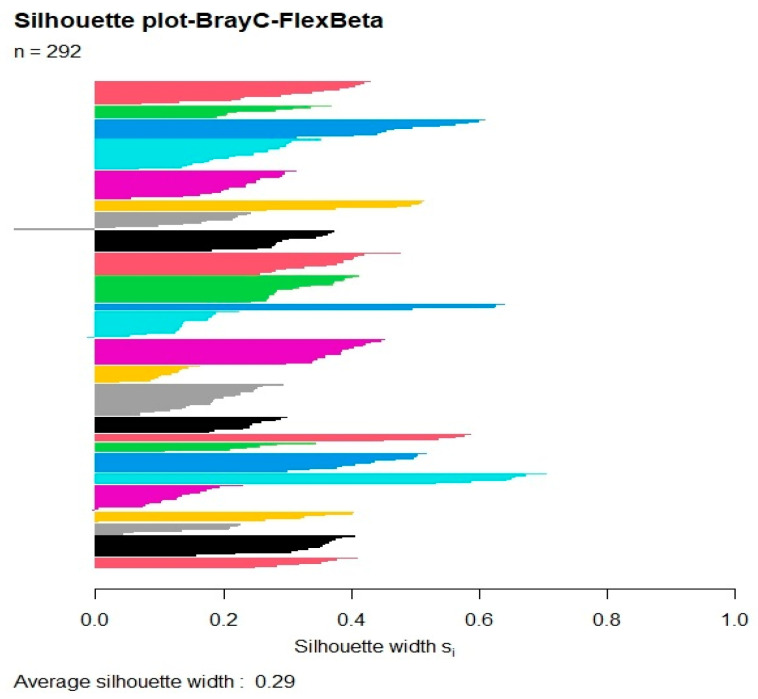
Graphic displaying the Silhouette at 25-group partition.

The average silhouette plot was calculated for all possible partitions (from 2 to 292); the Silhouette value has a sudden increase starting from 23 groups (0.29) with a maximum then at 68 groups (0.33). Considering 68 as a too high number of communities for vegetation type and area investigated, we considered the 25-group partition, which has a Silhouette of 0.29, as good (Figure 3) because this number corresponds well to the communities observed in the area.

**Figure 4:**
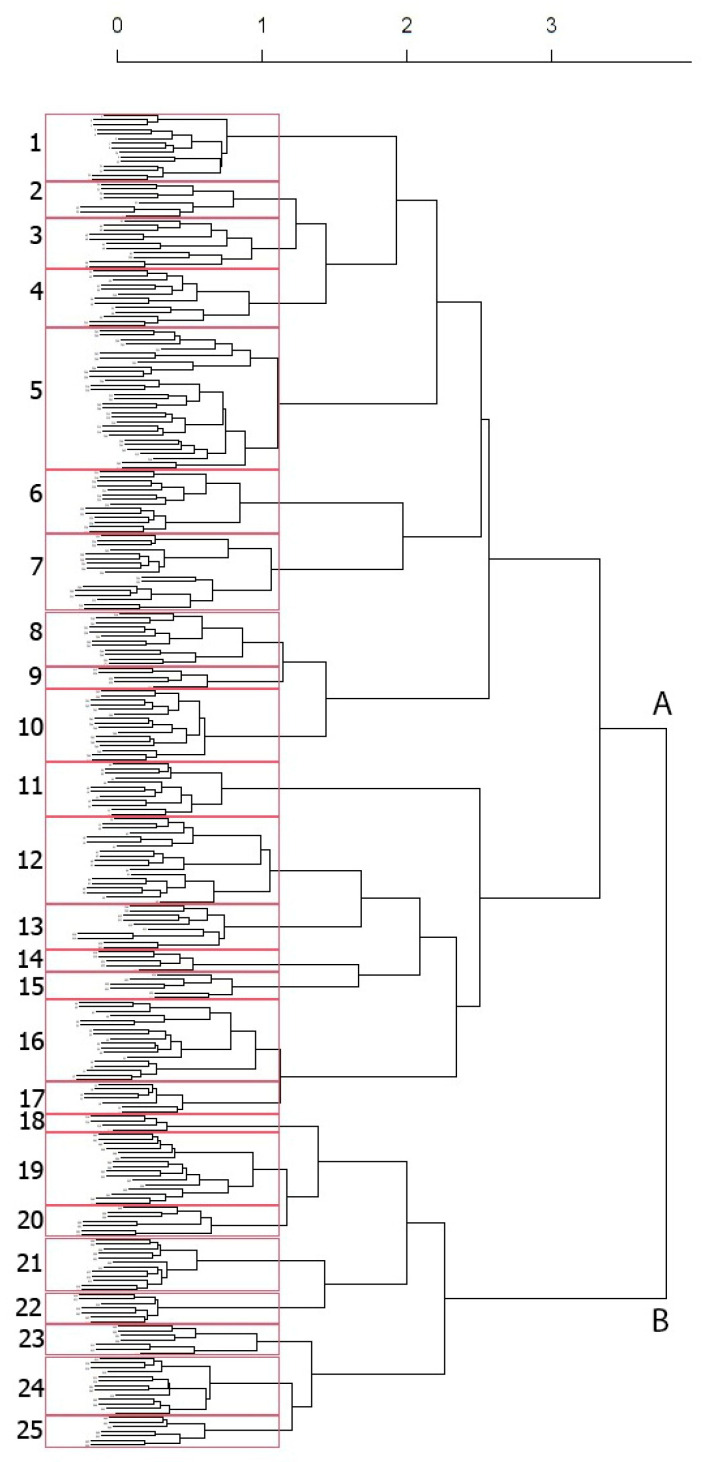
Dendrogram resulting from the cluster analysis (Bray Curtis, Flexible Beta) of the dataset, pruned at 25 groups distributed in two alliances: A—*Cisto eriocephali-Ericion multiflorae*; B—*Cytiso spinescentis-Saturejion montanae*; 1—*Loto-Thymetum capitati* s.l.; 2—*Loto-Thymetum capitati* subass. *plantaginetosum albicantis*; 3—*Dauco gummiferis-Thymelaeetum hirsutae*; 4—*Cisto monspeliensis-Sarcopoterietum spinosi*; 5—*Helianthemo jonii-Thymetum capitati*; 6—*Thymbro capitatae-Anthyllidetum japygicae*; 7—*Saturejo cuneifoliae-Ericetum manipuliflorae*; 8—*Vicio giacominianae-Helianthemetum jonii*; 9—*Cisto eriocephali-Phlomidetum fruticosae*; 10—*Plantago holostei-Thymbretum capitatae*; 11—*Phagnalo-Fumanetum thymifoliae*; 12—*Helianthemo jonii-Fumanetum thymifoliae*; 13—*Sileno otitis*; 14—*Ruto chalepensis-Salvietum trilobae*; 15—*Chamaecytiso spinescentis-Cistetum eriocephali*; 16—*Erico multiflorae-Halimietum halimifolii*; 17—*Cistetum salvifolio-clusii*; 18—*Fumano ericifoliae-Centaureetum subtilis*; 19—*Centaureo subtilis-Thymetum capitati*; 20—*Chamaecityso spinescentis-Genistetum michelii*; 21—*Fumano ericifoliae-Centaureetum subtilis* subass. *typicum*; 22—*Fumano ericifoliae-Centaureetum subtilis* subass. *genistetosum michelii*; 23—*Rhamno saxatili-Saturejetum montanae* subass. *typicum*; 24—*Rhamno saxatili-Saturejetum montanae* subass. *fumanetosum procumbentis*; 25—*Rhamno saxatili-Saturejetum montanae* subass. *asyneumetosum limonifolium*.

In Figure 4, the cluster dendrogram is pruned at 25 groups. At the highest separation, the cluster divides into a main group (A), including most of the relevés (440), and a second group (B) of 27 relevés; the two main clusters correspond to the highest ranks at the alliance level, that is *Cisto eriocephali-Ericion multiflorae* Biondi 2000 and *Cytiso spinescentis-Saturejion montanae* Pirone and Tammaro 1997. The 25 groups represent the main vegetation units at the association and sub-association levels.

**Figure 5:**
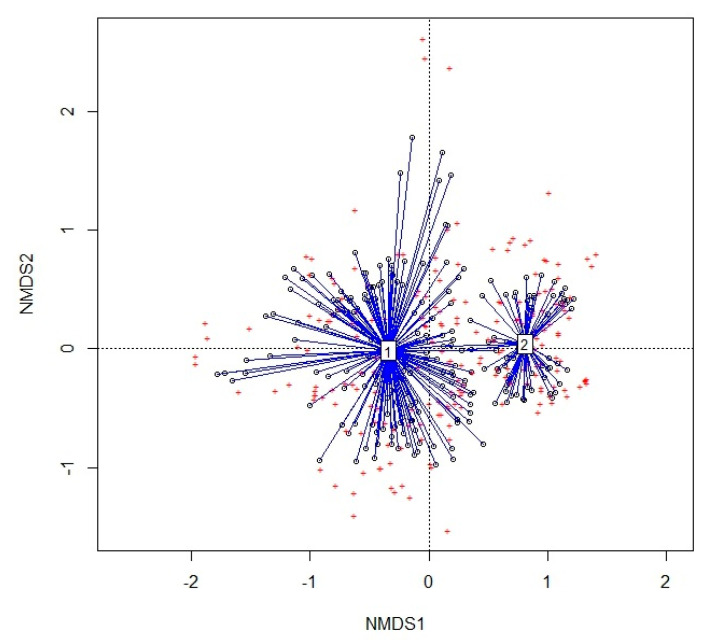
NMDS analysis of the dataset; relevés in Group 1 belong to *Cisto-Ericion multiflorae*, and relevés in Group 2 to *Cytiso spinescentis-Saturejion montanae*.

Figure 5 shows the outcome of the NMDS analysis. The two main groups, 1 and 2, correspond to the two alliances *Cisto-Ericion multiflorae* and *Cytiso spinescentis-Saturejion montanae,* respectively, and are quite good, with a stress of 0.22.

**Figure 6:**
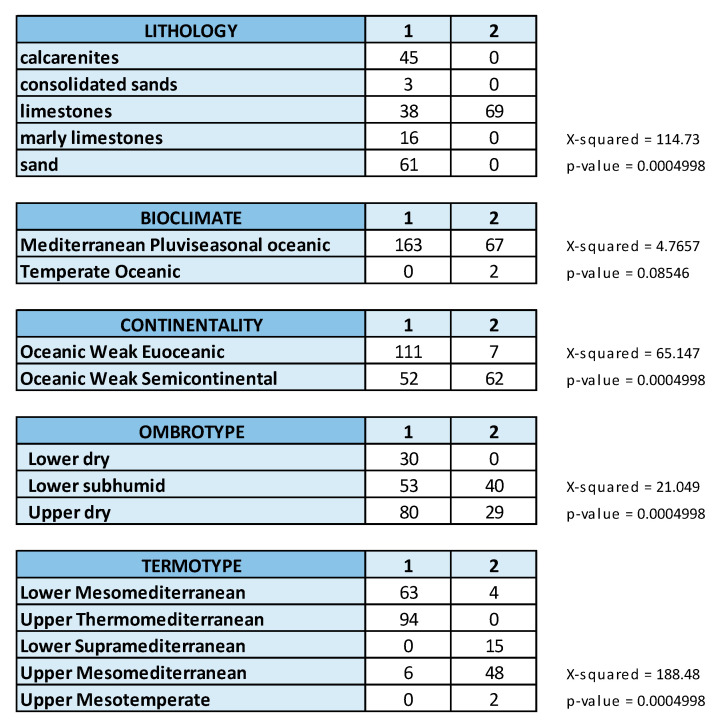
Crosstabs for the evaluation of frequency distribution of the relevés by groups (alliances: 1 = *Cisto-Ericion multiflorae*; 2 = *Cytiso spinescentis-Saturejion montanae*) and by ecological variables.

Figure 6 shows the crosstabs implemented with the multivariate frequency distribution of the relevés by groups (alliances) and by ecological qualitative variables, as well as the results of Pearson’s Chi-squared test performed to determine the association between groups and considered variables. The association turned out to be very significant (*p* < 0.001) except for bioclimate (macrobioclimate).

**Figure 7:**
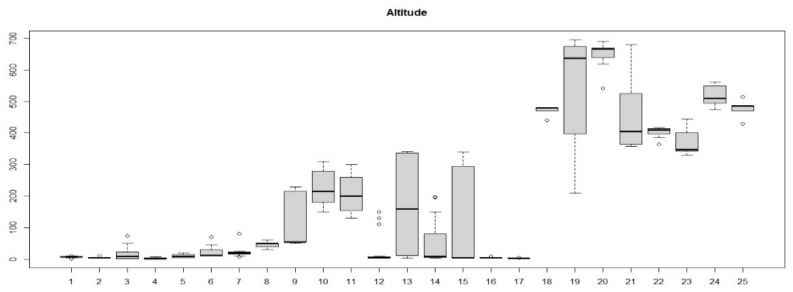
Boxplot showing the distribution of the relevés in the different groups (communities) identified based on the altitude.

**Figure 8:**
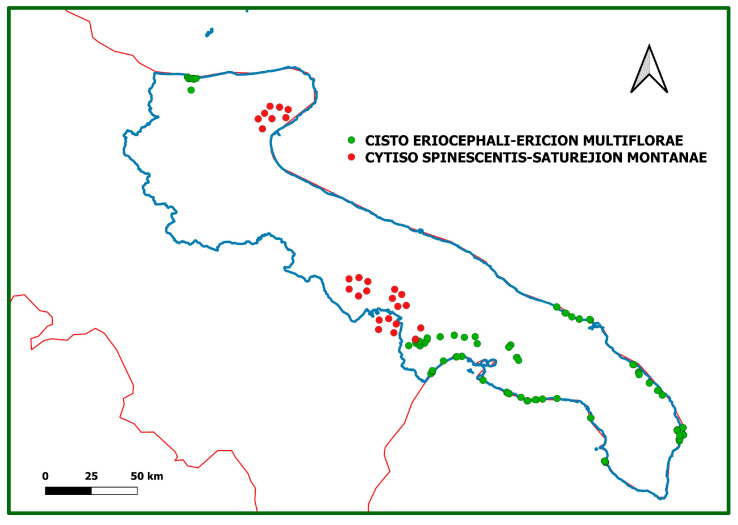
Geographic distribution of the sites where relevés in *Cisto-Ericion multiflorae* (green dots) and of *Cytiso spinescentis-Saturejion montanae* (red dots) were performed.

The boxplot in Figure 7 shows the distribution of the relevés in different communities identified based on the altitude: the different altitudinal distribution between the relevés in communities 1–16 (*Cisto-Ericion multiflorae*) and those of communities 17–25 (*Cytiso spinescentis-Saturejion montanae*) appears very clear. Figure 8 shows the geographic distribution of the two alliances.

### 2.2. Description of the Vegetation

Based on the results of the multivariate analysis described in the previous section, twenty-one associations and nine subassociations were recognized and arranged in two distinct alliances of *Cisto-Micromerietea julianae* class, *Cisto eriocephali-Ericion multiflorae,* and *Cytiso spinescentis-Saturejion montanae*. In the following sections, each syntaxon is provided with floristic, structural, ecological, chorological, and nomenclatural descriptions. In order to summarize part of this information, a summary table is provided in Appendix D, with the association names and their distribution, lithology, and bioclimate.

*CISTO-MICROMERIETEA JULIANAE* Oberdorfer 1954, Vegetatio 5–6: 91

Holotypus: *Cisto-Micromerietalia* Oberdorfer 1954, Vegetatio 5–6: 91.

Syn.: *Erico-Cistetea* Trinajstić 1985, Poljoprivreda i šumarstvo 31(1): 51, p.p.

**Figure 9:**
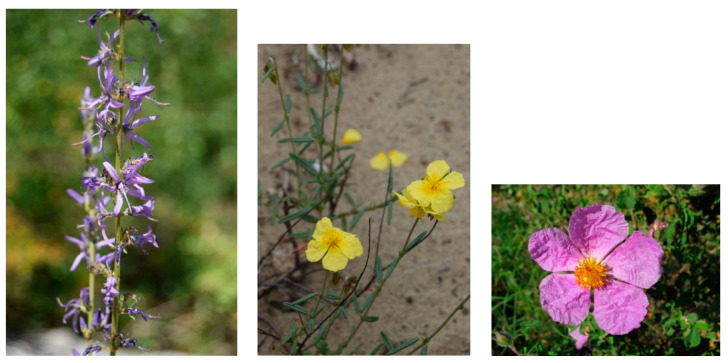
From the left: *Asyneuma limonifolium* subsp. *limonifolium*, *Helianthemum jonium*, *Cistus creticus* subsp. *eriocephalus*, characteristic species of *Cisto-Micromerietea julianae*, *Cisto-Ericetalia manipuliflorae*, and *Cisto eriocephali-Ericion multiflorae*, respectively.

Characteristic species occurring in Apulia: *Asyneuma limonifolium* subsp. *limonifolium* (Figure 9), *Cistus creticus* subsp. *creticus*, *C. monspeliensis*, *C. salviifolius*, *Cytinus hypocistis*, *Cytisus villosus*, *Erica forskalii*, *Euphorbia spinosa*, *Fumana arabica*, *F. ericifolia*, *F. laevis*, *F. scoparia*, *F. thymifolia*, *Globularia alypum*, *Helichrysum italicum* subsp. *italicum*, *Lavandula stoechas*, *Micromeria graeca* subsp. *graeca*, *M. juliana*, *M. nervosa*, *Phagnalon rupestre* subsp. *illyricum*, *P. rupestre* subsp. *rupestre*, *Ononis pusilla*, *Phlomis fruticosa*, *Rosmarinus officinalis*, *Salvia fruticosa*, *Teucrium capitatum* subsp. *capitatum*, *Thymbra capitata*.

Structure and ecology: It groups the chamaephytic and nanofanerophytic thermo-xerophilous and meso-xerophilous plant communities, often rich in pulvinate and thorny species, represented by garrigues, phrygana or batha growing through the Central and Eastern Mediterranean on different substrates (limestone, marl, clay, vulcanite, granite, schist, gneiss, and sand). The vegetation belonging to this class is widespread from the coastal to the mountain belt within the thermo-mesomediterranean and thermo-mesotemperate belts.

Distribution: In the Central Mediterranean territories, the class occurs in the Central–Southern Italian peninsula (including Adriatic, Tyrrhenian, and Ionian sides), Sicily, and Sardinia, while in the Eastern Mediterranean, it is widespread from the Balkan peninsula (including the Aegean area) to Western and Southern Anatolia, Cyprus, Syria, Lebanon, Israel, and Cyrenaica.

*CISTO-ERICETALIA MANIPULIFLORAE* Horvatić 1958, Acta Botanica Croatica 17: 24

Holotypus: *Cisto-Ericion manipuliflorae* Horvatić 1958, Acta Botanica Croatica 17: 23.

Syn.: *Artemisio albae-Saturejietalia montanae* Biondi & Allegrezza in Biondi, Allegrezza, Casavecchia, Galdenzi, Gasparri, Pesaresi, Vagge & Blasi 2014, Plant Biosystems 148(1): 330.

Characteristic species occurring in Apulia: *Helianthemum jonium* (Figure 9), *Hippocrepis comosa*, *H. glauca*, *Leontodon apulus*, *Linum tommasinii*, *Satureja cuneifolia*.

Structure and ecology: As concerns the structural features and ecological requirements of this order, what has already been said for the class applies.

Distribution: The plant communities belonging to this order are distributed in the various countries of the Central Mediterranean area, including the Central and Southern Italian peninsula, Sicily, Sardinia, Malta, North–East Tunisia, the Adriatic coast of Croatia, and Montenegro.

Note: In the Eastern Mediterranean area, such as southern Balkan peninsula, Aegean islands, Western and Southern Anatolia, Cyprus, Syria, Lebanon, Israel, and Cyrenaica, this order is vicaried by the *Poterietalia spinosi* Eig 1939 order (=*Hyperico empetrifolii-Genistetalia acanthocladae* Mucina in Mucina et al., 2016), characterized by a group of species exclusive of the above-mentioned territories or rarely occurring in the Central or western Mediterranean area [1]. Within this order, several alliances were recognized, usually showing a well-circumscribed distribution [1].

*CISTO ERIOCEPHALI-ERICION MULTIFLORAE* Biondi 2000, Coll. Phytosoc. 27: 130

Holotypus: *Rosmarino-Thymetum capitati* Furnari 1965, Boll. Istituto Botanico Univ. Catania, s. 3, 5: 13.

Characteristic species occurring in Apulia: *Cistus creticus* subsp. *eriocephalus* (Figure 9), *Coris monspeliensis*, *Dianthus tarentinus*, *Erica multiflora*, *Lotus hirsutus*, *Micromeria graeca* subsp. *garganica*, *Phagnalon rupestre* subsp. *illyricum*, *Silene vulgaris* subsp. *tenoreana*.

Structure and ecology: This alliance is considered to be a geographical vicariant of *Cisto cretici-Ericion manipuliflorae* Horvatić 1958, including the thermo-xerophilous garrigues distributed mainly in the coastal belt of the Central Mediterranean area. It was described by [4], who included it in the *Rosmarinetalia officinalis* Br.-Bl. ex Molinier 1934, order of the *Rosmarinetea officinalis*, while [6] attribute it more properly to *Cisto-Micromerietalia julianae*. The plant communities referred to as *Cisto eriocephali-Ericion multiflorae* Biondi 2000 are localized within the thermo-mesomediterranean bioclimatic belts because of their thermo-xeric ecological requirements.

Distribution: Adriatic, Ionian, and Tyrrhenian sides of the Italian Peninsula, as well as in Sicily, Malta, Southern Sardinia, and North–East Tunisia.

Notes: This alliance is replaced along the Adriatic coasts of Istria, Dalmatia, and Montenegro by *Cisto cretici-Ericion manipuliflorae* Horvatić 1958, which is floristically differentiated by *Argyrolobium zanonii* (Turra) P.W. Ball, *Centaurea affinis* Friv., *Hieracium stupposum* Rchb., *Lotus herbaceus* (Vill.) Jauzein, *Thymus sibthorpii* Benth., and *Veronica orbiculata* A. Kern. Even if some associations surveyed along the Adriatic coast of the Italian peninsula were previously attributed to this alliance, at the best of current knowledge, *Cisto cretici-Ericion manipuliflorae* seems to be missing in this territory [6].

1. *Loto commutati-Thymetum capitati* Gèhu, Biondi, Gèhu-Frank & Marchiori 1984, Doc. Phytosoc., n.s., 8: 560 (Appendix B, Table A1)

Holotypus: rel. 5. Table 1, [12].

Characteristic and differential species: *Coronilla juncea*, *Helichrysum italicum* subsp. *italicum*, *Lotus creticus*, *Matthiola sinuata*.

**Figure 10:**
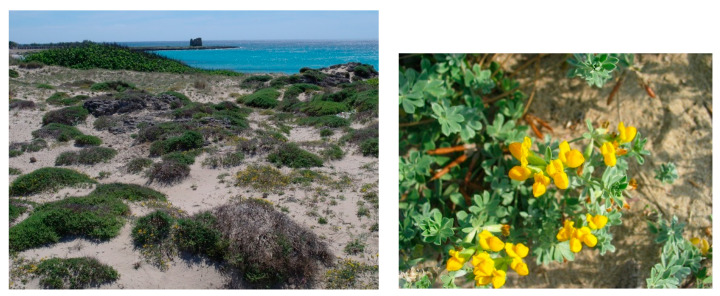
From the left: coastal garrigues of the *Loto commutati-Thymetum capitati*; *Lotus creticus*.

Structure and ecology. The association represents a thermophilous and edaphophilous garrigue growing on sandy soils of coastal dunes and characterized by the dominance of *Thymbra capitata*, usually growing together with *Lotus creticus* (=*L. commutatus*) (Figure 10). It is mainly localized on consolidated substrates of retro-dunal stands, in contact with the associations of the *Euphorbio paraliae-Ammophiletea australis* Géhu & Rivas Martinez in Rivas-Martinez et al., 2011 to the seaside, and inwards with the *Juniperus macrocarpa* maquis. It can be considered an edaphophilous vegetation linked to stabilized sandy soils within the upper thermomediterranean bioclimate with an upper dry to lower dry ombrotype. According to [26], the association, for its structural and ecological features, shows close relations with those of the *Ononidion ramosissimae* Pignatti 1952, while for its floristic set it must be attributed to the *Cisto-Micromerietea* class, especially for the occurrence of some shrubs such as *Thymbra capitata*, *Rosmarinus officinalis*, *Teucrium capitatum*, *Fumana thymifolia*, *Cistus creticus* subsp. *eriocephalus*, and *Helichrysum italicum* subsp. *italicum*. Within this association, some authors [12,13] recognized several subassociations differentiated both ecologically and with regard to the dominance of some species. They are (a) subass. *helichrysetosum italici* characterized by the dominance of *Helichrysum italicum* subsp. *italicum*, growing in the stands closest to the shoreline, which are more affected by marine aerosol; (b) subass. r*osmarinetosum officinalis* growing in the innermost stands with more mature soils, where *Rosmarinus officinalis* is dominant; (c) subass. *cistetosum eriocephali* differentiated by the occurrence of *Cistus creticus* subsp. *eriocephalus*, which can be considered of secondary origin. Moreover, a particular variant of this association, characterized by the occurrence of *Plantago albicans*, has been observed further inland on quite consolidated sands, often forming a mosaic with the *Juniperus macrocarpa* communities. This vegetation is here proposed as *plantaginetum albicantis* Costanzo, Sciandrello & Tomaselli subass. nova hoc loco (Table A1, rel. 16–23; holosyntypus rel. 16, hoc loco).

Distribution. According to data from the literature and personal unpublished relevés, the association occurs in southern–western Apulia, along the Ionian coast, from Taranto to Porto Cesareo, as well as on the Adriatic coast at Torre Guaceto, near Brindisi [12,13,16,26].

2. *Dauco gummiferis-Thymelaeetum hirsutae* Costanzo & Tomaselli, ass. nova hoc loco (Appendix B, Table A2)

Holotypus: rel. 6, Table A2, hoc loco.

Characteristic and differential species: *Daucus carota* subsp. *gummifer*, *Elymus acutus*, *Thymelaea hirsuta*.

Structure and ecology. Along the rocky coast, on calcarenitic substrates, a dwarf garrigue physiognomically dominated by *Thymbra capitata*, usually associated with *Thymelaea hirsuta*, occurs. It is localized within the upper thermomediterranean bioclimate with lower dry to lower subhumid ombrotype. It often covers large surfaces in contact with the sea with the halophilous communities of *Crithmo-Limonietea* Br.-Bl. in Br.-Bl. et al., 1952 nom. mut. This vegetation is characterized as a weak halophile because of the effects of the marine aerosol, as attested by the occurrence of two salt-tolerant species, namely *Daucus carota* subsp. *gummifer* and *Elymus acutus*. For its floristic and ecological features, it is described as a new association, namely *Dauco gummiferis-Thymelaeetum hirsutae*. It usually replaces the inwards halophilous associations of the *Crithmo-Limonietea* and, in particular, the *Agropyro acuti-Helichrysetum italici* Bartolo, Brullo, and Signorello. For the occurrence of *Thymelaea hirsuta* and *Thymbra capitata,* this association is quite similar to the *Thymelaeo hirsutae-Thymetum capitati*, described by [27], from southern Sardinia, but the latter association is localized exclusively on limestone and is characterized by *Satureja thymbra* and other differential species, such as *Phagnalon rupestre* subsp. *annoticum* and *Teucrium polium* subsp. *capitatum*, while *Daucus carota* subsp. *gummifer* and *Elymus acutus* are missing. Another association showing some floristic and ecological relations with the *Dauco gummiferis-Thymelaeetum hirsutae* is the *Thymelaeo hirsutae-Helichrysetum siculi* Bartolo, Bartolo et al., 1992, described from Sicily, where it is linked to limestone and floristically differentiated by *Helichrysum siculum* and *Teucrium polium* subsp. *aureum* [28]).

Distribution. The association has been observed in coastal areas of the Adriatic side, between Brindisi and Otranto, and in the Ionian side near Taranto.

3. *Cisto monspeliensis-Sarcopoterietum spinosi* Brullo, Minissale & Spampinato 1997, Fitosociologia, 32: 43. (Appendix B, Table A3)

Holotypus: rel. 3. Table published by [8].

Characteristic and differential species: *Sarcopoterium spinosum*.

Structure and ecology. In the Salento Peninsula, a garrigue dominated by *Sarcopoterium spinosum* occurs along the coastal area, within the upper thermomediterranean bioclimatic belt with upper dry ombrotype, where it is localized on calcarenitic substrates or sometimes on consolidated dunes. In this vegetation, *Thymbra capitata* and *Cistus monspeliensis* are frequent, and they usually grow together with other species of the *Cisto-Micromerietea* class. From the phytosociological point of view, [8] referred this plant community to the *Poterium spinosum* and *Coridothymus capitatus* (=*Thymbra capitata*) ass., syntaxon described by [29], from Rhodos Island and included in the *Oleo-Ceratonion*. This attribution must be rejected since the Apulian vegetation is floristically and ecologically well differentiated from the association proposed by [29]. Based on this consideration, [1] attributed the vegetation at issue to a new association, namely *Cisto monspeliensis-Sarcopoterietum spinosi*. The association is in contact, in the rocky stands near the sea, with the *Limonietum japygici* Curti & Lorenzoni 1968, belonging to the *Crithmo-Limonietea*, while, inwards, it is in contact with the *Myrto communis-Pistacietum lentisci* (R. Molinier 1954) Rivas-Martinez 1975. Other garrigues characterized by *Sarcopoterium spinosum* and *Thymbra capitata* have also been described in other territories of the Central Mediterranean area; among them can be mentioned the following association: the *Genisto corsicae-Sarcopoterietum spinosi* Biondi & Mossa 1992, occurring in southern Sardinia, growing on limestone and differentiated by *Genista corsica* and *Helichrysum italicum* subsp. *microphyllum* [27]; the *Chamaeropo-Sarcopoterietum spinosi* Barbagallo, Brullo & Fagotto 1979 localized in southern Sicily and differentiated by *Chamaerops humilis* [30,31]; the *Helichryso italici-Sarcopoterietum spinosae* Gèhu & Costa in Gèhu et al., 1984 surveyed on the dry gravelly riverbeds of the Ionian coast in Southern Italy and differentiated by *Helichrysum italicum* subsp. *italicum* [12,13,32].

Distribution. The association shows a very limited range, being circumscribed to two localities in the Salento Peninsula [8,33].

4. *Thymbro capitatae-Anthyllidetum japygicae* Costanzo, Tomaselli, Giusso del Galdo & Brullo ass. nova hoc loco (Appendix B, Table A4)

Holotypus: rel. 10, Table A4, hoc loco.

Characteristic and differential species: *Anthyllis hermanniae* subsp. *japygica*.

**Figure 11:**
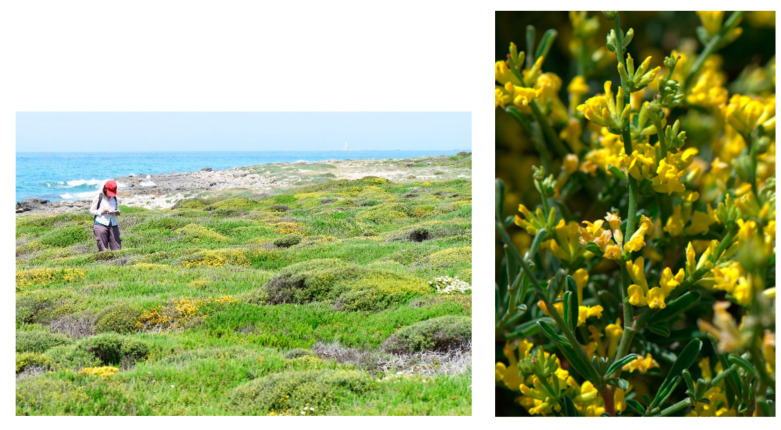
From the left: coastal garrigues of the *Thymbro capitatae-Anthyllidetum japygicae*; *Anthyllis hermanniae* subsp. *japygica*.

Structure and ecology. A very rare and geographically circumscribed garrigue occurs along the Ionian coast of Salento, within the upper thermomediterranean bioclimate with an upper dry ombrotype. It is physiognomically characterized by *Anthyllis hermanniae* subsp. *japygica* (Figure 11), an endemic taxon having a very limited distribution in this area, where it represents a geographic vicariant within the *Anthyllis hermanniae* L. species complex [34]. In this vegetation, *Rosmarinus officinalis* and *Thymbra capitata* are usually dominant and grow together with other species of *Cisto-Micromerietea*. The substrate is represented by consolidated sands or calcarenites, where this plant community colonizes the stands quite distant from the shoreline, while in the belt closest to the sea, it is replaced by the *Limonietum japygici*. For its floristic and ecological peculiarities, it is here described as a new association, named *Thymbro capitatae-Anthyllidetum japygicae*, and can be considered as a geographical vicariant of the *Coridothymo-Anthyllidetum brutiae* Brullo et al., 1997 corr. Brullo hoc loco (syn. *Coridothymo-Anthyllidetum hermanniae* Brullo et al., 1997, Fitosociologia 32: 43, nom. inept. art. 43) described from the Ionian coast of Calabria, where it occurs in a similar ecological context, but differentiated by *Anthyllis hermanniae* subsp. *brutia*, circumscribed to the Calabria coast [34].

Distribution. The association is distributed along a short stretch of the Ionian coast of Salento, south of Gallipoli (LE); its main core area is represented by “Punta Pizzo”, while other small patches are scattered in other neighboring localities [35].

5. *Saturejo cuneifoliae-Ericetum manipuliflorae* Brullo, Minissale, Signorello, Spampinato 1987, Arch. Bot. Biogeogr. Ital. 62(3–4): 206 (Appendix B, Table A5)

Holotypus: rel. 7, Table 1, [14].

Characteristic and differential species: *Erica forskalii*, *Lotus herbaceus*.

**Figure 12:**
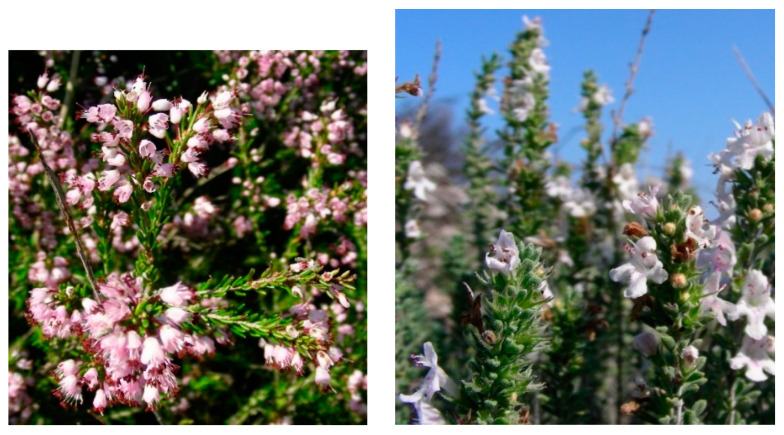
From the left: *Erica forskalii*, *Satureja cuneifolia* (*Saturejo cuneifoliae-Ericetum manipuliflorae*).

Structure and ecology. This association represents a peculiar garrigue physiognomically characterized by *Erica forskalii* (=*Erica manipuliflora* Salisb.) (Figure 12), a species having a wide Eastern Mediterranean distribution in Italy localized in a few stands of the Adriatic coast of the Salento Peninsula, which is the western limit of its range [36]. The bioclimate in this area falls in the upper thermomediterranean with an upper dry to lower subhumid ombrotype. Other shrubs are frequent in this plant community, such as *Rosmarinus officinalis*, *Thymbra capitata*, *Satureja cuneifolia*, *Cistus salviifolius*, and *C. creticus* subsp. *eriocephalus*. This vegetation thrives on flat, calcarenitic surfaces in correspondence with shallow soils with outcropping rocks at low altitudes not exceeding 20 m a.s.l, sometimes in proximity of coastal areas. It is usually in contact with the maquis of the *Arbuto-Quercetum calliprini* Brullo et al., 1986, an association that is considered climacic vegetation in this coastal area [14,37].

Distribution. The association has been observed in numerous sites along the Adriatic side of the Salento Peninsula, in the municipalities of Brindisi and Lecce [14].

6. *Vicio giacominianae-Helianthemetum jonii* Costanzo, Tomaselli, Giusso del Galdo and Brullo ass. nova hoc loco (Appendix B, Table A6)

Holotypus: rel. 8, Table A6, hoc loco.

Characteristic and differential species: *Centaurea tenacissima*, *Helianthemum jonium*, *Vicia giacominiana*.

**Figure 13:**
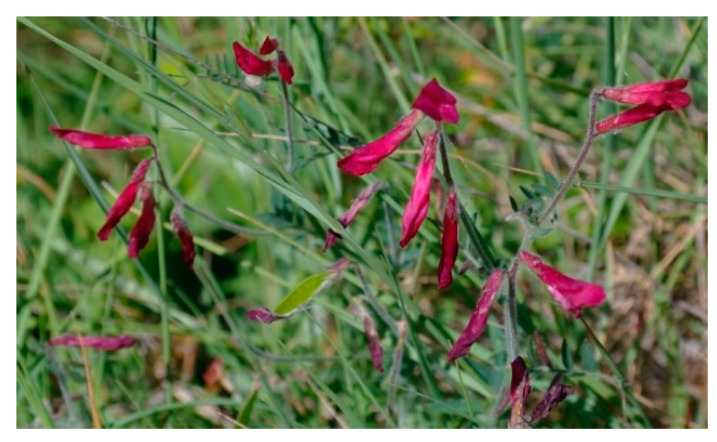
*Vicia giacominiana* (*Vicio giacominianae-Helianthemetum jonii*).

Structure and ecology. In the Apulian territory and especially in the Salento Peninsula, garrigues physiognomically characterized by *Thymbra capitata*, *Satureja cuneifolia*, and *Helianthemum jonium* are very frequent, mainly on carbonatic substrates. In coastal habitats characterized by calcareous outcrops represented by Cretaceous limestones and falling within the upper thermomediterranean lower subhumid belt, a quite specialized plant community was surveyed. It occurs at altitudes between 10 and 80 m. a.s.l., on rocky surfaces with shallow soils deposited mostly in the cracks and sunken areas. This vegetation is floristically well distinct from the other similar communities, apart from the constant occurrence of *Helianthemum jonium*, mainly for the significant frequency of *Vicia giacominiana* (Figure 13), narrowly endemic to a very limited area and considered critically endangered (CR) in Italy [38], and *Centaurea tenacissima*, a rare and endemic species from Puglia and Basilicata. In addition, some endemic species belonging to the East-Mediterranean element are quite frequent, such as *Leontodon apulus*, *Dianthus tarentinus*, *Asyneuma limonifolium* subsp. *limonifolium*, and *Phlomis fruticosa*. This plant community, for its floristic and ecological peculiarities, is here described as *Vicio giacominianae-Helianthemetum jonii* and is included in the *Cisto eriocephali-Ericion multiflorae* alliance. This association belongs to the *Arbuto-Querceto calliprini sigmetum*, which is considered the climacic vegetation of southeastern Salento [14,37].

Distribution. This association is localized near Porto Badisco, a coastal village in the Otranto municipality.

7. *Cisto eriocephali-Phlomidetum fruticosae* Brullo, Scelsi, Spampinato 2001, Veget. Aspromonte: 139 (Appendix B, Table A7)

Holotypus: rel. 3, Table 47A, [39].

Characteristic and differential species: *Phlomis fruticosa*.

**Figure 14:**
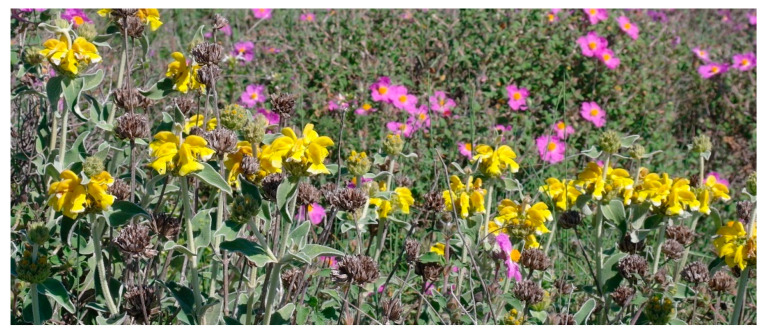
Garrigues of *Cisto eriocephali-Phlomidetum fruticosae*.

Structure and ecology. Garrigues physiognomically dominated by *Phlomis fruticosa* can be observed in the southern part of the Salento Peninsula, in the upper thermomediterranean lower subhumid belt, and at an altitudinal range between 30 and 100 m. a.s.l., where this species usually shows high cover values and grows together with *Cistus creticus* subsp. *eriocephalus* (Figure 14). The primary habitats of this vegetation are probably represented by calcareous screes or stony grounds; only secondarily, it tends to spread in abandoned lands and overgrazed areas [40]. Due to its floristic composition and ecological requirements, this plant community can be referred to as *Cisto eriocephali-Phlomidetum fruticosae* Brullo et al., 2001, described for the Southern Calabria, where it represents secondary vegetation linked to degradation or recolonization processes. In the Apulian territory, this association occurs within the climatophilous area of the *Arbuto-Querceto calliprini sigmetum*. Other associations characterized by *P. fruticosa* were described in the Italian territory [31,41] from the submontane area of the central Apennines (Abruzzo, Italy) as *Sideritido italicae-Phlomidetum fruticosae* Pirone 1995 and southern Sicily, as *Salvio fruticosae-Phlomidetum fruticosae* Barbagallo, Brullo & Fagotto 1979. Later, Ref. [40] stated that the *Sideritido italicae-Phlomidetum fruticosae* is also present in Apulia, but in our opinion, both in terms of floristic composition (none of the diagnostic species indicated for the *Sideritido italicae-Phlomidetum fruticosae* are present in the Apulian communities) and ecology, the communities occurring in Apulia cannot be attributed to this association.

Distribution. In the study area, this association is localized near Otranto on the Salento Peninsula (southern Apulia).

8. *Plantago holostei-Thymbretum capitatae* Tomaselli & Costanzo ass. nova hoc loco (Appendix B, Table A8)

Holotypus: rel. 8, Table A8, hoc loco.

Characteristic and differential species: *Helianthemum leptophyllum*, *Hypericum spruneri*, *Onobrychis alba* subsp. *alba*, *Plantago holosteum*.

**Figure 15:**
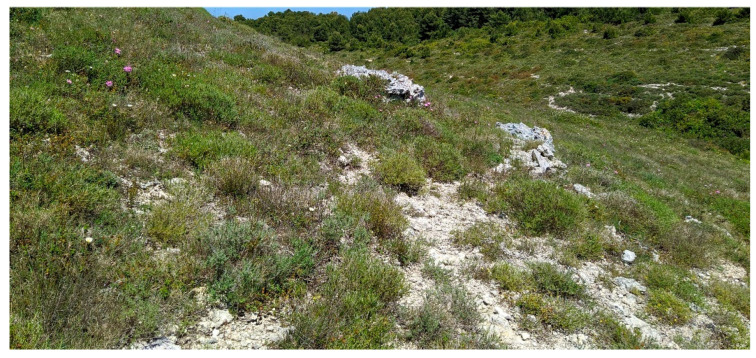
Garrigues of the *Plantago holostei-Thymbretum capitatae*.

Structure and ecology. Garrigues dominated by *Thymbra capitata* and *Satureja cuneifolia*, where *Plantago holosteum* shows high frequency, occurring on marl or marly-limestone substrates (Figure 15) dating back to the Messinian (early Miocene). This vegetation has been observed in the inland of the southern part of Salento, at an elevation between 30 and 70 m a.s.l., in a bioclimate upper thermomediterranean with a lower subhumid ombrotype, which differs floristically very well from the other plant communities belonging to *Cisto eriocephali-Ericion multiflorae*. In fact, apart from *P. holosteum*, this vegetation is characterized by *Helianthemum leptophyllum*, a Western-Mediterranean species known to be present only in Sardinia [42,43]), and here reported for the first time in Apulia, where it occurs exclusively in these stands. Moreover, *Onobrychis alba* subsp. *alba* and *Hypericum spruneri*, both with a southeastern-European distribution, result localized in this community. For the floristic composition and ecological requirements, this vegetation is here proposed as a new association, namely *Plantago holostei-Thymbretum capitatae*. It belongs to the *Hedero helicis-Querceto calliprini sigmetum* [37], which is the climatophilous forest of the inland areas of the Salento Peninsula, of which, after degradation processes, it can represent a secondary stage.

Distribution. It occurs on marly outcrops inland from the Salento Peninsula, near Otranto.

9. *Helianthemo jonii-Thymetum capitati* Biondi & Guerra 2008, Fitosociologia 45 (1) suppl.1: 89 (Appendix B, Table A9)

Syn.: *Helianthemo jonii-Thymetum capitati* Di Pietro & Misano 2010, Acta Bot. Gall. 157(2): 211, nom. illeg. (Art. 29c, 31); *Fumano scopariae-Thymetum capitati* Forte, Carruggio & Mantino 2011, Inform. Bot. Ital., 43 Suppl. 1: 16, nom. nud. (Art. 2); *Phagnalo saxatilis-Saturejetum cuneifoliae* Biondi & Guerra 2008, Fitosociologia 45 (1) suppl. 1: 92 (Art. 29c, 31); *Sedo ochroleuci-Saturejetum cuneifoliae* Di Pietro & Misano 2010, Acta Bot. Gall. 157(2): 212 (Art. 29c, 31).

Holotypus: rel. 4, Table 17, [18].

Characteristic and differential species: *Helianthemum jonium*, *Fumana scoparia*.

**Figure 16:**
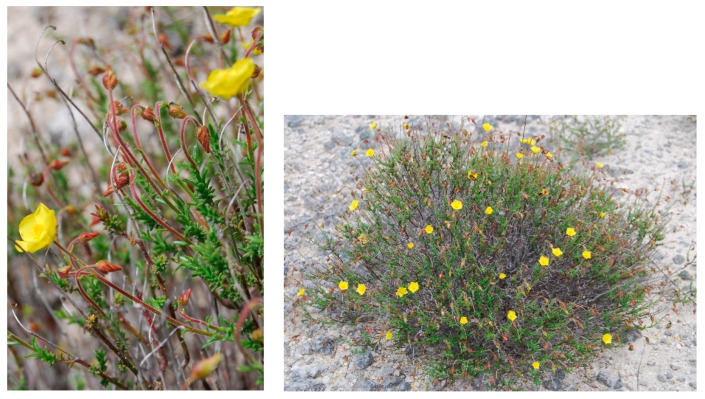
*Fumana scoparia* (*Helianthemo jonii-Thymetum capitati*).

Structure and ecology. The southernmost part of the Murge area, localized in the Ionian arc between Matera and Taranto, is represented by a terraced carbonate tableland incised by Plio-Pleistocenic karst canyons locally named “gravine” [44]. In this place, at altitudes ranging from 100 to 350 m a.s.l., within the bioclimate lower mesomediterranean with an upper dry ombrotype, garrigues dominated by *Thymbra capitata* and rich in numerous thermophilous shrubs, such as *Helianthemum jonium*, *Satureja cuneifolia*, *Hippocrepis glauca*, and various *Fumana* species (*F. thymifolia*, *F. ericifolia*, *F. laevis*, and *F. scoparia*) occurs. Among the latter, *F. scoparia* shows a relevant phytogeographical role because this Mediterranean species is very rare in Italy, where it seems to have its greatest diffusion in this Apulian area [20]. This vegetation was described as *Helianthemo jonii-Thymetum capitati* Biondi & Guerra 2008 [18], and later also by [19], using the same name. Because in the relevés published by these authors [18,19], *F. scoparia* (Figure 16) was not mentioned, a new name for this association (*Fumano scopariae-Thymetum capitati* nom. nud.), without type designation, was proposed by [20]. Considering that *F. scoparia* is widespread in this association and often has high cover values, it is most likely that the previous authors had not distinguished this species from the allied *F. ericifolia*, which often coexists in the same stands. Therefore, the name *Fumano scopariae-Thymetum capitati* must be considered also a “*nomen superfluum*” (Art. 18b, 29c, 31). Within this association can be included the *Phagnalo saxatilis-Saturejetum cuneifoliae* Biondi & Guerra 2008, and the *Sedo ochroleuci-Saturejetum cuneifoliae* Di Pietro & Misano 2010, since for their floristic and ecological characteristics these two plant communities can be considered as impoverished stages of *Helianthemo jonii-Thymetum capitati* and therefore illegitimate names (Art. 29c, 31). As concerns the diagnostic species indicated as characteristics by [18], only *Helianthemum jonium*, amphiadriatic species [45], can be considered as differential species of the association, along with *Fumana scoparia*, since the other taxa are to be included among the characteristics of higher rank. This vegetation represents mostly a secondary stage as a result of degradation processes of *Thymo capitati-Pinetum halepensis* De Marco & Caneva 1984, which is the climacic vegetation on the Taranto Murge calcareous plateau and of the Ionian arc “gravine” [37].

Distribution. The association is widespread in the “gravine” of the Ionian arc (Taranto municipality), as well as in the carbonate tableland in this area.

10. *Phagnalo annotici-Fumanetum thymifoliae* Biondi 2000, Coll. Phytosoc., XXVII: 132 (Appendix B, Table A10)

Holotypus: rel. 2, Table 3, [4].

Characteristic and differential species: *Phagnalon rupestre* subsp. *illyricum*.

Structure and ecology. On the steep gravelly slopes, represented by screes or stone deposits, with soils rich in allochthonous material characterized by medium and fine grain size mixed with red earth, low and open garrigue, physiognomically dominated by *Phagnalon rupestre* subsp. *illyricum* (=*P. rupestre* subsp. *annoticum*) occurs. It was described by [4] and later considered by [18] as *Phagnalo annotici-Fumanetum thymifoliae.* It represents an edaphophilous vegetation linked to carbonate substrates, distributed between 10 and 350 m a.s.l, within the thermophilous *Pinus halepensis* woods. It grows within the lower and upper mesomediterranean bioclimatic belts, with an upper dry to lower subhumid ombrotype. It is a pioneer vegetation that is floristically very poor, whereas *Fumana thymifolia* is the more frequent shrub, often showing high cover values.

Distribution. It was surveyed on the Gargano promontory (FG) and in some localities on the slopes of the Ionian arc “gravine” (TA).

11. *Sileno otitis-Helianthemetum lippii* Tomaselli & Costanzo ass. nova hoc loco (Appendix B, Table A11)

Holotypus: rel. 9, Table A11, hoc loco.

Characteristic and differential species: *Helianthemum lippii*, *Silene otites*.

**Figure 17:**
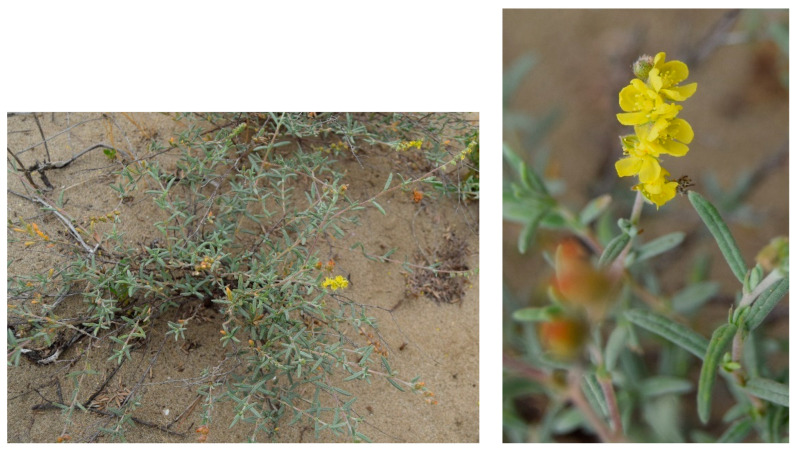
*Helianthemum lippii* (*Sileno otitis-Helianthemetum lippii*).

Structure and ecology. Along the sandy coast of the Ionian arc, West to Taranto, within the upper thermomediterranean bioclimatic belt with a lower dry ombrotype, in the back-dune stands, a vegetation with *Helianthemum lippii* (Figure 17), dominant species having a south-Mediterranean range, occurs. This species is a psammophyte in Italy recorded only from Apulia and Sicily, growing with few other perennial species, such as *Cistus creticus* subsp. *eriocephalus*, *Helianthemum jonium*, *Lotus cytisoides*, and *Silene otites*. This plant community is here proposed as a new association, named *Sileno otitis-Helianthemetum lippii*, which, even if floristically quite poor, is to be referred to as *Cisto cretici*-*Ericion multiflorae*. For its ecology and occurrence of *Helianthemum lippii*, it shows a close similarity with the *Hyparrhenio hirtae*-*Helianthemetum sessiliflorae*, an association described [30] from southern Sicily, where it is localized in the inland paleo-dunes, differing from the Apulian communities for the high cover values of *Hyparrhenia hirta*. The garrigues of *Sileno otitis*-*Helianthemetum lippii* take catenal contact with the psammophilous vegetation of *Euphorbio paraliae*-*Ammophiletea australis* and with the maquis of *Helianthemo sessiliflori*-*Juniperetum macrocarpae* Brullo et al., 2001 [26].

Distribution. It was surveyed along the coastal dune systems of the Ionian arc near Taranto.

12. *Ruto chalepensis-Salvietum trilobae* Biondi & Guerra 2008, Fitosociologia 45 (1) suppl.1: 89 (Appendix B, Table A12)

Holotypus: rel. 5, Table 18, [18].

Characteristic and differential species: *Aurinia saxatilis* subsp. *megalocarpa*, *Coronilla valentina*, *Ruta chalepensis*, *Salvia fruticosa*.

**Figure 18:**
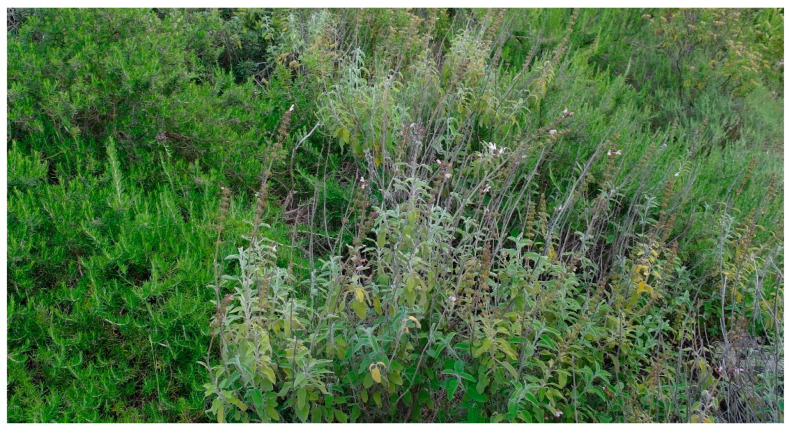
Garrigues of *Ruto chalepensis-Salvietum trilobae*.

Structure and ecology. In the Ionian arc “gravine,” limited to the surfaces characterized by deposits of calcareous debris of various grain sizes, between 150 and 250 m a.s.l., within the lower mesomediterranean bioclimatic belt with an upper dry ombrotype, shrubby vegetation dominated by *Salvia fruticosa* occurs (Figure 18). This eastern Mediterranean species, in Italy, distributed mainly in Apulia and Sicily, grows together with *Ruta chalepensis* and a few other shrubs. This plant community was described by [18] as *Ruto chalepensis*-*Salvietum trilobae* and is differentiated from other associations with *S. fruticosa* by *Coronilla valentina*, *Phagnalon rupestre* subsp. *illyricum,* and *Aurinia saxatilis* subsp. *megalocarpa*. It can be considered as a vicariant of *Salvio trilobae*-*Phlomidetum fruticosae* Barbagallo et al., 1979, the association described from southeastern Sicily, where it grows on the most mesic slopes of the karst canyons of the Hyblean area [31].

**Figure 19:**
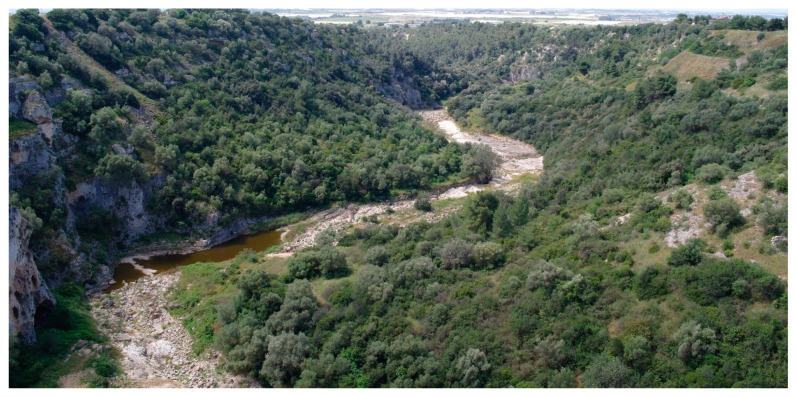
“Gravina” di Castellaneta (Ionian arc).

Distribution. In Apulia, it was observed in the eastern sector of the Ionian arc “gravine” (Figure 19).

13. *Chamaecytiso spinescentis-Cistetum eriocephali* Biondi & Guerra 2008, Fitosociologia 45 (1) suppl.1: 90 (Appendix B, Table A13)

Holotypus: rel. 4, Table 19, [18].

Characteristic and differential species: *Cytisus spinescens*.

Structure and ecology. In the summit parts of the Ionian arc “gravine,” at altitudes ranging between 290 and 360 m a.s.l., within the upper mesomediterranean bioclimatic belt with a lower subhumid ombrotype, garrigue characterized by the dominance of *Cistus creticus* subsp. *eriocephalus*, associated with *Cytisus spinescens*, a shrub with a pulvinar habit, was observed. This vegetation shows a scarce occurrence of shrub species of *Cisto*-*Micromerietea*, probably due to extremely harsh environmental conditions and, therefore, not very favorable for the establishment of garrigues. It was described by [18] as *Chamaecytiso spinescentis-Cistetum eriocephali* and seems linked to windy stands affected by wet draughts coming from the Ionian Sea. In addition, it should be highlighted that *Cytisus spinescens*, a species widespread in the amphiadriatic territories and characteristic of *Cytiso spinescentis*-*Saturejion montanae* Pirone & Tammaro 1997, in other areas of Apulia, shows its optimum in territories characterized by a very mesic bioclimate. This vegetation can be considered a secondary stage of *Teucrio siculi-Quercetum trojanae* Biondi et al., 2004 [18,37].

Distribution. The association is localized in some stands of the Laterza “gravina.”

14. *Erico multiflorae-Halimietum halimifolii* Taffetani & Biondi 1989, Coll. Phytosoc 18: 333 (Appendix B, Table A14)

Holotypus: rel. 8, Table 6, [15].

Characteristic and differential species: *Halimium halimifolium* subsp. *halimifolium*.

**Figure 20:**
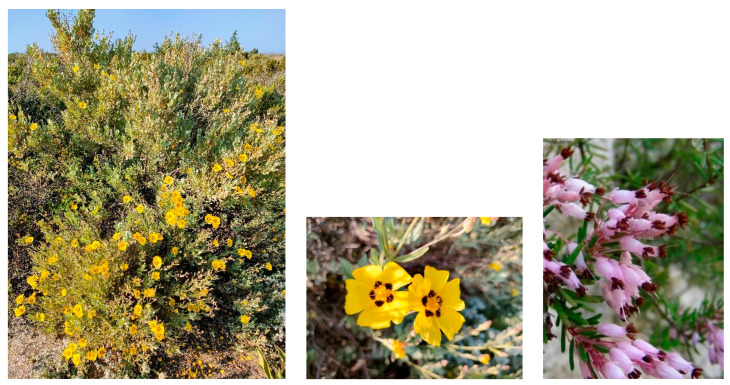
From the left: coastal garrigues of *Erico multiflorae-Halimietum halimifolii*; detail of flowers of *H. halimifolium* subsp. *halimifolium*; *Erica multiflora*.

Structure and ecology. On the inward side of extensive dune complexes, on consolidated sands, in an area characterized by a lower mesomediterranean bioclimate with an upper dry ombrotype, shrubby vegetation dominated by *Halimium halimifolium* subsp. *halimifolium*, a species occurring in the western Mediterranean (Figure 20). It represents a psammophilous garrigue very rich in elements of *Cisto eriocephali-Ericion multiflorae* and *Cisto-Micromerietea*, including, in particular, *Cistus creticus* subsp. *eriocephalus*, *Erica multiflora*, *Rosmarinus officinalis*, *Fumana thymifolia*, *C*. *salviifolius*, and *Thymbra capitata*. This plant community, often covering very extensive surfaces, was described by [15] as *Erico multiflorae*-*Halimietum halimifolii*, an association distributed along the Adriatic coast between Molise and Northern Puglia. For the occurrence of *Halimium halimifolium* subsp. *halimifolium*, this association shows some similarity with those known from the Iberian Peninsula and included in the *Coremation albi* Rothmaler 1943 or *Halimienion halimifolii* Rivas-Martinez & Costa in Rivas-Martinez et al., 1992 [46]. Towards the sea, this community is in catenal contact with the coastal maquis dominated by *Juniperus macrocarpa*, here represented by *Asparago acutifolii*-*Juniperetum macrocarpae* R. & R. Molinier ex O. Bolòs, 1962 [15,47]).

Distribution. Sand coastal areas of northern Apulia, from Bosco Isola di Lesina up to the Molise region.

15. *Helianthemo jonii-Fumanetum thymifoliae* Taffetani & Biondi 1989, Coll. Phytosoc 18: 333 (Appendix B, Table A15)

Holotypus: rel. 2, Table 5, [15].

Characteristic and differential species: *Helianthemum jonium*, *Lotus creticus*, *Verbascum niveum* subsp. *garganicum*.

Structure and ecology. This association replaces *Erico multiflorae*-*Halimietum halimifolii*, which is localized in the flat surfaces with well-consolidated sands, mainly in the more raised parts of dunes with loose sandy soils, affected by lower mesomediterranean bioclimatic belt with a lower dry ombrotype [15]. Although the two associations form complex mosaics, *Helianthemo jonii*-*Fumanetum thymifoliae* is well differentiated by the previous association for the absence or sporadicity of *Halimium halimifolium* subsp. *halimifolium* and *Erica multiflora*, which instead are physiognomically relevant in the other one. Moreover, *Helianthemum jonium* and *Fumana thymifolia* play a very significant role in this association, especially because of their high cover values.

Distribution. This association is well represented in Northern Apulia, especially in the coastal dune complex of the SCI “Duna e Lago di Lesina-Foce del Fortore”. It occurs also in the coastal areas of the Ionian arc near Taranto, in the innermost areas of the dune complexes.

16. *Cistetum salvifolio-clusii* Bartolo, Giardina, Minissale & Spampinato 1987, Boll. Acc. Gioenia Sci. Nat., 20(330): 145 (Appendix B, Table A16)

Holotypus: rel. 11, Table 1, [48].

Characteristic and differential species: *Cistus clusii*.

Structure and ecology. In Apulia, garrigues characterized by the occurrence of *Cistus clusii* were localized in some stands near Lesina Lake, on the Northern side of the Gargano promontory, within the lower mesomediterranean bioclimatic belt with upper dry ombrotype. This vegetation was surveyed by [9], who emphasized that it was linked to sandy soils of the innermost part of the retro-dunal complexes, where *Cistus clusii* grew together with other shrubs of *Cisto*-*Micromerietea*, such as *Rosmarinus officinalis*, *Cistus creticus* subsp. *eriocephalus*, *C. salviifolius*, *Erica multiflora*, *Fumana thymifolia*, etc. According to [48], this plant community was referred to as *Cistetum salvifolio*-*clusii*, an association described from sandy or sandstone substrates of southern Sicily. Floristically and ecologically, the Apulian plant community has strong affinities with that observed in Sicily. As regards *C. clusii*, this species represents a western Mediterranean element since it is distributed in the Iberian Peninsula, Balearic Islands, and Maghreb, while in Italy, it is localized in Sicily and Apulia. On the basis of recent field investigations [49], this species is considered to have nearly disappeared in Apulia for a long time as a consequence of tourist infrastructure. Actually, a small population of *Cistus clusii*, as a result of a reintroduction intervention to remedy its extinction in the wild, can be observed [49]. Other associations characterized by *Cistus clusii* were described from the Iberian Peninsula, such as *Cytiso fontanesii*-*Cistetum clusii* Br.-Bl. & Bolòs 1958, *Anthyllido cytisoidis-Cistetum clusii* Br.-Bl. et al., 1936 corr. O. Bolòs 1967, *Ulici baetici*-*Cistetum clusii* Rivas Goday & Rivas Martinez 1969 corr. Diez Garretas & Asensi 1994, and *Thymo orospedani-Cistetum clusii* F. Valle, Mota & Gomez-Mercado 1988 [46]. It is to be assumed that in Apulia, the association was in catenal contact with *Erico multiflorae-Halimietum halimifolii*, replacing it in more mature edaphic conditions.

Distribution. Originally, the association was recorded from Bosco Isola di Lesina, but, as already highlighted above, it has not been observed in recent times.

*CYTISO SPINESCENTIS-SATUREJION MONTANAE* Pirone and Tammaro 1997, Fitosociologia, 32: 74

Syn: *Artemisio albae-Saturejion montanae* Allegrezza, Biondi, Formica & Ballelli 1997, Fitosociologia 32: 98.

Holotypus: *Osiridi albae-Cistetum cretici* Pirone & Tammaro 1997, Fitosociologia, 32: 75.

**Figure 21:**
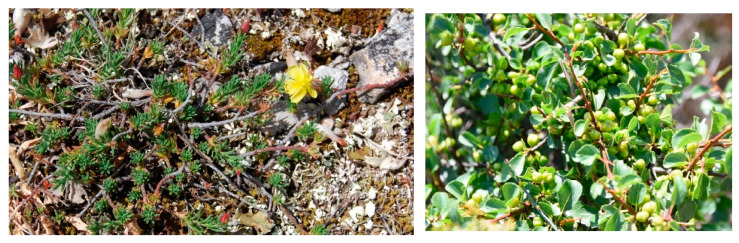
From the left: *Fumana procumbens*, *Rhamnus saxatilis*, char. species of *Cytiso spinescentis*-*Saturejion montanae*.

Characteristic species: *Alyssum diffusum* subsp. *garganicum*, *Centaurea subtilis*, *Cytisus spinescens*, *Fumana procumbens* (Figure 21), *Helianthemum oleandicum* subsp. *incanum*, *Mattiola fruticulosa* subsp. *fruticulosa*, *Rhamnus saxatilis* (Figure 21), *Scabiosa garganica*, and *Satureja montana* subsp. *montana*.

Structure and ecology: According to [50], in the Apennine territories characterized by a mesic bioclimate, the garrigues occurring on calcareous, arenaceous, or conglomeratic substrates are to be included in *Cytiso spinescentis*-*Saturejion montanae*. The plant communities referred to as this alliance are widespread within the sub-Mediterranean variant of the temperate bioclimate, from the mesotemperate to the supratemperate thermotypes. In hilly stands at lower altitudes, this vegetation can also be localized in the meso and supramediterranean belts. Simultaneously with this alliance, in the same journal, other authors [51] described another one, namely *Artemisio albae*-*Saturejion montanae,* and included it in the *Rosmarinetalia officinalis*. Later, Ref. [6] correctly treated the latter as a syntaxonomic synonym of *Cytiso spinescentis*-*Saturejion montanae,* but without giving any floristic or phytogeographical justification, they arranged it within *Festuco hystricis*-*Ononidetea striatae* Rivas-Mart. et al., 2002 and, even more surprisingly, in *Erysimo*-*Jurineetalia bocconei* Brullo 1984, an order endemic to some mountains in Sicily. We completely disagree with this proposed framework, whereas we perfectly agree with [5,50], which includes the alliance in question in the class *Cisto*-*Micromerietea julianae*. Therefore, being the *Artemisio albae-Saturejion montanae* a synonym of *Cytiso spinescentis-Saturejion montanae*, the order *Artemisio albae-Saturejietalia montanae*, of which the aforesaid alliance represents the nomenclatural type, must also be treated as a synonym of *Cisto-Ericetalia manipuliflorae*.

Distribution: This alliance is distributed in the central and southern Apennine up to Apulia and Basilicata.

17. *Centaureo subtilis-Thymetum capitati* Terzi & D’Amico 2006, Quad. Bot. Amb. Appl., 17(2): 68 (Appendix B, Table A17)

Holotypus: rel. 5, Table 3, [17].

Characteristic and differential species: *Lomelosia crenata* subsp. *crenata*, Fu*mana procumbens*, *Anthemis hydruntina* subsp. *hydruntina*, *Scabiosa holosericea*, *Matthiola fruticulosa* subsp. *fruticulose*.

Structure and ecology. In the Murgia “Materana” (Matera, Basilicata), between 350 and 420 m a.s.l., on carbonate substrates represented by Mesozoic limestones dating back to the Cretaceous and partially covered by Plio-Pleistocenic organogenic calcarenitic deposits, a very peculiar garrigue was surveyed, characterized by dwarf shrubs, among which *Thymbra capitata*, *Fumana thymifolia*, *Helianthemum oleandicum* subsp. *incanum*, *Fumana procumbens*, *Satureja montana*, *and Cytisus spinescens* can be mentioned. In these stands, *Centaurea subtilis* plays a significant physiognomical role, a rare endemic species exclusively known from this area of Basilicata and Gargano. Other quite relevant species frequent in this vegetation are some endemics with an Apulo–Lucanian distribution, such as *Leontodon apulus*, *Alyssum diffusum* subsp. *garganicum,* and *Anthemis hydruntina* subsp. *hydruntina*, as well as *Lomelosia crenata* subsp. *crenata*, *Scabiosa holosericea*, and *Matthiola fruticulosa* subsp. *fruticulosa*, showing a wider range. This vegetation was described by [17] as *Centaureo subtilis-Thymetum capitati* and referred to the *Cisto-Ericion* alliance of *Cisto-Micromerietea*. Actually, in this association, the characteristic species of *Cisto eriocephali-Ericion multiflorae* are rare or infrequent, while the mesic floristic contingent of *Cytiso spinescentis-Saturejon montanae* is well represented. In particular, among the species of the latter alliance, *Helianthemum oleandicum* subsp*. incanum*, *Cytisus spinescens*, *Satureja montana* subsp*. montana*, *Rhamnus saxatilis*, and *Alyssum diffusum* subsp. *garganicum* occur. As characteristic and differential species of the association were proposed by [17] *Centaurea subtilis*, *Leontodon apulus,* and *Lomelosia crenata* subsp. *crenata*. In this regard, it must be emphasized that *Centaurea subtilis*, despite having a relevant physiognomic role, is to be included among the diagnostic species of the alliance *Cytiso spinescentis-Saturejion montanae*, while *Leontodon apulus* is quite widespread in many Apulian communities of *Cisto-Micromerietea*, and only *Lomelosia crenata* subsp. *crenata* can be considered a differential in the association. To the latter, *Fumana procumbens*, *Matthiola fruticulosa* subsp*. fruticulosa*, *Scabiosa holosericea*, and *Anthemis hydruntina* subsp*. hydruntina* can be added. In particular, *A. hydruntina* subsp. *hydruntina* is a rare Apulo–Lucanian endemism, which is vicaried on siliceous substrates of the Silan Massif by *A. hydruntina* subsp. *silensis* [52]. This association, localized within the upper mesomediterranean upper dry bioclimatic belt, can be considered as a secondary stage of the thermophilous deciduous oak forests occurring on calcareous substrates.

Distribution. According to current knowledge, the association is localized in the Murgia “Materana” (Basilicata).

18. *Fumano ericifoliae-Centaureetum subtilis* Tomaselli & Costanzo ass. nova hoc loco (Appendix B, Table A18)

Holotypus: rel. 6, Table A18, hoc loco.

Characteristic and differential species: *Fumana ericifolia*, *Scabiosa garganica*, *Hippocrepis comosa*.

**Figure 22:**
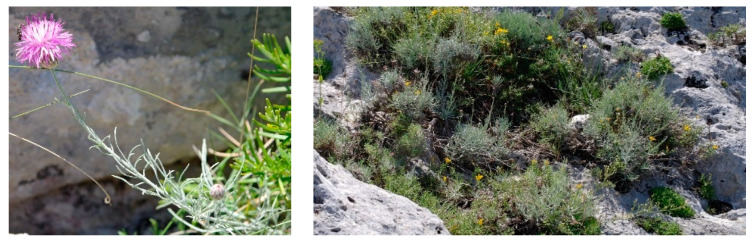
*Centaurea subtilis*; garrigues of *Fumano ericifoliae-Centaureetum subtilis*.

Structure and ecology. On the steep slopes covered by coarse clastic carbonate material, at altitudes between 200 and 700 m a.s.l., a pioneer vegetation characterized by *Centaurea subtilis*, usually showing a dominant role, occurs. In these stands, several dwarf shrubs are frequent, such as *Thymbra capitata*, *Satureja cuneifolia*, *S. montana*, *Fumana ericifolia*, *F. thymifolia*, *Helianthemum oleandicum* subsp *incanum*, *Hippocrepis comosa*, *Rosmarinus officinalis*, etc. Moreover, the occurrence of *Scabiosa garganica*, narrow endemic to the Gargano area, is significant. This plant community shows some similarities with the other association physiognomically dominated by *Centaurea subtilis*, described by [17] from the Murgia Materana (Basilicata) as *Centaureo subtilis*-*Thymetum capitati* (Figure 22). From the floristic and ecological point of view, the latter is well differentiated from the plant community at issue. In fact, the Apulian vegetation is characterized by the occurrence, sometimes with high cover values, of *Fumana ericifolia*, usually growing with *Hippocrepis comosa* and *Scabiosa garganica*, while the diagnostic species of *Centaureo subtilis*-*Thymetum capitati* are completely missing. Therefore, a new association, named *Fumano ericifoliae-Centaureetum subtilis,* is proposed here that can be considered a geographic vicariant of *Centaureo subtilis-Thymetum capitati*. Within this new association, apart from the subass. *typicum*, a new subassociation, *genistetosum michelii* (holotypus: rel. 16, hoc loco) is proposed, which represents a transition aspect towards *Chamaecityso spinescentis-Genistetum michelii*. This vegetation is frequently in serial contact with *Centaureo tenacissimae-Euphorbietum spinosae* or with *Chamaecityso spinescentis-Genistetum michelii* and is dynamically linked to *Cyclamino hederifolii-Querceto ilicis sigmetum*, that is considered as climacic vegetation in this area [37]. This association is widespread within the bioclimatic belts between the upper mesomediterranean and the lower supramediterranean, penetrating, with some isolated patches, even into the upper mesotemperate one.

Distribution. The association has been observed on the southern slope of Gargano promontory (N Puglia).

19. *Chamaecityso spinescentis-Genistetum michelii* De Faveri & Nimis ex Biondi 2000 Coll. Phytosoc 27: 137 (Appendix B, Table A19)

Holotypus: rel. 22, Table 1 [11], designated by [4].

Syn.: *Chamaecityso-Genistetum michelii* De Faveri & Nimis 1982, Ecol. Medit. 8: 89 (Art.5)

Characteristic and differential species: *Genista michelii*.

**Figure 23:**
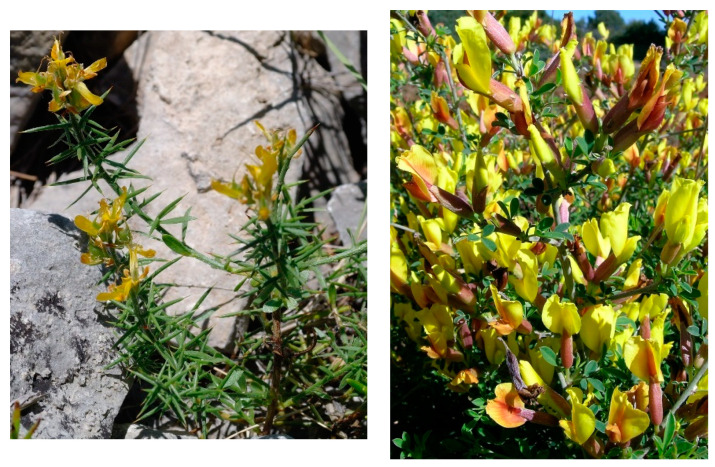
From the left: *Genista michelii*, *Cytisus spinescens* (*Chamaecityso spinescentis-Genistetum michelii*).

Structure and ecology. On shallow stony carbonatic soils, localized on the ridges occurring in the upper part of the valleys frequent in the southern slope of Gargano promontory, between 500 and 700 m a.s.l., a peculiar thorny and cushion-like shrub vegetation occurs. This shrubland is characterized by *Rosmarinus officinalis*, *Thymbra capitata*, *Satureja montana*, *S. cuneifolia*, and *Cytisus spinescens*, which grow together with *Genista michelii*, endemic to Gargano and Central Apennines [43] (Figure 23). This plant community was described by [11] as *Chamaecityso-Genistetum michelii*, but this name, according to Art. 5 of the ICPN, is invalid because the holotype was not designated by the authors. This association was later validated by [4] and included in *Cisto cretici-Ericion manipuliflorae* Horvatić 1958, the alliance of *Cisto-Micromerietea* Oberdorfer 1954. For its floristic set and ecological requirements, *Chamaecityso spinescentis*-*Genistetum michelii* must be more properly referred to as *Cytiso spinescentis-Saturejon montanae*, an alliance represented by *Cytisus spinescens*, *Helianthemum oelandicum* subsp*. incanum*, *Satureja montana* subsp*. montana*, *Rhamnus saxatilis*, *Scabiosa garganica*, and *Centaurea subtilis.* The association seems linked to poorly developed soils in stands strongly affected by humid winds frequently blowing from the sea, while as concerns the bioclimatic conditions, it is localized within the lower supramediterranean and upper mesotemperate with a lower subhumid ombrotype. It can be considered a permanent edaphoclimatic vegetation showing catenal contact with *Fumano ericifoliae-Centaureetum subtilis*, occurring in the habitat with more mature soils and with the dry grasslands of *Stipo austroitalicae-Seslerietum juncifoliae* Di Pietro & Wagensommer 2014, that replaces it on steepest slopes [53]. Previously, *G. michelii* was found in other plant communities of the central Apennines, such as in the subass. *genistetosum michelii* Allegrezza et al., 1997 of *Cephalario leucanthae*-*Saturejetum montanae* Allegrezza et al., 1997, as well as in the subass. *genistetosum michelii* Allegrezza et al., 1997 of *Carici humilis-Seslerietum apenninae* Biondi et al., 1988 [51].

Distribution. Gargano promontory (N Puglia), southern slopes near Monte S. Angelo village.

20. *Centaureo tenacissimae-Euphorbietum spinosae* Minissale, Giusso del Galdo & Brullo ass. nova hoc loco (Appendix B, Table A20)

Holotypus: rel. 2, Table A20, hoc loco.

Characteristic and differential species: *Euphorbia spinosa*, *Centaurea tenacissima*, *Aurinia sinuata*, *Onosma echioides* subsp. *angustifolia*.

Structure and ecology. At altitudes between 400 and 500 m, in hilly and sub-mountain stands characterized by carbonate rock outcrops with shallow soils deposited in correspondence of hollows and cracks of the rock, low-pulvinate vegetation occurs, which is physiognomically dominated by *Satureja cuneifolia* and *Euphorbia spinosa*. Several shrubs of the *Cisto-Micromerietea* class are quite frequent, among them including *Fumana ericoides*, *F. thymifolia*, *Micromeria graeca* subsp*. graeca*, and *Thymbra capitata*. Moreover, the occurrence of some rare or endemic species, such as *Centaurea tenacissima*, *Onosma echioides* subsp*. Angustifolia,* and *Aurinia sinuata*, is very significant. These species allow us to differentiate a new association, proposed as *Centaureo tenacissimae-Euphorbietum spinosae*, which can be included in *Cytiso spinescentis-Saturejon montanae*, as confirmed by the occurrence of *Cytisus spinescens* and *Rhamnus saxatilis*. Similarly to the other associations of the same alliance, previously described for the Central–Southern Apennines [21,50,54], this vegetation also shows mesic requirements, being localized within the upper mesomediterranean lower subhumid bioclimate. As concerns its dynamic position, it is often in contact with the garrigues of *Fumano ericifoliae-Centaureetum subtilis* or, more rarely, with *Chamaecityso spinescentis-Genistetum michelii*.

Distribution. The association was observed on the southern slope of Gargano promontory.

21. *Rhamno saxatili-Saturejetum montanae* Tomaselli, Silletti, Forte 2021, Plant Sociology 58(2): 11 (Appendix B, Table A21)

Holotypus: rel. 18, Table 1, [21].

Characteristic and differential species: *Euphorbia nicaeensis* subsp. *japygica*, *Ruta graveolens*, *Allium apulum*, and *Centaurea brulla*.

**Figure 24:**
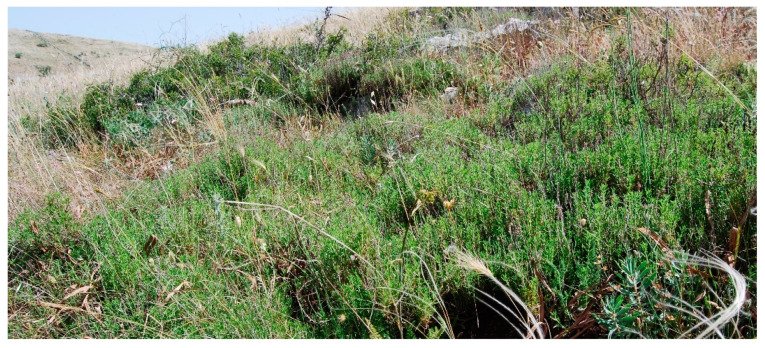
Garrigues of *Rhamno saxatili-Saturejetum montanae* subass. *typicum*.

Structure and ecology: The Alta Murgia Plateau is an area characterized by extensive calcareous outcrops with high stoniness and immature soils, where the garrigue is dominated mainly by *Satureja montana* subsp. *montana*. This vegetation, falling within the upper mesomediterranean lower subhumid bioclimate, occurs at altitudes ranging between 400 and 600 m a.s.l. and was described by [21] as *Rhamno saxatilis-Saturejetum montanae*. Floristically, it is differentiated by *Rhamnus saxatilis*, which usually shows high cover values, along with *Satureja montana* subsp. *montana* (Figure 24). Frequent in this association are also *Ruta graveolens* and some endemic and subendemic taxa, such as *Euphorbia nicaeensis* subsp. *japygica*, *Allium apulum*, and *Centaurea brulla.* Based on some floristic and ecological peculiarities, several subassociations can be distinguished, proposed by [21] as subass. *typicum* and subass. *fumanetosum procumbentis*. The first one is localized on more or less steep and very stony slopes where *Rhamnus saxatilis* has its optimum, often showing a dominant role. The second one occurs on flat or slightly steep surfaces, in stands characterized by soils rich in coarse-grained material, and is differentiated by *Fumana procumbens*, *Odontites luteus* subsp. *luteus*, and *Ornithogalum gussonei*. Previously, from the nearby “gravines” of the Ionian arc, an association physiognomically dominated by *Satureja montana* subsp. *montana* showing close similarities with the plant communities at issue was described by [18]. It was proposed as *Asyneumo limonifoliae-Saturejetum montanae*, which is linked to semi-rupestrian ridge stands, which are quite windy and arid, localized at altitudes between 280 and 400 m a.s.l. It seems to represent a floristically impoverished stage of *Rhamno saxatilis-Saturejetum montanae* and, more than an association, can be considered as a sub-association with markedly thermo-xeric requirements. Therefore, it is here proposed as a subass. *asyneumetosum limonifolium* (Biondi & Guerra 2008) Tomaselli & Forte stat. nov. (Art. 26), having as differential species *Asyneuma limonifolium* subsp. *limonifolium*, *Phagnalon rupestre* subsp. *illyricum*, and *Cistus creticus* subsp*. eriocephalus*.

Distribution. Alta Murgia Plateau (central part of Apulia), and Laterza “gravina” (Ionian Arc, TA).

### 2.3. Syntaxonomical Framework of Cisto-Micromerietea julianae in Apulia

The associations surveyed in the study area and their syntaxonomic arrangement are provided in the following scheme:

*CISTO-MICROMERIETEA JULIANAE* Oberdorfer 1954

*CISTO CRETICI-ERICETALIA MANIPULIFLORAE* Horvatić 1958

*CISTO ERIOCEPHALI-ERICION MULTIFLORAE* Biondi 2000

*Loto commutati-Thymetum capitati* Gèhu, Biondi, Gèhu-Frank & Marchiori 1984

subass. *typicum*

subass. *helichrysetosum italici* Gèhu, Biondi, Gèhu-Frank & Marchiori 1984

subass. *rosmarinetosum officinalis* Gèhu, Biondi, Gèhu-Frank & Marchiori 1984

subass. *plantaginetosum albicantis* Costanzo, Sciandrello & Tomaselli subass. nova

*Dauco gummiferis-Thymelaeetum hirsutae* Costanzo & Tomaselli ass. nova

*Cisto monspeliensis-Sarcopoterietum* spinosi Brullo, Minissale & Spampinato 1997

*Thymbro capitatae-Anthyllidetum japygicae* Costanzo, Tomaselli, Giusso del Galdo & Brullo ass. nova

*Saturejo cuneifoliae-Ericetum manipuliflorae* Brullo, Minissale, Signorello, Spampinato, 1987

*Vicio giacominianae-Helianthemetum jonii* Costanzo, Tomaselli, Giusso del Galdo & Brullo ass. nova

*Cisto eriocephali-Phlomidetum fruticosae* Brullo, Scelsi, Spampinato 2001

*Plantago holostei-Thymbretum capitatae* Tomaselli & Costanzo ass. nova

*Helianthemo jonii-Thymetum capitati* Biondi & Guerra 2008

*Phagnalo annotici-Fumanetum thymifoliae* Biondi 2000

*Sileno otitis-Helianthemetum lippii* Tomaselli & Costanzo ass. nova

*Ruto chalepensis-Salvietum trilobae* Biondi & Guerra 2008

*Chamaecytiso spinescentis-Cistetum eriocephali* Biondi & Guerra 2008

*Erico multiflorae-Halimietum halimifolii* Taffetani & Biondi 1989

*Helianthemo jonii-Fumanetum thymifoliae* Taffetani & Biondi 1989

*Cistetum salvifolio-clusii* Bartolo, Giardina, Minissale & Spampinato 1987

*CYTISO SPINESCENTIS-SATUREJION MONTANAE* Pirone and Tammaro 1997

*Centaureo subtilis-Thymetum capitati* Terzi & D’Amico 2006

*Fumano ericifoliae-Centaureetum subtilis* Tomaselli & Costanzo ass. nova

subass. *typicum*

subass. *genistetosum michelii* Tomaselli & Costanzo subass. nova

*Chamaecytiso spinescentis-Genistetum michelii* De Faveri & Nimis ex Biondi 2000

*Centaureo tenacissimae-Euphorbietum spinosae* Minissale, Giusso del Galdo & Brullo ass. nova

*Rhamno saxatilis-Saturejetum montanae* Tomaselli, Silletti, Forte 2021

subass. *typicum*

subass. *fumanetosum procumbentis* Tomaselli, Silletti, Forte 2021

subass. *asyneumetosum limonifolium* (Biondi & Guerra 2008) Tomaselli & Forte subass. nova

## 3. Materials and Methods

### Data Analysis

We examined the literature on garrigue vegetation in the Puglia region, including the Murgia of Matera in the Basilicata region, because of their proximity and similar phytogeographic characteristics [4,8,9,10,11,12,13,14,17,18,19,21]. All vegetation types attributable to transition or degradation stages were excluded from the analysis (for example, post-fire *Cistus* sp. pl. moors). In addition, original vegetation data were sampled in the field over a period between 1982 and 2023 at homogeneous and relatively stable sites, according to the phytosociological approach [55,56] and to fundamental and updated concepts suggested by [57,58]. The area of original relevés ranges mostly between 50 and 100 m^2^ and is in line with [59], who suggest a minimum area of 50 m^2^ for shrub vegetation (including Mediterranean low scrub); in some cases, larger surfaces (100 to 150 m^2^) were used to detect the floristic diversity of vegetation better. The original relevés collected since 2004 were geocoded using a Global Positioning System and integrated into a geodatabase using ArcGis 9.2. The maps in Figure 8 were produced using this software. All phytosociological relevés from both the literature and original, unpublished surveys were organized in a dataset. The date and locality of the analyzed phytosociological relevés are listed in Appendix E. Specifically, a matrix of 367 (objects) relevés x 265 (variables) species was obtained. No less important, following a review of species’ taxonomic identities conducted both in the field and in herbariums, we corrected several misclassifications. Finally, species with a frequency lower than 2% were removed from the dataset.

Some simple basic statistics were calculated, such as the number of species per relevé and matrix density. Furthermore, a correlation analysis between the area of a relevé and species richness (number of species per relevé), as well as between altitude and species richness, was made.

For the identification of outliers, on the basis of relevé area, the three-sigma test was applied, and 18 relevés were thus removed from the matrix (that is, those relevés with surface > 500 sqm).

Then, in order to visualize the general data structure and to detect the presence of other outliers, the matrix was subjected to different agglomerative methods based on cluster analysis by using different combinations of distance measures and group linkage methods on both presence/absence values and cover values (transformed according to the method proposed by [60]. Based on these analyses, undesirable outliers were removed (e.g., small clusters separated at high hierarchical levels) [61,62] because degraded–altered communities or transitional forms with different types of forest vegetation or scrub (e.g., *Quercetea ilicis*) or grassland (e.g., *Lygeo-Stipetea tenacissimae*).

Finally, a matrix of 292 (objects) relevés × 213 (variables) species was obtained (82 literature and 210 original). For the identification of plant communities, agglomerative hierarchical clustering was performed by using the Flexible-β clustering algorithm with the Bray–Curtis dissimilarity index, according to [62,63], which consider Bray–Curtis dissimilarity, combined with a range of clustering algorithms and especially with Flexible-β, the best choice for heterogeneous (i.e., vegetation) dataset. Beta was set at −0.25, so flexible beta clustering became a space-conserving method [61].

In order to determine the best partition (optimal number of clusters required to separate the dataset into distinct vegetation units), we used the average silhouette width, which assesses whether the clusters are compact and distinct from each other, pointing out the optimum number of clusters and the quality of the entire classification [64,65]. The silhouette width measures the mean dissimilarity of each object from its assigned cluster compared to its dissimilarity from the most similar cluster; high positive silhouette widths are assigned to objects that fit well into their assigned cluster, low silhouette widths to objects that fit poorly into the cluster, while negative silhouette widths indicate objects less dissimilar to another cluster than to the one to which they are assigned. The average silhouette width over all objects provides a measure of the overall goodness-of-clustering [66]. The relevés were also ordinated by means of nonmetric multi-dimensional scaling (NMDS), using the Bray–Curtis coefficient as a dissimilarity measure.

Bioclimatic maps from [24] as well as geo-morphological maps retrieved from the regional geological repository (source: http://webgis.distrettoappenninomeridionale.it/geomorfologica/map_default.phtml; accessed on 15 April 2024) were cross-matched with the phytosociological geodatabase published for the region, assigning to each geo-referenced relevé a series of bioclimatic and edaphic variables. In order to validate the results of multivariate analysis, crosstabs were implemented with the multivariate frequency distribution of the relevés by groups (alliances) and by ecological qualitative variables, i.e., bioclimatic and edaphic (lithology) and Pearson’s Chi-squared test performed to determine the significance of the association between groups and considered variables. Moreover, in order to explore the overall distribution pattern of the relevés based on the altitude, box plots were performed, and the significance of observed differences was tested by the Kruskal–Wallis test.

All computations concerning the numerical classification were performed in R version 4.3.2 (R Core Team [67]) with the ‘vegan’ and ‘cluster’ packages [68,69].

As concerns the floristic nomenclature, we have followed [43,70,71,72,73]. The checklist of the species mentioned in the phytosociological relevés is reported in Appendix A.

The syntaxa names comply with the International Code of Phytosociological Nomenclature (ICPN) [74].

For the bioclimatic characterization of the study area, reference was made according to the bioclimate of Italy [24].

## 4. Conclusions

From the results of the present survey, the diversity, richness, and phytogeographical value of phytocenoses within the investigated area clearly arise. Another important outcome of this study is the separation of the Apulian garrigues in the two alliances, *Cisto eriocephali-Ericion multiflorae* and *Cytiso spinescentis-Saturejion montanae.* Future research could be aimed at expanding investigations on this vegetation to other territories in Italy and the central Mediterranean to better define the geographical and ecological limits of these alliances. As an example, in [6], the authors define *Cisto eriocephali-Ericion multiflorae* as “Thermo-mesomediterranean calcicolous garrigue of the central and southern regions of the Adriatic and Ionian seaboards of the Apennine Peninsula,” without giving information about the southern regions of Thyrrenian seabords and Sicily; this, and other related issues, deserves further investigations.

As regards the conservation aspect, many of the communities described in this paper are characterized by rare, and often endemic species or of relevant phytogeographic and/or conservation value, but only a few of these types are protected as they are included in Annex I of Directive 92/43 EEC (Habitat Directive; European Commission [75,76], such as the *Poterium spinosum* phryganas (habitat 5420, here including *Cisto monspeliensis-Sarcopoterietum spinosi*), and the garrigues established in correspondence of coastal dune systems (habitat 2260, i.e., *Loto commutati-Thymetum capitati*, *Sileno otitis-Helianthemetum lippii*, *Helianthemo jonii-Fumanetum thymifoliae*, *Erico multiflorae-Halimietum halimifolii*, and *Cistetum salvifolio-clusii*). The other communities, although representing vegetation types of high conservation interest, are not included in Annex I. For this reason, a new habitat suitable to be included in Annex I of the Habitat Directive has been recently proposed and described for southern Italy as “Mediterranean and sub-Mediterranean dwarf garrigues with rare and/or endemic species” [77]. This habitat includes thermomediterranean, mesomediterranean, and submediterranean, primary and secondary garrigues, physiognomically dominated by chamaephytes and characterized by the occurrence of rare, endemic or sub-endemic species, or conservation value, such as *Allium apulum*, *Asyneuma limonifolium* subsp*. limonifolium*, *Centaurea brulla*, *C. subtilis*, *Dianthus tarentinus*, *Erica forskalii*, *Fumana scoparia*, *Genista michelii*, *Helianthemum leptophyllum*, *H. jonium*, *H. lippii*, *Leontodon apulus*, *Linum tommasinii*, *Micromeria graeca* subsp. *garganica*, etc. These plant communities correspond to the EUNIS types F6.1 (Western garrigues) and F6.2 (Eastern garrigues), and to the CORINE Biotopes types 32.212 (Thermo-Mediterranean heath garrigues), 32.46 (Lavender garrigues), 32.47 (Thyme, sage, germander and other labiate garrigues), 32.4B (Erica garrigues), 32.4D (*Helianthemum* and *Fumana* garrigues). Many of these garrigues occur in the Apulian territory; some of them fall within the limits of the Natura2000 network or in protected areas, but many others, often covering extensive surfaces, are without any kind of protection and are often subject to various anthropogenic pressures. So, it would be desirable to implement some protection plans specifically addressed to these communities, which are elements characterizing the Apulian landscape, emphasizing their ecological functions and their unique functional and species diversity, in order to maintain the attention of both the scientific and political communities and even with the involvement of local institutions, management bodies, and stakeholders.

## Data Availability

Data are contained within the article.

## Appendix A

Checklist of the taxa occurring in the phytosociological relevés.

*Achnatherum bromoides* (L.) P.Beauv.
*Acinos alpinus* (L.) Moench
*Ajuga chamaepitys* (L.) Schreb.
*Alkanna tinctoria* Tausch subsp. *tinctoria*
*Allium apulum* Brullo, Guglielmo, Pavone & Salmeri
*Allium roseum* L.
*Allium sardoum* Moris
*Allium sphaerocephalon* L.
*Allium subhirsutum* L. subsp. *subhirsutum*
*Alyssum diffusum* Ten. subsp. *garganicum* Španiel, Marhold, N.G.Passal. & Lihová
*Ammophila arenaria* (L.) Link subsp. *arundinacea* (Husn.) H. Lindb.
*Anacamptis coriophora* (L.) R.M. Bateman, Pridgeon & M.W. Chase
*Anagyris foetida* L.
*Anchusa undulata* L. subsp. *hybrida* (Ten.) Bég.
*Andropogon distachyos* L.
*Anethum piperitum* Ucria
*Anthemis hydruntina* E. Groves subsp. *hydruntina*
*Anthyllis hermanniae* L. subsp. *japygica* Brullo & Giusso
*Anthyllis vulneraria* L. subsp. *maura* (Beck) Maire
*Anthyllis vulneraria* L. subsp. *rubriflora* (DC.) Arcang.
*Anthyllis vulneraria* L. subsp. *weldeniana* (Rchb.) Cullen
*Arbutus unedo* L.
*Asparagus acutifolius* L.
*Asphodeline liburnica* (Scop.) Rchb.
*Asphodeline lutea* (L.) Rchb.
*Asphodelus ramosus* L. subsp. *ramosus*
*Asyneuma limonifolium* (L.) Janch. subsp. *limonifolium*
*Aurinia saxatilis* (L.) Desv. subsp. *megalocarpa* (Hausskn.) T.R.Dudley
*Aurinia sinuata* (L.) Griseb.
*Bellardia trixago* (L.) All.
*Bituminaria bituminosa* (L.) C.H.Stirt
*Blackstonia perfoliata* (L.) Huds. subsp. *perfoliata*
*Brachypodium retusum* (Pers.) P. Beauv.
*Bromopsis erecta* (Huds.) Fourr. subsp. *erecta*
*Bupleurum baldense* Turra
*Carduus nutans* L.
*Carex caryophyllea* Latourr.
*Carex distachya* Desf.
*Carex flacca* Schreb. subsp. *erythrostachys* (Hoppe) Holub
*Carex liparocarpos* Gaudin subsp. *liparocarpos*
*Carlina corymbosa* L.
*Catapodium rigidum* (L.) C.E.Hubb.
*Centaurea brulla* Greuter
*Centaurea deusta* Ten. s.l.
*Centaurea sicula* L.
*Centaurea subtilis* Bertol.
*Centaurea tenacissima* (E.Groves) Brullo
*Centaurium erythraea* Rafn
*Centaurium pulchellum* (Sw.) Druce subsp. *pulchellum*
*Centaurium tenuiflorum* (Hoffmanns. & Link) Fritsch
*Cistus clusii* Dunal
*Cistus creticus* L. subsp. *creticus*
*Cistus creticus* L. subsp. *eriocephalus* (Viv.) Greuter & Burdet
*Cistus monspeliensis* L.
*Cistus salviifolius* L.
*Clematis flammula* L.
*Clinopodium suaveolens* (Sm.) Kuntze
*Convolvulus altheoides* L.
*Convolvulus cantabrica* L.
*Convolvulus elegantissimus* Mill.
*Coronilla juncea* L.
*Coronilla scorpioides* (L.) W.D.J.Koch
*Coronilla valentina* L.
*Crepis neglecta* L. subsp. *neglecta*
*Crepis vesicaria* L.
*Crupina crupinastrum* (Moris) Vis.
*Cynanchica aristata* (L.f.) P.Caputo & Del Guacchio subsp. *aristata*
*Cynanchica garganica* (Huter ex Ehrend. & Krendl) P.Caputo & Del Guacchio
*Cytisus infestus* (C.Presl) Guss.
*Cytisus spinescens* C.Presl
*Dactylis glomerata* L. subsp. *hispanica* (Roth) Nyman
*Daphne gnidium* L.
*Dasypyrum villosum* (L.) P.Candargy
*Daucus carota* L. s.l.
*Daucus carota* L. subsp. *gummifer* (Syme) Hook.f.
*Dianthus tarentinus* Lacaita
*Dittrichia viscosa* (L.) Greuter
*Echinops ritro* L.
*Emerus major* Mill. subsp. *emeroides* (Boiss. & Spruner) Soldano & F.Conti
*Erica forskalii* Vitm.
*Erica multiflora* L.
*Eryngium amethystinum* L.
*Eryngium campestre* L.
*Euphorbia characias* L.
*Euphorbia dendroides*
*Euphorbia nicaeensis* All. subsp. *japygica* (Ten.) Arcang.
*Euphorbia segetalis* L.
*Euphorbia spinosa* L.
*Ferula communis* L.
*Festuca circummediterranea* Patzke
*Festuca fasciculata* Forskal.
*Fumana ericifolia* Wallr.
*Fumana laevis* (Cav.) Pau
*Fumana procumbens* (Dunal) Gren. & Godr.
*Fumana scoparia* Pomel
*Fumana thymifolia* (L.) Spach ex Webb
*Galium corrudifolium* Vill.
*Galium lucidum* All.
*Gastridium ventricosum* (Gouan) Schinz & Thell.
*Genista michelii* Spach
*Halimium halimifolium* (L.) Willk. subsp. *halimifolium*
*Hedypnois rhagadioloides* (L.) F.W.Schmidt
*Helianthemum canum*
*Helianthemum croceum* (Desf.) Pers.
*Helianthemum jonium* Lacaita & Grosser
*Helianthemum leptophyllum* Dunal
*Helianthemum nummularium* (L.) Mill.
*Helianthemum oelandicum* (L.) Dum.Cours. subsp. *incanum* (Willk.) G.López
*Helichrysum italicum* (Roth) G.Don subsp. *italicum*
*Helictotrichon convolutum* (C.Presl) Henrard
*Herniaria hirsuta* L. subsp. *hirsuta*
*Hippocrepis ciliata* Willd.
*Hippocrepis comosa* L.
*Hippocrepis glauca* Ten.
*Hyoseris radiata* L.
*Hyparrhenia hirta* (L.) Stapf
*Hypericum spruneri* Boiss.
*Hypochaeris achyrophorus* L.
*Inula verbascifolia* (Willd.) Haussk
*Iris pseudopumila* Tineo
*Juniperus macrocarpa* Sm.
*Juniperus oxycedrus* L.
*Juniperus turbinata* Guss.
*Jurinea mollis* (L.) Rchb. subsp. *mollis*
*Knautia integrifolia* (L.) Bertol. subsp. *integrifolia*
*Koeleria subcaudata* (Asch. & Graebn.) Ujhelyi
*Lagurus ovatus* L.
*Leontodon apulus* (Fiori) Brullo
*Leontodon crispus* Vill.
*Leontodon intermedius* (Fiori) Huter, Porta & Rigo ex Rigo
*Leontodon tuberosus* L.
*Limonium virgatum* (Willd.) Fourr.
*Linum strictum* L.
*Linum tenuifolium* L.
*Linum tommasinii* (Rchb.) Nyman
*Linum trigynum* L.
*Linum usitatissimum* L. subsp. *angustifolium* (Huds.) Thell.
*Lomelosia crenata* (Cirillo) Greuter & Burdet subsp. *crenata*
*Lomelosia crenata* (Cirillo) Greuter & Burdet subsp. *dallaportae* (Boiss.) Greuter & Burdet
*Lonicera implexa* Aiton subsp. *implexa*
*Lotus corniculatus* L.
*Lotus creticus* L.
*Lotus cytisoides* L.
*Lotus herbaceus* (Vill.) Jauzein
*Lotus hirsutus* L.
*Lysimachia arvensis* (L.) U.Manns & Anderb. subsp. *arvensis*
*Macrobriza maxima* (L.) Tzvelev
*Marrubium incanum* Desr.
*Matthiola fruticulosa* (L.) Maire subsp. fruticulosa
*Matthiola sinuata* (L.) W.T.Aiton
*Medicago littoralis* Loisel.
*Medicago minima* (L.) L.
*Melica ciliata* L.
*Micromeria graeca* (L.) Benth. ex Rchb. subsp. *garganica* (Briq.) Guinea
*Micromeria graeca* (L.) Benth. ex Rchb. subsp. *graeca*
*Micromeria juliana* (L.) Benth. ex Rchb.
*Micromeria nervosa* (Desf.) Benth.
*Muscari comosum* (L.) Mill.
*Myrtus communis* L.
*Odontites luteus* (L.) Clairv. subsp. *luteus*
*Odontites vernus* (Bellardi) Dumort. subsp. *serotinus* (Dumort.) Corb.
*Olea europaea* L.
*Oloptum miliaceum* (L.) Röser & H.R.Hamasha
*Onobrychis aequidentata* (Sm.) d’Urv
*Onobrychis alba* (Waldst. & Kit.) Desv. subsp. *alba*
*Onobrychis alba* (Waldst. & Kit.) Desv. subsp. *echinata* (Guss.) P.W. Ball
*Onobrychis caput-galli* (L.) Lam.
*Ononis mitissima* L.
*Ononis pusilla* L.
*Ononis reclinata* L.
*Ononis spinosa* L. subsp. *antiquorum* (L.) Arcang.
*Onosma variegata* L.
*Onosma echioides* (L.) L. subsp. *angustifolia* (Lehm.) Peruzzi & N.G. Passal.
*Onosma echioides* (L.) L. subsp. *echioides*
*Ophrys fusca* Viv.
*Osyris alba* L.
*Pallenis spinosa* (L.) Cass. subsp. *spinosa*
*Pancratium maritimum* L.
*Petrorhagia saxifraga* (L.) Link subsp. *saxifraga*
*Petrosedum ochroleucum* (Chaix) Niederle subsp. *mediterraneum* (L.Gallo) Niederle
*Petrosedum rupestre* (L.) P.V.Heath
*Petrosedum sediforme* (Jacq.) Grulich
*Phagnalon rupestre* (L.) DC. subsp. *illyricum* (H.Lindb.) Ginzb.
*Phillyrea angustifolia* L.
*Phillyrea latifolia* L.
*Phlomis fruticosa* L.
*Picris hieracioides* L.
*Pimpinella tragium* Vill.
*Pinus halepensis* Mill. subsp. *halepensis*
*Pistacia lentiscus* L.
*Pistacia terebinthus* L. subsp. *terebinthus*
*Plantago holosteum* Scop.
*Plantago albicans* L.
*Plantago coronopus* L.
*Plantago lanceolata* L.
*Plantago serraria* L.
*Poa bulbosa* L. subsp. *bulbosa*
*Polygala monspeliaca* L.
*Polygala nicaeensis* Risso ex W.D.J.Koch
*Poterium sanguisorba* L.
*Quercus calliprinos* Webb
*Quercus ilex* L. subsp. *ilex*
*Quercus trojana* Webb subsp. *trojana*
*Ranunculus millefoliatus* Vahl
*Reichardia picroides* (L.) Roth
*Reseda alba* L.
*Reseda phyteuma* L.
*Rhamnus alaternus* L. subsp. *alaternus*
*Rhamnus saxatilis* Jacq.
*Rosmarinus officinalis* L.
*Rubia peregrina* L.
*Ruta chalepensis* L.
*Ruta graveolens* L.
*Sabulina attica* (Boiss. & Sprun.) Dillenb. & Kadereit
*Salvia fruticosa* Mill.
*Sarcopoterium spinosum* (L.) Spach
*Satureja cuneifolia* Ten.
*Satureja montana* L. subsp. *montana*
*Scabiosa garganica* Porta & Rigo ex Wettst.
*Scabiosa holosericea* Bertol.
*Schoenus nigricans* L.
*Scorzonera villosa* Scop. subsp. *columnae* (Guss.) Nyman
*Sedum acre* L.
*Serapias parviflora* Parl.
*Sideritis italica* (Mill.) Greuter & Burdet
*Silene colorata* Poir.
*Silene conica* L.
*Silene italica* (L.) Pers. subsp. italica
*Silene otites* (L.) Wibel
*Silene vulgaris* (Moench) Garcke subsp. *tenoreana* (Colla) Soldano & F. Conti
*Sixalix atropurpurea* (L.) Greuter & Burdet subsp. *maritima* (L.) Greuter & Burdet
*Smilax aspera* L.
*Sonchus bulbosus* (L.) N. Kilian & Greuter subsp. *bulbosus*
*Sporobolus virginicus* (L.) Kunth
*Squilla pancration* Steinh.
*Stachys italica* Mill.
*Stachys major* (L.) Bartolucci & Peruzzi
*Stachys recta* subsp. *subcrenata* (Vis.) Briq.
*Stachys romana* (L.) E.H.L.Krause
*Stipellula capensis* (Thunb.) Röser & H.R.Hamasha
*Sulla capitata* (Desf.) B.H.Choi & H.Ohashi
*Teucrium capitatum* L. subsp. *capitatum*
*Teucrium chamaedrys* L. subsp. *chamaedrys*
*Teucrium flavum* L. subsp. *flavum*
*Teucrium montanum* L.
*Thapsia asclepium* L.
*Thapsia garganica* L.
*Thesium humifusum* DC.
*Thesium humile* Vahl
*Thinopyrum acutum* (DC.) Banfi
*Thinopyrum junceum* (L.) Á.Löve
*Thymbra capitata* (L.) Cav.
*Thymelaea hirsuta* (L.) Endl.
*Thymus spinulosus* Ten.
*Trachynia distachya* (L.) Link
*Trifolium campestre* Schreb.
*Trifolium scabrum* L.
*Trifolium stellatum* L.
*Urospermum dalechampii* (L.) F.W.Schmidt
*Verbascum niveum* Ten. subsp. *garganicum* (Ten.) Murb.
*Verbascum phlomoides* L.
*Vicia giacominiana* Segelb.
*Xeranthemum inapertum* (L.) Mill.

## Appendix B

Phytosociological tables.

**Table A1:** Loto-Thymetum capitati Gèhu, Biondi, Gèhu-Frank & Marchiori 1984; helichrysetosum italici (1–7), rosmarinetosum officinalis (8–9), typicum (10–20), plantaginetosum albicantis (21–28).

Relevé Number	1	2	3	4	5	6	7	8	9	10	11	12	13	14	15	16	17	18	19	20	21	22	23	24	25	26	27	28
**Typus**		X																			X							
**Surface (m^2^)**	10	10	10	9	10	10	100	10	10	80	60	80	70	100	80	100	40	80	80	70	40	50	40	100	50	20	40	60
**Cover (%)**	-	90	90	100	85	90	60	90	85	60	60	50	70	70	80	80	80	60	70	80	70	60	90	90	70	80	85	80
**Altitude (m)**	-	-	-	-	-	-	6	-	-	11	9	7	8	10	5	2	7	9	7	8	3	5	10	4	4	3	5	3
**Exposure**	-	-	-	-	-	-	SO	-	-	SO	S	O	-	-	O	O	-	-	-	-	-	-	O	E	-	-	-	-
**Slope (°)**	-	-	-	-	-	-	3	-	-	5	5	5	-	-	3	3	-	-	-	-	-	-	5	5	-	-	-	-
**Average vegetation height (cm)**	-	-	-	-	-	-	25	-	-	25	30	20	30	30	30	35	30	35	30	30	35	30	60	60	40	50	50	40
**Diagnostic species**																												
*Lotus creticus*	2	2	1	1	1	1	1	1	2	2	2	2	1	1	1	+	1	+	1	1	1	2	1	1	+	1	1	+
*Helichrysum italicum* subsp. *italicum*	3	2	3	4	5	5	3	+	1	.	.	.	+	1	+	2	.	+	+	+	.	+	.	+	.	.	.	.
*Matthiola sinuata*	.	+	+	+	+	+	1	+	+	+	1	+	+	1	1	+	+	1	+	+	.	.	.	.	.	.	.	.
*Coronilla juncea*	.	.	.	.	.	.	.	.	+	.	.	.	2	.	3	.	1	2	1	.	.	.	.	.	.	.	.	.
**Diff. subassociation**																												
*Plantago albicans*	.	.	.	.	.	.	.	.	.	.	+	.	+	.	.	.	.	.	.	.	2	1	1	2	1	+	+	+
***Cisto eriocephali-Ericion multiflorae***																												
*Silene vulgaris* subsp. *tenoreana*	+	1	1	.	1	+	+	2	+	.	.	.	+	1	+	1	+	+	1	+	.	.	.	.	.	.	.	.
*Cistus creticus* subsp. *eriocephalus*	.	.	.	.	.	.	+	.	.	.	.	.	1	.	.	+	.	.	.	.	1	1	.	+	1	2	2	.
*Lotus hirsutus*	.	.	.	.	.	.	.	.	.	.	.	.	1	.	.	+	.	.	.	+	+	+	+	+	.	.	.	.
*Micromeria graeca* subsp. *garganica*	.	.	.	.	.	.	.	.	.	.	.	.	.	.	.	.	.	.	.	.	.	.	.	+	.	.	.	.
***Cisto-Ericetalia manipuliflorae* and *Cisto-Micromerietea***																												
*Thymbra capitata*	3	4	4	2	1	1	3	3	3	4	4	4	4	5	4	5	4	3	4	4	4	3	5	5	4	5	5	4
*Rosmarinus officinalis*	.	+	+	.	.	+	.	4	4	.	.	.	+	1	1	.	1	1	+	.	.	.	.	.	.	.	.	.
*Teucrium capitatum* subsp. *capitatum*	.	.	.	.	.	.	1	.	.	.	1	2	.	.	.	+	.	.	.	.	1	1	+	+	2	1	+	2
*Fumana thymifolia*	.	.	.	.	.	.	.	.	.	1	.	.	+	.	.	+	.	.	.	.	2	3	2	+	2	2	1	2
*Micromeria graeca* subsp. *graeca*	.	.	.	.	.	.	.	.	.	.	.	.	.	.	.	+	.	.	.	+	.	.	.	.	1	+	.	.
*Cistus monspeliensis*	.	.	.	.	.	.	.	.	.	.	.	.	.	.	.	.	.	.	.	.	1	+	2	2	.	.	.	.
*Cistus salviifolius*	.	.	.	.	.	.	.	.	.	.	.	.	.	.	.	.	.	.	.	.	.	.	.	.	+	1	+	2
*Helianthemum jonium*	.	.	.	.	.	.	.	.	+	.	.	.	.	.	.	.	.	.	.	.	.	.	.	.	.	.	.	.
**Other species**																												
*Sixalix atropurpurea* subsp. *maritima*	+	+	+	1	1	+	1	+	1	1	1	+	+	1	.	+	+	.	.	1	.	.	.	.	1	1	+	.
*Pistacia lentiscus*	.	.	.	.	.	.	.	+	+	+	+	+	+	+	+	+	+	.	+	+	+	.	+	1	.	+	.	.
*Reichardia picroides*	.	+	.	.	+	+	1	+	+	1	+	1	+	+	+	.	.	.	+	.	.	.	.	.	+	.	.	.
*Pinus halepensis* subsp. *halepensis*	.	.	.	.	.	.	.	.	.	.	.	.	+	+	.	+	+	+	+	.	+	+	+	.	.	.	.	.
*Sonchus bulbosus* subsp. *bulbosus*	+	1	+	+	1	+	.	+	2	.	.	.	.	.	.	.	.	.	.	.	.	.	.	.	.	.	.	.
*Plantago coronopus*	.	.	.	.	.	.	1	.	.	.	.	1	.	1	.	.	+	+	.	+	.	.	.	.	1	.	.	.
*Cynanchica aristata* subsp. *aristata*	.	.	.	.	.	.	.	.	.	+	+	+	+	.	.	.	.	.	.	.	.	.	+	.	.	+	+	.
*Rubia peregrina*	.	2	+	.	.	.	.	+	1	.	.	.	+	+	.	+	.	.	.	.	.	.	.	.	.	.	.	.
*Cytisus infestus*	.	.	.	.	.	.	.	.	.	.	.	.	.	.	.	.	.	.	.	.	.	.	1	+	+	1	1	1
*Juniperus macrocarpa*	.	.	.	.	.	.	.	.	.	.	.	+	.	+	.	.	+	.	+	+	+	.	.	.	.	.	.	.
*Daphne gnidium*	.	.	.	.	.	.	.	+	.	.	.	.	.	.	.	.	.	.	.	.	.	.	1	+	.	1	1	+
*Sporobolus virginicus*	+	.	+	+	.	.	.	.	.	+	+	.	+	.	.	.	.	.	.	.	.	.	.	.	.	.	.	.
*Crepis vesicaria*	+	.	+	1	.	.	.	.	1	.	.	.	.	+	+	.	.	.	.	.	.	.	.	.	.	.	.	.
*Silene colorata*	.	+	+	.	+	+	.	+	+	.	.	.	.	.	.	.	.	.	.	.	.	.	.	.	.	.	.	.
*Thinopyrum junceum*	.	.	.	.	.	.	.	.	.	.	+	1	.	+	.	.	.	+	.	+	.	.	.	.	.	.	.	.
*Osyris alba*	.	.	.	.	.	.	.	.	.	.	.	.	+	.	.	+	.	.	.	.	.	.	.	.	.	1	+	+
*Phillyrea angustifolia*	.	.	.	.	.	.	.	.	.	.	.	.	.	+	.	+	.	.	.	.	.	.	+	1	.	.	.	.
*Juniperus turbinata*	.	.	.	.	.	.	.	1	+	.	.	.	.	+	.	.	+	.	.	.	.	.	.	.	.	.	.	.
*Ammophila arenaria* subsp. *arundinacea*	.	+	.	.	1	+	.	.	1	.	.	.	.	.	.	.	.	.	.	.	.	.	.	.	.	.	.	.
*Asphodelus ramosus* subsp. *ramosus*	.	.	.	.	.	.	.	.	.	.	.	.	.	.	.	.	.	.	.	.	.	.	.	.	+	+	.	1
*Squilla pancration*	.	.	.	.	.	.	.	.	.	.	.	.	.	.	.	.	.	.	.	.	+	+	.	.	.	.	.	+
*Lonicera implexa* subsp. *implexa*	.	.	.	.	.	.	.	.	.	.	.	.	.	.	.	.	.	.	.	.	.	.	.	.	.	+	+	+
*Quercus ilex* subsp. *ilex*	.	.	.	.	.	.	.	.	.	.	.	.	.	.	.	.	.	.	.	.	.	.	.	.	.	+	+	+
*Smilax aspera*	.	.	.	.	.	.	.	.	.	.	.	.	.	.	.	.	.	.	.	.	.	.	.	.	.	+	+	+
*Limonium virgatum*	.	.	.	+	+	+	.	.	.	.	.	.	.	.	.	.	.	.	.	.	.	.	.	.	.	.	.	.
*Lagurus ovatus*	.	.	.	.	.	+	.	+	+	.	.	.	.	.	.	.	.	.	.	.	.	.	.	.	.	.	.	.
*Brachypodium retusum*	.	.	.	.	.	.	.	.	.	.	.	.	.	.	.	+	.	.	.	.	.	.	.	.	.	.	.	+
*Myrtus communis*	.	.	.	.	.	.	.	.	.	.	.	.	.	.	.	.	.	.	.	+	.	.	.	.	.	.	.	2
*Hippocrepis glauca*	.	.	.	.	.	.	.	.	.	.	.	.	.	.	.	.	.	.	.	.	.	.	+	+	.	.	.	.
*Petrosedum ochroleucum* subsp. *mediterraneum*	.	.	.	.	.	.	.	.	.	.	.	.	1	.	.	1	.	.	.	.	.	.	.	.	.	.	.	.
*Rhamnus alaternus* subsp. *alaternus*	.	.	.	.	.	.	.	.	.	.	.	.	.	.	.	.	.	.	.	.	.	.	.	.	.	+	+	.

**Table A2:** *Dauco gummiferis-Thymelaeetum hirsutae* Costanzo & Tomaselli ass. nova.

Relevé Number	1	2	3	4	5	6	7	8	9
**Typus**						X			
**Surface (m^2^)**	150	50	50	100	100	50	40	70	80
**Cover (%)**	90	90	80	100	95	85	100	85	90
**Altitude (m)**	9	2	2	14	10	8	9	75	30
**Exposure**	-	-	-	O	OSO	S	-	SO	-
**Slope (°)**	-	-	-	10	5	10	-	10	-
**Rockiness**	20	15	15	-	-	-	-	5	5
**Stoniness**	25	20	20	-	-	-	-	10	30
**Average vegetation height (cm)**	60	60	60	-	-	50	50	65	55
**Diagnostic species**									
*Thymelaea hirsuta*	1	1	2	2	2	2	1	+	1
*Daucus carota* subsp. *gummifer*	+	+	+	.	.	1	1	+	+
*Thinopyrum acutum*	+	.	.	+	1	+	+	.	.
***Cisto eriocephali-Ericion multiflorae***									
*Cistus creticus* subsp. *eriocephalus*	2	3	2	.	.	1	2	.	.
*Lotus hirsutus*	1	.	.	1	1	.	.	+	1
*Silene vulgaris* subsp. *tenoreana*	1	.	+	.	+	+	+	.	.
*Dianthus tarentinus*	.	.	.	.	.	.	.	.	1
*Micromeria graeca* subsp. *garganica*	.	.	.	.	.	.	.	2	.
***Cisto-Ericetalia manipuliflorae* and *Cisto-Micromerietea***									
*Thymbra capitata*	4	5	5	4	5	5	4	5	5
*Teucrium capitatum* subsp. *capitatum*	2	2	2	1	+	+	+	.	1
*Fumana thymifolia*	+	.	.	1	+	.	.	.	.
*Helichrysum italicum* subsp. *italicum*	.	.	.	.	.	.	.	.	1
*Micromeria graeca* subsp. *graeca*	1	.	.	.	.	.	.	.	.
*Cistus monspeliensis*	.	.	.	+	.	.	.	.	.
*Satureja cuneifolia*	.	.	.	.	.	.	.	+	.
*Phlomis fruticosa*	.	.	.	.	.	.	.	+	.
*Asyneuma limonifolium* subsp. *limonifolium*	.	.	.	.	.	.	.	+	.
**Other species**									
*Dactylis glomerata* subsp. *hispanica*	+	.	1	+	1	+	1	.	+
*Anthyllis vulneraria* subsp. *rubriflora*	2	.	.	.	.	1	+	+	+
*Reichardia picroides*	.	.	.	+	+	.	.	+	+
*Sixalix atropurpurea* subsp. *maritima*	+	.	.	+	.	1	+	+	.
*Pinus halepensis* subsp. *halepensis*	.	.	+	+	.	.	.	+	+
*Brachypodium retusum*	2	1	+	.	.	.	2	2	.
*Myrtus communis*	1	+	1	.	.	+	.	+	.
*Phillyrea latifolia*	2	1	1	1	+	.	.	.	.
*Plantago serraria*	.	.	.	+	+	.	.	1	1
*Pistacia lentiscus*	.	.	.	2	2	1	1	.	.
*Pallenis spinosa* subsp. *spinosa*	1	.	.	+	+	.	+	.	.
*Carlina corymbosa*	.	.	.	1	+	.	+	.	+
*Cytisus infestus*	.	+	1	2	.	.	.	.	.
*Lotus creticus*	.	.	.	.	+	2	+	.	.
*Knautia integrifolia* subsp. *integrifolia*	.	.	.	.	.	.	.	+	+
*Plantago coronopus*	.	.	.	+	.	+	.	.	.
*Juniperus macrocarpa*	.	.	.	.	.	1	+	.	.
*Asphodelus ramosus* subsp. *ramosus*	.	.	.	.	.	.	.	+	+
*Squilla pancration*	.	.	.	+	.	.	.	+	.

**Table A3:** *Cisto monspeliensis-Sarcopoterietum spinosi* Brullo, Minissale & Spampinato 1997.

Relevé Number	1	2	3	4	5	6	7	8	9	10	11	12	13
**Typus**	X												
**Surface (m^2^)**	200	200	150	300	150	100	100	40	30	40	50	40	50
**Cover (%)**	85	70	70	90	70	100	100	100	100	90	100	100	20
**Altitude (m)**	2	2	2	2	2	2	2	2	2	3	2	2	1
**Average vegetation height (cm)**	70	70	60	40	60	-	-	40	40	40	50	50	-
**Diagnostic species**													
*Sarcopoterium spinosum*	3	3	2	2	2	3	3	3	3	4	4	3	2
***Cisto eriocephali-Ericion multiflorae***													
*Micromeria graeca* subsp. *garganica*	.	.	.	.	.	1	+	1	1	2	1	1	.
*Cistus creticus* subsp. *eriocephalus*	.	.	.	.	.	.	.	1	+	.	+	.	.
*Lotus hirsutus*	.	.	.	.	.	.	.	.	.	1	.	1	.
*Silene vulgaris* subsp. *tenoreana*	.	.	.	.	+	.	.	.	.	.	.	.	.
***Cisto-Ericetalia manipuliflorae* and *Cisto-Micromerietea***													
*Thymbra capitata*	2	1	2	3	3	2	3	3	2	1	3	4	3
*Teucrium capitatum* subsp. *capitatum*	+	+	1	+	+	.	.	1	1	2	1	2	1
*Fumana thymifolia*	.	+	+	.	1	1	+	+	2	2	2	1	1
*Cistus monspeliensis*	2	2	3	2	2	3	2	.	.	.	+	+	3
*Helichrysum italicum* subsp. *italicum*	+	+	.	1	2	.	.	1	2	3	2	2	.
*Micromeria graeca* subsp. *graeca*	+	+	+	+	+	.	.	+	1	.	.	.	+
*Fumana laevis*	.	.	.	.	.	.	.	+	.	1	2	1	.
*Cistus salviifolius*	.	.	.	.	.	.	.	.	.	1	1	2	.
**Other species**													
*Pistacia lentiscus*	1	1	2	1	+	2	2	+	1	1	+	1	2
*Dactylis glomerata* subsp. *hispanica*	+	+	1	1	+	1	1	.	1	1	+	+	.
*Brachypodium retusum*	2	1	1	2	+	1	+	1	.	2	1	1	.
*Squilla pancration*	+	+	+	+	+	.	+	.	+	2	1	1	2
*Asparagus acutifolius*	+	.	+	+	+	.	.	+	+	1	+	1	+
*Myrtus communis*	1	+	1	1	.	1	+	.	.	1	.	.	+
*Linum strictum*	.	.	+	+	+	+	.	.	.	+	+	+	.
*Carlina corymbosa*	1	+	+	+	.	.	.	+	+	.	.	.	+
*Cytisus infestus*	+	+	.	+	+	1	2	.	.	.	.	.	+
*Carex flacca* subsp. *erythrostachys*	+	+	.	+	+	+	1	.	.	.	.	.	.
*Daucus carota* s.l.	+	+	+	+	+	1	.	.	.	.	.	.	.
*Reichardia picroides*	.	.	.	+	+	+	+	+	.	.	.	.	+
*Pallenis spinosa* subsp. *spinosa*	.	+	.	+	.	.	.	.	.	+	1	+	.
*Dittrichia viscosa*	.	.	+	+	+	+	.	.	.	.	.	.	+
*Plantago serraria*	.	.	.	+	+	1	1	.	.	.	.	.	1
*Sixalix atropurpurea* subsp. *maritima*	+	+	+	.	.	.	.	1	.	.	.	+	.
*Trifolium stellatum*	+	+	+	.	+	.	.	.	.	.	.	.	.
*Phillyrea angustifolia*	+	+	+	.	.	.	.	.	.	.	.	.	+
*Stachys major*	.	.	.	+	+	+	.	.	.	.	.	.	1
*Lagurus ovatus*	.	+	+	+	.	.	.	.	.	.	.	.	.
*Olea europaea*	.	.	.	+	.	.	.	.	.	.	+	1	.
*Plantago coronopus*	.	+	+	+	.	.	.	.	.	.	.	.	.
*Schoenus nigricans*	.	.	+	.	.	+	+	.	.	.	.	.	.
*Lotus creticus*	.	.	.	.	.	.	.	1	.	+	1	.	.
*Alkanna tinctoria* subsp. *tinctoria*	.	.	+	.	.	.	.	+	.	+	.	.	.
*Trifolium campestre*	.	.	+	.	.	.	.	.	+	.	.	.	.
*Brachypodium distachyon*	+	.	.	+	.	.	.	.	.	.	.	.	.
*Centaurium erythraea*	+	.	.	.	.	.	.	.	.	+	.	.	.
*Limonium virgatum*	.	.	+	+	.	.	.	.	.	.	.	.	.
*Phillyrea latifolia*	.	.	.	.	.	1	+	.	.	.	.	.	.
*Smilax aspera*	.	+	.	.	+	.	.	.	.	.	.	.	.
*Teucrium chamaedrys* subsp. *chamaedrys*	.	.	.	.	.	.	.	.	.	1	+	.	.
*Anthyllis vulneraria* subsp. *rubriflora*	.	.	.	.	.	.	+	.	.	.	.	.	.

**Table A4:** *Thymbro capitatae-Anthyllidetum japygicae* Costanzo, Tomaselli, Giusso del Galdo & Brullo ass. nova.

Relevé Number	1	2	3	4	5	6	7	8	9	10	11	12	13	14
**Typus**										X				
**Surface (m^2^)**	100	80	100	100	100	100	100	100	100	100	100	50	50	100
**Cover (%)**	80	90	95	95	95	100	100	100	90	90	80	90	80	90
**Altitude (m)**	5	6	5	7	6	4	5	5	5	5	5	5	5	5
**Exposure**	-	S	-	-	-	-	-	-	-	-	-	-	-	-
**Slope (°)**	-	5	-	-	-	-	-	-	-	-	-	-	-	-
**Diagnostic species**														
*Anthyllis hermanniae* subsp. *japygica*	1	+	1	3	2	3	2	3	2	3	2	2	3	3
***Cisto eriocephali-Ericion multiflorae***														
*Lotus hirsutus*	.	.	.	+	+	.	.	.	.	+	+	.	.	+
*Micromeria graeca* subsp. *garganica*	.	.	+	+	1	.	.	+	.	.	.	.	.	.
*Cistus creticus* subsp. *eriocephalus*	.	.	.	.	.	.	.	.	.	2	.	+	.	+
***Cisto-Ericetalia manipuliflorae* and *Cisto-Micromerietea***														
*Rosmarinus officinalis*	3	5	4	2	1	4	4	3	4	4	4	3	3	3
*Thymbra capitata*	3	1	2	2	3	+	1	2	2	2	2	3	2	1
*Teucrium capitatum* subsp. *capitatum*	1	+	+	+	+	+	+	+	+	+	1	+	1	+
*Fumana thymifolia*	2	1	1	1	+	+	+	.	2	1	2	1	1	2
*Cistus salviifolius*	2	+	1	+	+	+	.	.	1	2	+	2	+	2
*Cistus monspeliensis*	.	.	2	2	3	2	2	1	2	2	1	3	1	1
**Other species**														
*Pistacia lentiscus*	1	+	+	1	1	+	1	1	.	+	.	1	+	.
*Cytisus infestus*	.	.	.	.	1	1	.	+	1	2	1	+	+	1
*Myrtus communis*	+	1	1	1	1	+	+	1	.	.	.	.	.	.
*Crepis vesicaria*	+	+	+	+	.	.	.	.	.	+	+	.	+	+
*Sixalix atropurpurea* subsp. *maritima*	1	+	.	.	.	+	.	+	.	+	.	.	+	+
*Carex flacca* subsp. *erythrostachys*	.	.	1	1	+	1	+	1	.	.	.	.	.	.
*Cynanchica aristata* subsp. *aristata*	+	.	+	.	+	.	.	.	.	.	+	+	.	+
*Petrosedum ochroleucum* subsp. *mediterraneum*	.	.	.	.	.	.	.	.	+	1	1	1	+	+
*Anthyllis vulneraria* subsp. *rubriflora*	.	.	+	1	+	+	.	+	.	.	.	.	.	.
*Dactylis glomerata* subsp. *hispanica*	.	.	+	+	+	+	.	+	.	.	.	.	.	.
*Juniperus turbinata*	+	+	+	.	.	.	+	+	.	.	.	.	.	.
*Reichardia picroides*	+	+	.	.	.	+	+	+	.	.	.	.	.	.
*Odontites luteus* subsp. *luteus*	.	.	.	.	.	.	.	.	.	+	+	+	+	+
*Sonchus bulbosus* subsp. *bulbosus*	.	.	.	.	.	.	.	+	+	+	+	+	.	.
*Brachypodium retusum*	.	.	+	+	.	+	.	+	.	.	.	.	.	.
*Squilla pancration*	.	.	.	.	.	+	+	.	.	+	+	.	.	.
*Pinus halepensis* subsp. *halepensis*	+	+	.	.	+	.	+	.	.	.	.	.	.	.
*Lotus cytisoides*	.	.	.	.	.	.	.	.	+	+	+	.	.	.
*Carlina corymbosa*	.	.	.	+	1	.	.	+	.	.	.	.	.	.
*Plantago serraria*	.	.	.	.	.	.	+	+	.	.	.	.	.	.
*Smilax aspera*	.	.	.	+	.	.	+	.	.	.	.	.	.	.

**Table A5:** *Saturejo cuneifoliae-Ericetum manipuliflorae* Brullo, Minissale, Signorello, Spampinato, 1987.

Relevé Number	1	2	3	4	5	6	7	8	9	10	11	12	13	14	15	16	17
**Typus**							X										
**Surface (m^2^)**	100	90	50	20	50	100	60	80	100	50	100	100	100	80	40	100	100
**Cover (%)**	100	100	100	90	90	90	100	100	100	100	90	95	95	90	80	90	95
**Altitude (m)**	14	14	16	16	20	20	5	5	15	15	5	12	12	14	10	10	12
**Rockiness**	-	-	-	-	-	-	-	-	-	-	5	5	5	5	10	5	5
**Stoniness**	-	-	-	-	-	-	-	-	-	-	20	15	15	15	10	15	15
**Average vegetation height (cm)**	-	-	-	-	-	-	-	-	-	-	70	70	70	50	60	60	70
**Diagnostic species**																	
*Erica forskalii*	3	2	3	3	3	4	3	4	3	3	4	4	4	3	3	4	5
*Lotus herbaceus*	2	2	1	.	3	1	2	+	2	1	.	.	.	.	.	.	.
***Cisto eriocephali-Ericion multiflorae***																	
*Cistus creticus* subsp. *eriocephalus*	1	1	+	2	3	2	2	2	2	2	.	1	+	1	+	1	2
*Lotus hirsutus*	3	1	1	.	.	1	1	1	+	+	.	.	.	.	.	.	.
*Dianthus tarentinus*	.	.	.	.	.	+	+	+	.	.	.	.	.	.	.	.	.
***Cisto-Ericetalia manipuliflorae* and *Cisto-Micromerietea***																	
*Satureja cuneifolia*	1	+	1	2	2	1	2	1	2	2	1	2	1	1	2	+	+
*Cistus salviifolius*	2	2	2	+	1	2	2	2	1	2	2	2	2	1	1	2	2
*Thymbra capitata*	+	+	+	2	+	1	1	1	.	1	+	3	2	3	2	1	+
*Fumana thymifolia*	.	+	+	2	+	1	1	.	+	+	.	2	1	1	1	3	3
*Rosmarinus officinalis*	+	.	1	.	2	2	2	2	3	2	.	3	4	4	2	3	3
*Cistus monspeliensis*	.	1	2	.	.	1	2	1	2	2	.	.	.	.	.	.	.
*Micromeria graeca* subsp. *graeca*	.	.	.	.	.	.	.	.	.	.	.	1	1	+	+	.	.
*Teucrium capitatum* subsp. *capitatum*	.	.	.	.	.	.	.	.	.	.	.	+	+	+	.	.	.
**Other species**																	
*Brachypodium retusum*	+	+	+	2	1	1	2	2	2	1	.	+	+	1	1	+	.
*Dactylis glomerata* subsp. *hispanica*	.	.	.	1	+	+	1	1	+	.	1	+	1	1	+	+	+
*Arbutus unedo*	2	3	2	1	.	1	+	1	1	1	.	.	.	.	.	2	+
*Schoenus nigricans*	.	.	.	.	1	1	1	+	1	1	+	.	+	+	+	+	.
*Myrtus communis*	1	2	2	.	2	1	2	1	2	1	.	1	.	.	.	.	.
*Carex flacca* subsp. *erythrostachys*	.	.	.	1	+	+	1	.	+	+	1	.	.	.	.	+	1
*Daucus carota* s.l.	.	.	.	1	+	.	+	+	+	+	.	+	+	+	.	.	.
*Pistacia lentiscus*	1	3	2	.	1	1	+	1	+	.	.	.	.	.	.	+	.
*Festuca circummediterranea*	.	.	.	.	.	1	1	+	.	+	.	1	+	1	+	.	.
*Pinus halepensis* subsp. *halepensis*	.	.	1	.	.	1	.	.	1	1	.	+	+	+	.	.	+
*Cynanchica aristata* subsp. *aristata*	.	.	.	1	1	1	+	+	+	1	.	.	.	.	.	.	.
*Lotus corniculatus*	.	.	+	.	1	1	1	+	1	1	.	.	.	.	.	.	.
*Odontites luteus* subsp. *luteus*	.	.	.	.	.	.	.	.	.	.	.	1	+	1	1	2	2
*Lonicera implexa* subsp. *implexa*	+	+	1	.	.	+	.	.	+	+	.	.	.	.	.	.	.
*Odontites vernus* subsp. *serotinus*	.	.	.	1	+	1	1	.	1	1	.	.	.	.	.	.	.
*Ononis spinosa* subsp. *antiquorum*	.	.	.	.	2	1	1	2	1	1	.	.	.	.	.	.	.
*Osyris alba*	.	.	.	.	1	1	+	+	1	1	.	.	.	.	.	.	.
*Phillyrea angustifolia*	1	1	2	.	.	+	+	.	+	.	.	.	.	.	.	.	.
*Quercus calliprinos*	1	2	2	.	1	+	.	.	.	1	.	.	.	.	.	.	.
*Reichardia picroides*	.	.	.	.	.	.	.	.	.	.	.	+	+	+	+	+	+
*Sixalix atropurpurea* subsp. *maritima*	.	.	.	+	+	.	+	.	+	+	.	.	.	.	.	.	.
*Leontodon tuberosus*	.	.	.	.	.	.	.	.	.	.	1	.	+	+	+	.	.
*Picris hieracioides* subsp. *hieracioides*	.	.	.	.	+	.	+	+	.	+	.	.	.	.	.	.	.
*Asparagus acutifolius*	.	.	+	.	.	.	+	+	.	1	.	.	.	.	.	.	.
*Juniperus turbinata*	.	.	.	.	.	+	.	+	+	+	.	.	.	.	.	.	.
*Petrosedum rupestre*	.	.	.	.	.	.	.	.	.	.	.	+	+	+	+	.	.
*Plantago coronopus*	.	.	.	.	+	.	+	+	+	.	.	.	.	.	.	.	.
*Cytisus infestus*	+	.	1	.	.	.	.	+	.	.	.	.	.	.	.	.	.
*Petrosedum sediforme*	.	.	.	.	.	.	+	+	.	+	.	.	.	.	.	.	.
*Rubia peregrina*	.	.	.	.	.	+	.	.	+	1	.	.	.	.	.	.	.
*Silene italica* subsp. *italica*	.	.	.	.	.	.	.	.	.	.	.	+	+	+	.	.	.
*Daphne gnidium*	+	.	1	.	.	.	.	.	.	.	.	.	.	.	.	.	.
*Macrobriza maxima*	.	+	+	.	.	.	.	.	.	.	.	.	.	.	.	.	.
*Smilax aspera*	+	+	.	.	.	.	.	.	.	.	.	.	.	.	.	.	.
*Juniperus macrocarpa*	.	.	.	.	.	.	.	.	.	.	+	.	.	.	.	.	.
*Olea europaea*	.	.	.	.	.	.	.	.	.	.	+	.	.	.	.	.	.
*Phillyrea latifolia*	.	.	.	.	.	.	.	.	.	.	+	.	.	.	.	.	.

**Table A6:** *Vicio giacominianae-Helianthemetum jonii* Costanzo, Tomaselli, Giusso & Brullo ass. nova.

Relevé Number	1	2	3	4	5	6	7	8	9	10	11	12	13
**Typus**								X					
**Surface (m^2^)**	80	70	50	60	100	50	50	10	50	50	50	100	50
**Cover (%)**	60	50	50	70	80	80	90	100	90	90	80	60	80
**Altitude (m)**	45	70	10	15	80	18	20	24	18	25	20	20	30
**Exposure**	E	SE	S	S	-	S	S	S	S	S	-	-	-
**Slope (°)**	10	10	5	5	-	5	5	10	10	10	-	-	-
**Rockiness**	30	35	45	25	20	-	-	-	-	-	-	-	-
**Stoniness**	10	15	5	5	-	-	-	-	-	-	-	-	-
**Average vegetation height (cm)**	20	20	15	15	60	-	-	-	-	-	-	-	-
**Diagnostic species**													
*Helianthemum jonium*	1	2	1	1	1	2	2	2	1	1	2	1	1
*Vicia giacominiana*	+	.	+	+	.	.	1	+	+	+	+	.	.
*Centaurea tenacissima*	.	.	.	.	.	.	.	.	.	+	2	.	.
***Cisto eriocephali-Ericion multiflorae***													
*Cistus creticus* subsp. *eriocephalus*	1	1	.	+	+	+	1	3	3	2	.	1	2
*Dianthus tarentinus*	1	1	.	.	+	1	1	2	1	+	+	+	1
*Micromeria graeca* subsp. *garganica*	+	1	.	+	1	.	.	.	.	.	.	.	.
*Lotus hirsutus*	.	.	.	.	.	.	.	+	.	.	.	+	.
*Phagnalon rupestre* subsp. *illyricum*	.	.	.	.	.	.	.	.	.	.	.	.	+
***Cisto-Ericetalia manipuliflorae* and *Cisto-Micromerietea***													
*Thymbra capitata*	2	2	4	2	4	3	3	3	3	4	1	2	1
*Fumana thymifolia*	2	3	+	1	1	2	2	3	2	2	1	2	1
*Satureja cuneifolia*	4	4	2	4	1	3	2	2	3	2	2	3	2
*Leontodon apulus*	+	+	+	+	.	1	2	2	1	+	+	1	1
*Asyneuma limonifolium* subsp. *limonifolium*	+	+	+	.	.	+	1	1	1	1	1	.	2
*Teucrium capitatum* subsp. *capitatum*	+	.	+	+	.	.	.	.	+	+	+	1	+
*Micromeria nervosa*	.	.	.	.	.	+	1	2	1	1	1	1	1
*Phlomis fruticosa*	.	.	.	.	.	.	2	2	2	1	+	1	1
*Hippocrepis glauca*	.	.	.	.	.	1	+	1	+	.	.	+	+
*Cistus salviifolius*	+	1	.	.	.	.	.	.	.	.	.	1	1
*Helichrysum italicum* subsp. *italicum*	.	.	.	.	.	+	+	+	.	+	.	.	.
*Rosmarinus officinalis*	.	.	.	.	.	.	.	.	.	.	.	2	2
**Other species**													
*Brachypodium retusum*	.	1	1	+	2	1	.	2	+	2	+	.	1
*Dactylis glomerata* subsp. *hispanica*	.	.	.	+	+	2	2	2	+	1	+	+	1
*Daucus carota* s.l.	.	.	+	+	+	+	+	+	+	+	+	.	+
*Festuca circummediterranea*	1	+	.	.	.	2	2	2	1	1	1	2	2
*Hyparrhenia hirta*	.	+	+	+	+	+	.	+	+	1	1	.	.
*Lotus cytisoides*	.	+	.	1	+	1	+	1	+	+	+	.	.
*Reichardia picroides*	+	.	+	+	+	.	1	+	.	+	1	.	+
*Anthyllis vulneraria* subsp. *maura*	.	.	.	.	.	1	+	1	1	+	+	+	+
*Asparagus acutifolius*	.	+	+	.	+	.	1	+	+	+	.	.	+
*Bituminaria bituminosa*	+	.	+	+	.	.	.	+	+	+	+	+	.
*Petrosedum ochroleucum* subsp. *mediterraneum*	.	.	.	1	.	.	+	1	+	+	+	+	+
*Crepis vesicaria*	.	.	+	+	.	+	+	+	+	+	.	.	.
*Koeleria subcaudata*	.	.	.	.	.	1	.	2	+	+	+	1	+
*Sabulina attica*	.	.	.	.	.	1	+	1	+	.	+	+	1
*Cynanchica aristata* subsp. *aristata*	+	+	+	1	.	.	.	.	.	.	.	+	+
*Euphorbia spinosa*	.	.	.	.	.	.	.	1	1	+	3	2	3
*Silene italica* subsp. *italica*	.	.	.	.	.	+	2	1	.	.	+	+	+
*Carex flacca* subsp. *erythrostachys*	.	.	.	.	1	+	+	+	+	.	.	.	.
*Squilla pancration*	.	.	.	.	2	.	+	.	.	+	.	+	1
*Linum usitatissimum* subsp. *angustifolium*	+	+	+	.	+	.	.	.	.	.	.	.	.
*Pinus halepensis* subsp. *halepensis*	+	+	.	+	+	.	.	.	.	.	.	.	.
*Trifolium scabrum*	+	+	1	1	.	.	.	.	.	.	.	.	.
*Bromopsis erecta* subsp. *erecta*	.	.	.	.	.	2	+	+	.	.	.	.	.
*Centaurium tenuiflorum*	+	+	+	.	.	.	.	.	.	.	.	.	.
*Hypochaeris achyrophorus*	.	+	.	+	+	.	.	.	.	.	.	.	.
*Plantago holosteum*	.	.	.	.	.	2	1	1	.	.	.	.	.
*Serapias parviflora*	.	.	.	.	.	+	.	+	+	.	.	.	.
*Stachys romana*	.	.	+	+	+	.	.	.	.	.	.	.	.
*Anthyllis vulneraria* subsp. *rubriflora*	+	.	.	.	+	.	.	.	.	.	.	.	.
*Euphorbia segetalis*	.	.	.	.	.	+	1	.	.	.	.	.	.
*Olea europaea*	.	+	.	.	+	.	.	.	.	.	.	.	.
*Onobrychis alba* subsp. *echinata*	.	.	.	.	.	.	.	.	.	.	.	+	+
*Petrorhagia saxifraga* subsp. *saxifraga*	+	1	.	.	.	.	.	.	.	.	.	.	.
*Pistacia lentiscus*	.	.	.	.	.	.	+	.	+	.	.	.	.
*Sixalix atropurpurea* subsp. *maritima*	.	.	.	1	+	.	.	.	.	.	.	.	.

**Table A7:** *Cisto eriocephali-Phlomidetum fruticosae* Brullo, Scelsi, Spampinato 2001.

Relevé Number	1	2	3	4	5
**Typus**					
**Surface (m^2^)**	60	60	50	50	50
**Cover (%)**	90	95	80	90	90
**Altitude (m)**	60	45	50	45	50
**Exposure**	E	E	E	S	SO
**Slope (°)**	5	5	5	10	10
**Rockiness**	5	5	10	5	5
**Stoniness**	-	-	10	15	15
**Average vegetation height (cm)**	70	60	50	120	100
**Diagnostic species**					
*Phlomis fruticosa*	4	4	3	5	5
***Cisto eriocephali-Ericion multiflorae***					
*Cistus creticus* subsp. *eriocephalus*	3	3	4	2	+
*Micromeria graeca* subsp. *garganica*	1	+	1	2	1
***Cisto-Ericetalia manipuliflorae* and *Cisto-Micromerietea***					
*Satureja cuneifolia*	1	1	2	2	+
*Thymbra capitata*	2	2	1	1	.
*Fumana thymifolia*	.	+	1	+	.
*Helianthemum jonium*	.	+	1	+	.
*Asyneuma limonifolium* subsp. *limonifolium*	.	.	1	+	+
*Leontodon apulus*	+	.	1	+	.
*Teucrium capitatum* subsp. *capitatum*	+	+	.	.	.
**Other species**					
*Asparagus acutifolius*	+	+	+	+	1
*Pistacia lentiscus*	+	+	+	+	2
*Reichardia picroides*	+	+	+	+	1
*Anthyllis vulneraria* subsp. *rubriflora*	+	+	1	+	.
*Bituminaria bituminosa*	1	1	+	.	+
*Brachypodium retusum*	2	1	2	1	.
*Sixalix atropurpurea* subsp. *maritima*	+	+	+	+	.
*Vicia giacominiana*	+	+	.	.	+
*Carex flacca* subsp. *erythrostachys*	.	+	1	.	+
*Squilla pancration*	.	1	.	1	+
*Olea europaea*	.	+	+	.	+
*Petrorhagia saxifraga* subsp. *saxifraga*	+	+	+	.	.
*Stachys major*	1	+	+	.	.
*Cynanchica aristata* subsp. *aristata*	.	+	+	.	.
*Crepis vesicaria*	+	+	.	.	.
*Daucus carota*	+	+	.	.	.
*Hyparrhenia hirta*	.	+	.	1	.
*Hypochaeris achyrophorus*	+	+	.	.	.
*Knautia integrifolia* subsp. *integrifolia*	1	+	.	.	.
*Linum usitatissimum* subsp. *angustifolium*	.	+	+	.	.
*Alkanna tinctoria* subsp. *tinctoria*	+	.	.	+	.

**Table A8:** *Plantago holostei-Thymbretum capitatae* Tomaselli & Costanzo ass. nova.

Relevé Number	1	2	3	4	5	6	7	8	9	10	11	12	13	14	15	16
**Typus**								X								
**Surface (m^2^)**	40	40	50	50	40	50	50	60	50	50	60	60	50	60	40	50
**Cover (%)**	60	80	90	85	90	100	80	85	80	75	80	75	80	85	75	80
**Altitude (m)**	40	40	40	50	50	50	50	60	54	50	55	55	50	50	55	55
**Exposure**	E	SE	SE	E	E	SSE	O	O	NO	NO	NO	SE	NE	NE	-	SO
**Slope (°)**	15	15	10	15	15	20	10	15	15	20	20	15	20	10	-	15
**Rockiness**	20	30	10	15	20	20	15	10	10	30	20	10	10	15	10	15
**Stoniness**	40	30	20	20	-	-	30	25	30	30	40	50	60	30	40	30
**Average vegetation height (cm)**	35	40	45	45	50	60	40	50	50	40	40	35	35	40	30	45
**Diagnostic species**																
*Plantago holosteum*	1	2	+	1	1	+	2	1	2	1	2	+	2	1	2	2
*Onobrychis alba* subsp. *alba*	.	.	1	1	+	2	+	1	+	1	1	1	+	2	2	1
*Helianthemum leptophyllum*	1	1	+	.	.	+	.	.	.	+	+	+	1	1	+	+
*Hypericum spruneri*	1	+	.	.	.	.	.	.	.	.	+	.	+	.	.	.
***Cisto eriocephali-Ericion multiflorae***																
*Cistus creticus* subsp. *eriocephalus*	1	2	1	2	1	.	2	1	1	+	1	2	2	1	1	+
*Lotus hirsutus*	+	+	+	.	1	1	+	+	+	+	+	.	+	+	1	+
*Micromeria graeca* subsp. *garganica*	+	1	1	1	1	+	1	1	+	1	1	2	+	.	.	.
*Dianthus tarentinus*	.	.	.	+	.	.	.	.	.	.	.	.	.	.	.	.
***Cisto-Ericetalia manipuliflorae* and *Cisto-Micromerietea***																
*Thymbra capitata*	2	3	5	4	4	5	4	5	5	4	3	2	2	3	2	3
*Satureja cuneifolia*	3	4	2	3	2	2	2	1	1	2	3	4	4	3	4	3
*Fumana thymifolia*	1	2	+	1	2	1	1	1	1	2	2	1	2	2	2	1
*Hippocrepis glauca*	+	1	2	1	2	1	1	2	1	1	1	1	1	1	+	+
*Teucrium capitatum* subsp. *capitatum*	2	1	1	1	1	+	.	.	1	+	+	1	1	1	+	.
*Asyneuma limonifolium* subsp. *limonifolium*	.	1	1	1	.	+	2	1	2	1	2	+	1	.	1	1
*Leontodon apulus*	.	.	.	.	.	+	1	1	1	1	1	+	1	1	1	1
*Cistus salviifolius*	2	1	1	+	2	1	1	.	.	+	+	+	.	.	.	.
*Phlomis fruticosa*	.	.	+	.	.	+	.	+	+	.	.	.	.	.	.	.
*Ononis pusilla*	.	.	.	.	.	.	.	.	.	.	.	.	.	+	.	.
**Other species**																
*Bromopsis erecta* subsp. *erecta*	+	+	1	1	2	.	1	1	+	1	1	1	1	2	2	2
*Anthyllis vulneraria* subsp. *rubriflora*	.	.	+	+	+	1	1	+	+	+	1	+	1	+	1	1
*Bituminaria bituminosa*	.	+	1	1	+	1	1	+	+	.	1	+	1	+	+	+
*Squilla pancration*	+	+	1	1	1	1	.	.	+	+	.	+	+	.	+	+
*Festuca circummediterranea*	.	.	+	1	1	.	1	+	2	1	+	.	1	1	+	.
*Osyris alba*	1	+	.	1	1	.	+	1	+	1	+	+	+	.	.	.
*Reichardia picroides*	+	.	.	.	+	+	+	1	+	.	.	+	+	+	+	+
*Linum tommasinii*	+	1	1	1	1	+	.	+	.	+	.	+	1	.	.	.
*Euphorbia spinosa*	.	+	+	1	1	+	1	+	+	.	.	.	+	+	.	+
*Centaurium erythraea*	+	.	+	.	1	+	.	.	.	+	.	+	.	+	+	1
*Petrorhagia saxifraga* subsp. *saxifraga*	+	1	+	.	.	.	.	+	+	+	+	1	+	.	.	.
*Schoenus nigricans*	+	+	1	+	+	.	.	.	.	.	.	1	+	.	+	.
*Dactylis glomerata* subsp. *hispanica*	.	.	+	+	.	+	+	+	+	.	.	.	.	+	.	.
*Koeleria subcaudata*	.	.	+	1	+	+	.	.	.	.	.	.	.	1	.	.
*Jurinea mollis* subsp. *mollis*	.	.	+	+	.	.	.	.	.	.	.	+	+	.	.	.
*Sixalix atropurpurea* subsp. *maritima*	.	+	.	+	.	.	.	.	.	.	.	.	.	1	.	+
*Carex flacca* subsp. *erythrostachys*	.	.	.	.	.	.	.	.	.	+	+	.	.	1	+	.
*Hyparrhenia hirta*	.	.	.	+	.	.	.	.	.	.	.	1	.	+	.	+
*Brachypodium retusum*	.	.	+	1	+	.	.	.	.	.	.	.	.	.	.	.
*Cynanchica aristata* subsp. *aristata*	.	.	.	.	.	.	.	.	.	.	.	.	.	1	+	+
*Cytisus infestus*	.	.	+	1	1	.	.	.	.	.	.	.	.	.	.	.
*Pinus halepensis* subsp. *halepensis*	.	.	.	+	+	+	.	.	.	.	.	.	.	.	.	.
*Pistacia lentiscus*	.	.	.	.	.	+	.	.	.	.	.	+	+	.	.	.
*Daphne gnidium*	.	.	.	.	.	.	.	+	+	.	.	.	.	.	.	.
*Quercus calliprinos*	.	.	+	.	+	.	.	.	.	.	.	.	.	.	.	.
*Daucus carota* s.l.	.	.	.	.	.	.	.	.	.	.	.	.	.	+	.	.
*Pallenis spinosa* subsp. *spinosa*	.	.	.	.	.	.	.	.	+	.	.	.	.	.	.	.

**Table A9:** Helianthemo jonii-Thymetum capitati Biondi & Guerra 2008.

Relevé Number	1	2	3	4	5	6	7	8	9	10	11	12	13	14	15	16	17	18	19	20	21	22	23	24	25	26	27	28	29	30	31
**Typus**		X																													
**Surface (m^2^)**	50	100	100	150	150	160	30	35	25	20	30	20	12	40	20	20	10	50	30	10	35	100	25	10	25	60	80	40	150	40	40
**Cover (%)**	95	90	100	95	30	20	-	-	-	-	-	80	80	80	90	80	90	80	80	80	90	90	60	50	90	80	90	70	80	70	60
**Altitude (m)**	229	120	227	227	216	216	290	300	280	310	310	190	260	170	150	180	210	154	160	270	210	250	180	220	300	130	130	110	150	114	120
**Exposure**	-	-	-	-	OSO	-	SSE	E	SE	SO	SO	-	E	SE	S	SE	-	SW	SW	-	W	S	W	W	W	SE	-	-	-	-	-
**Slope (°)**	-	-	-	-	5	-	20	350	40	25	10	-	15	20	10	15	-	20	15	-	3	20	10	40	30	5	-	-	-	-	-
**Rockiness**	-	-	-	-	-	-	-	-	-	-	-	35	-	40	20	35	10	15	40	30	-	-	-	-	30	30	10	40	30	-	-
**Stoniness**	-	-	-	-	-	-	-	-	-	-	-	-	-	-	-	-	-	-	-	-	-	-	-	-	-	-	-	-	-	60	60
**Average vegetation height (cm)**	-	-	-	-	-	-	-	-	-	-	-	20	40	30	30	30	30	30	40	30	40	30		20	30	40	30	30	40	35	30
**Diagnostic species**																															
*Helianthemum jonium*	1	1	1	1	1	1	1	1	+	+	+	1	1	+	1	+	+	+	+	+	1	1	1	1	2	+	+	+	+	+	2
*Fumana scoparia*	.	.	.	.	.	.	.	.	.	.	.	+	2	3	4	2	2	3	2	+	4	2	2	2	2	1	3	1	2	4	2
***Cisto eriocephali-Ericion multiflorae***																															
*Phagnalon rupestre* subsp. *illyricum*	.	1	.	1	.	+	+	1	1	1	2	+	.	+	+	+	.	2	.	.	.	+	+	+	.	.	.	.	.	.	.
*Dianthus tarentinus*	.	.	.	.	.	.	+	.	.	.	.	+	.	.	+	+	.	.	+	+	.	.	+	.	.	+	.	+	.	.	.
*Cistus creticus* subsp. *eriocephalus*	.	.	.	.	.	.	.	.	.	.	.	.	1	.	+	.	.	.	.	.	.	.	.	+	1	.	.	.	.	.	.
*Micromeria graeca* subsp. *garganica*	.	+	.	.	.	.	.	.	.	.	.	.	.	.	.	.	.	.	.	.	.	.	.	.	.	.	.	.	.	.	.
***Cisto-Ericetalia manipuliflorae* and *Cisto-Micromerietea***																															
*Thymbra capitata*	5	5	5	5	4	3	4	3	3	3	3	4	3	3	2	3	4	4	4	4	3	3	3	3	4	4	4	4	3	2	3
*Fumana thymifolia*	3	2	2	3	2	2	.	1	.	.	1	.	1	+	2	1	1	1	2	1	+	+	+	2	2	2	+	2	+	.	.
*Hippocrepis glauca*	.	.	.	.	.	.	1	1	1	1	1	+	+	+	+	1	+	+	+	+	+	.	+	+	.	+	1	+	+	.	.
*Fumana ericifolia*	.	.	.	+	.	.	1	2	1	3	3	3	+	+	.	+	+	.	1	1	+	+	+	.	.	+	.	+	+	.	+
*Satureja cuneifolia*	.	.	.	.	2	1	.	1	1	2	.	1	+	2	2	2	.	+	+	+	+	.	3	.	2	+	+	+	1	.	.
*Teucrium capitatum* subsp. *capitatum*	1	+	1	+	.	.	.	.	.	.	+	+	.	.	.	+	+	.	+	.	.	.	.	+	.	+	+	+	.	1	+
*Cistus monspeliensis*	1	+	+	+	.	.	.	.	.	.	1	+	1	.	.	.	.	.	.	.	+	+	.	+	.	+	+	+	1	.	.
*Rosmarinus officinalis*	1	.	+	+	1	1	.	.	.	.	.	.	.	2	.	.	.	.	.	.	2	3	1	.	.	.	.	.	3	+	.
*Linum tommasinii*	.	.	.	.	.	.	.	.	.	.	.	.	.	.	.	+	+	+	+	+	.	.	.	.	.	+	+	+	+	1	+
*Ononis pusilla*	.	.	.	.	.	.	.	.	.	.	.	.	.	+	+	+	.	+	.	+	+	+	+	.	.	+	.	+	+	.	.
*Cistus salviifolius*	.	+	.	.	+	1	.	.	.	.	.	+	.	2	2	+	.	.	1	+	.	.	.	.	.	.	.	.	.	+	.
*Micromeria graeca* subsp. *graeca*	1	1	1	.	.	.	.	.	.	.	.	.	.	.	+	.	.	+	.	.	.	.	+	.	.	.	+	.	.	1	+
*Fumana laevis*	.	.	.	.	.	.	.	.	.	.	.	.	.	1	.	1	.	.	.	.	2	3	2	.	.	2	3	.	2	.	.
*Asyneuma limonifolium* subsp. *limonifolium*	.	+	+	.	.	.	.	.	.	.	.	.	.	.	.	.	.	.	.	.	.	.	.	.	.	+	.	+	+	.	.
*Leontodon apulus*	.	.	.	.	.	.	.	.	.	.	.	.	.	.	+	+	.	.	.	.	.	+	.	.	.	.	.	.	+	.	.
*Hippocrepis comosa*	.	.	.	1	1	1	.	.	.	.	.	.	.	.	.	+	.	.	.	.	.	.	.	.	.	.	.	.	.	.	.
*Helichrysum italicum* subsp. *italicum*	.	.	.	.	.	.	.	.	.	.	.	.	.	.	.	.	.	+	.	.	.	.	.	.	.	.	+	.	.	.	.
**Other species**																															
*Stipa austroitalica* subsp. *austroitalica*	.	+	.	.	.	.	.	1	1	.	1	+	+	.	+	+	.	.	+	.	+	+	+	+	.	+	.	+	+	.	.
*Cynanchica aristata* subsp. *aristata*	.	.	.	.	.	.	.	.	.	.	.	+	.	+	+	.	.	.	+	+	+	+	.	+	.	+	.	+	+	.	.
*Centaurium erythraea*	.	.	.	.	.	.	.	.	.	.	.	+	.	+	.	.	+	.	+	+	+	+	.	.	.	+	+	.	+	.	.
*Petrosedum ochroleucum* subsp. *mediterraneum*	.	.	.	.	.	.	.	.	.	.	+	.	+	.	+	+	.	.	.	.	+	+	.	+	.	+	.	.	.	.	+
*Linum strictum*	+	.	.	.	1	1	.	.	.	.	.	.	.	.	.	.	.	.	.	.	.	.	.	+	+	.	+	+	.	.	.
*Ononis reclinata*	.	.	.	+	.	.	.	.	.	.	+	.	.	.	.	+	.	.	.	.	.	+	+	.	.	+	+	.	.	.	.
*Pinus halepensis* subsp. *halepensis*	.	.	.	+	.	.	.	.	.	.	.	.	.	+	.	+	.	.	.	+	+	.	+	.	.	.	.	.	.	.	+
*Odontites luteus* subsp. *luteus*	+	.	.	.	+	.	.	.	.	.	.	.	.	.	.	.	.	.	.	.	+	+	.	.	.	.	.	.	.	+	1
*Thesium humifusum*	.	.	.	.	.	.	+	.	.	.	.	+	.	.	.	.	.	+	+	+	.	.	+	.	.	.	.	.	.	.	.
*Centaurea subtilis*	.	.	.	.	.	.	.	.	.	.	.	1	.	.	.	+	.	.	1	1	.	.	.	.	.	+	.	.	.	.	.
*Crupina crupinastrum*	.	+	.	.	.	.	.	+	.	.	.	.	+	.	.	.	.	.	.	.	.	.	+	+	.	.	.	.	.	.	.
*Allium sphaerocephalon*	.	.	.	.	.	.	.	.	.	.	.	.	.	.	.	.	.	.	+	.	+	.	.	+	.	+	.	.	.	.	.
*Anthyllis vulneraria* subsp. *maura*	+	+	1	+	.	.	.	.	.	.	.	.	.	.	.	.	.	.	.	.	.	.	.	.	.	.	.	.	.	.	.
*Carex flacca* subsp. *erythrostachys*	.	.	.	.	.	.	.	.	.	.	.	.	.	.	.	.	.	.	.	.	.	+	.	+	+	+	.	.	.	.	.
*Festuca circummediterranea*	.	.	.	.	.	.	.	.	.	.	.	.	.	.	.	+	.	.	.	.	.	.	.	.	.	+	.	+	+	.	.
*Hypochaeris achyrophorus*	+	.	.	.	.	.	.	.	.	.	+	.	+	.	.	.	.	.	.	.	+	.	.	.	.	.	.	.	.	.	.
*Jurinea mollis* subsp. *mollis*	.	.	.	.	.	.	.	.	.	.	.	+	.	.	.	.	.	+	+	+	.	.	.	.	.	.	.	.	.	.	.
*Lagurus ovatus*	+	.	.	.	.	.	.	.	.	.	.	.	.	.	.	.	.	.	.	.	.	+	+	.	.	+	.	.	.	.	.
*Onosma echioides* subsp. *echioides*	.	.	.	.	.	.	.	.	.	.	+	.	.	.	.	+	.	.	+	.	.	.	.	.	.	.	.	.	.	+	.
*Pistacia lentiscus*	.	.	.	.	.	.	1	+	.	.	.	+	.	.	.	.	.	.	.	.	.	.	.	+	.	.	.	.	.	.	.
*Poa bulbosa* subsp. *bulbosa*	.	.	.	+	+	.	.	.	.	.	+	.	.	.	.	.	.	.	.	.	+	.	.	.	.	.	.	.	.	.	.
*Thesium humile*	.	.	.	.	.	.	+	+	1	1	.	.	.	.	.	.	.	.	.	.	.	.	.	.	.	.	.	.	.	.	.
*Anthyllis vulneraria* subsp. *rubriflora*	.	.	.	.	.	.	.	.	.	.	.	.	.	.	+	.	.	.	.	.	.	.	.	.	.	.	.	.	+	.	+
*Convolvulus cantabrica*	.	.	.	.	.	.	.	.	.	.	+	.	+	.	.	.	.	+	.	.	.	.	.	.	.	.	.	.	.	.	.
*Cytisus spinescens*	.	.	.	.	.	.	.	.	.	.	.	.	.	+	+	+	.	.	.	.	.	.	.	.	.	.	.	.	.	.	.
*Petrosedum rupestre*	.	.	.	.	+	1	1	.	.	.	.	.	.	.	.	.	.	.	.	.	.	.	.	.	.	.	.	.	.	.	.
*Reichardia picroides*	.	.	+	.	.	.	.	.	.	.	.	.	.	.	.	.	.	+	.	.	.	.	.	.	.	+	.	.	.	.	.
*Reseda alba* subsp. *alba*	.	.	.	.	.	.	.	1	+	+	.	.	.	.	.	.	.	.	.	.	.	.	.	.	.	.	.	.	.	.	.
*Rhamnus saxatilis*	.	.	.	.	.	.	2	.	+	.	.	.	.	.	.	.	.	.	.	.	.	.	.	+	.	.	.	.	.	.	.
*Sixalix atropurpurea* subsp. *maritima*	.	.	.	.	.	.	.	.	.	.	.	.	.	.	.	.	.	+	.	.	.	.	.	.	.	.	.	.	.	+	+
*Alkanna tinctoria* subsp. *tinctoria*	.	.	.	.	.	.	.	.	.	.	.	.	.	.	+	.	.	.	.	.	.	+	.	.	.	.	.	.	.	.	.
*Anthyllis vulneraria* subsp. *weldeniana*	.	.	.	.	.	.	1	.	.	+	.	.	.	.	.	.	.	.	.	.	.	.	.	.	.	.	.	.	.	.	.
*Asphodelus ramosus* subsp. *ramosus*	.	.	.	+	.	.	.	.	.	.	+	.	.	.	.	.	.	.	.	.	.	.	.	.	.	.	.	.	.	.	.
*Brachypodium distachyon*	.	.	.	.	.	+	.	.	.	.	.	.	.	.	.	.	.	.	.	.	.	.	.	+	.	.	.	.	.	.	.
*Squilla pancration*	.	.	.	.	+	.	+	.	.	.	.	.	.	.	.	.	.	.	.	.	.	.	.	.	.	.	.	.	.	.	.
*Daucus carota* s.l.	.	+	.	.	.	.	.	.	.	.	.	.	.	.	.	.	.	+	.	.	.	.	.	.	.	.	.	.	.	.	.
*Lysimachia arvensis* subsp. *arvensis*	.	.	.	.	+	+	.	.	.	.	.	.	.	.	.	.	.	.	.	.	.	.	.	.	.	.	.	.	.	.	.

**Table A10:** Phagnalo-Fumanetum thymifoliae Biondi 2000.

Relevé Number	1	2	3	4	5	6	3	4	5	6	7	8	9	10
**Typus**		X												
**Surface (m^2^)**	8	8	8	20	15	20	140	80	40	50	10	10	25	30
**Cover (%)**	60	90	60	80	100	80	75	95	100	70	60	60	70	70
**Altitude (m)**	100	20	100	10	70	30	325	340	339	342	340	335	200	200
**Exposure**	SSW	W	SSW	S	SE	S	SSE	S	NE	O	SO	SO	SSO	SSO
**Slope (°)**	10	20	10	40	20	60	15	10	30	10	30	30	10	15
**Rockiness**	-	-	-	-	-	-	-	-	-	-	10	10	-	-
**Stoniness**	-	-	-	-	-	-	-	-	-	-	80	80	80	70
**Average vegetation height (cm)**	-	-	-	-	-	-	-	-	-	-	20	20	30	30
**Diagnostic species**														
*Phagnalon rupestre* subsp. *illyricum*	1	2	1	1	.	3	4	4	5	4	3	3	4	3
***Cisto eriocephali-Ericion multiflorae***														
*Cistus creticus* subsp. *eriocephalus*	.	.	.	.	.	.	.	1	+	+	.	.	.	.
***Cisto-Ericetalia manipuliflorae* and *Cisto-Micromerietea***														
*Fumana thymifolia*	2	3	3	2	2	3	3	2	3	3	3	2	3	2
*Micromeria graeca* subsp. *graeca*	+	1	1	+	1	2	+	1	+	1	.	.	1	1
*Teucrium capitatum* subsp. *capitatum*	.	.	.	+	1	.	1	.	.	.	1	1	1	+
*Thymbra capitata*	.	.	.	.	3	.	.	1	1	+	.	.	1	+
*Hippocrepis comosa*	.	.	.	.	.	.	1	1	.	.	.	.	.	+
*Helianthemum jonium*	.	.	.	.	.	.	.	.	.	+	.	.	1	2
*Fumana ericifolia*	.	.	.	2	.	.	.	.	.	.	.	.	+	1
*Rosmarinus officinalis*	.	.	.	.	+	+	.	.	.	.	.	.	.	.
*Cistus creticus* subsp. *creticus*	.	.	.	.	.	.	1	.	.	.	.	.	.	.
*Cistus monspeliensis*	.	.	.	.	.	.	.	.	+	.	.	.	.	.
	.	.	.	.	.	.								
**Other species**	.	.	.	.	.	.								
*Hyparrhenia hirta*	2	1	.	+	.	.	+	.	.	.	1	1	.	.
*Linum strictum*	.	.	.	.	.	.	+	.	.	.	1	+	+	+
*Thapsia asclepium*	.	.	.	.	.	.	1	+	+	.	2	1	.	.
*Convolvulus altheoides*	.	.	.	.	.	.	.	.	.	.	+	1	+	+
*Reichardia picroides*	.	.	.	.	.	.	.	+	+	.	.	.	+	+
*Cynanchica aristata* subsp. *aristata*	.	.	.	.	1	+	.	.	.	1	.	.	1	.
*Petrosedum sediforme*	+	1	1	.	.	.	1	.	.	.	.	.	.	.
*Squilla pancration*	.	.	1	.	1	.	1	.	.	.	.	.	.	+
*Sixalix atropurpurea* subsp. *maritima*	.	.	.	.	.	.	+	.	.	.	.	.	+	+
*Convolvulus elegantissimus*	.	.	.	.	.	.	+	.	+	.	.	.	.	.
*Cytisus spinescens*	.	.	.	.	.	.	1	.	.	1	.	.	.	.
*Asphodeline liburnica*	.	.	.	.	.	.	.	+	.	+	.	.	.	.
*Brachypodium distachyon*	.	.	.	.	.	.	.	+	.	+	.	.	.	.
*Centaurea brulla*	.	.	.	.	.	.	.	1	.	+	.	.	.	.
*Onosma echioides* subsp. *echioides*	.	.	.	.	.	.	.	.	.	.	.	.	+	+
*Pallenis spinosa* subsp. *spinosa*	.	.	.	.	.	.	.	+	.	.	.	.	+	.
*Scorzonera villosa* subsp. *columnae*	.	.	.	.	.	.	+	+	.	.	.	.	.	.
*Stipa austroitalica* subsp. *austroitalica*	.	.	.	.	.	.	.	+	.	+	.	.	.	.
*Thymus spinulosus*	.	.	.	.	.	.	1	.	.	+	.	.	.	.
*Helianthemum canum*	.	.	.	1	+	.	.	.	.	.	.	.	.	.
*Euphorbia dendroides*	+	.	+	.	.	.	.	.	.	.	.	.	.	.
*Stipa bromoides*	.	.	.	+	1	.	.	.	.	.	.	.	.	.
*Pinus halepensis* subsp. *halepensis*	.	.	.	+	.	+	.	.	.	.	.	.	.	.

**Table A11:** *Sileno otitis-Helianthemetum lippii* Tomaselli & Costanzo ass. nova.

Relevé Number	1	2	3	4	5	6	7	8	9	10	11	12
**Typus**									X			
**Surface (m^2^)**	30	30	40	30	40	30	15	20	20	20	20	15
**Cover (%)**	65	70	65	50	65	60	60	60	50	70	65	60
**Altitude (m)**	4	3	4	4	3	3	8	10	8	4	9	10
**Average vegetation height (cm)**	20	30	20	25	30	25	20	30	20	30	25	25
**Diagnostic species**												
*Helianthemum lippii*	2	2	3	4	4	3	2	3	3	4	3	3
*Silene otites*	.	1	+	.	+	1	+	.	+	1	1	+
***Cisto eriocephali-Ericion multiflorae***												
*Cistus creticus* subsp. *eriocephalus*	2	2	3	1	2	1	2	1	1	1	1	+
*Lotus hirsutus*	.	.	.	.	.	.	+	+	+	.	.	.
***Cisto-Ericetalia manipuliflorae* and *Cisto-Micromerietea***												
*Rosmarinus officinalis*	1	+	.	.	.	.	+	1	.	.	.	.
*Helianthemum jonium*	.	.	.	.	.	.	2	+	+	.	+	.
*Fumana thymifolia*	.	.	.	.	.	.	.	.	1	.	+	.
*Cistus salviifolius*	+	.	.	.	.	.	.	.	.	.	.	.
**Other species**												
*Festuca fasciculata*	+	+	+	+	1	+	1	1	+	1	+	1
*Silene colorata*	+	+	+	.	+	+	1	+	+	1	1	+
*Sixalix atropurpurea* subsp. *maritima*	+	1	+	1	1	2	.	.	1	2	1	1
*Lotus cytisoides*	.	1	+	.	+	.	+	1	+	1	1	+
*Smilax aspera*	+	+	+	1	.	.	+	+	.	+	.	+
*Pancratium maritimum*	+	1	.	+	1	+	+	.	.	.	.	+
*Juniperus macrocarpa*	.	+	+	+	+	+	.	+	.	.	.	+
*Asparagus acutifolius*	+	+	+	1	.	.	.	+	+	.	.	.
*Ononis variegata*	.	.	+	.	.	+	+	1	.	1	.	+
*Pinus halepensis* subsp. *halepensis*	+	+	+	+	+	.	.	.	.	+	.	.
*Hedypnois rhagadioloides*	+	+	+	.	+	.	.	.	.	.	.	.
*Rubia peregrina*	+	1	+	+	.	.	.	.	.	.	.	.
*Matthiola sinuata*	.	1	+	+	.	.	.	.	.	.	.	.
*Alkanna tinctoria* subsp. *tinctoria*	.	.	.	.	.	.	.	+	+	.	.	+
*Lonicera implexa* subsp. *implexa*	+	.	.	+	.	.	.	.	.	.	.	.

**Table A12:** *Ruto chalepensis-Salvietum trilobae* Biondi & Guerra 2008.

Relevé Number	1	2	3	4	5
**Typus**					X
**Surface (m^2^)**	80	100	400	100	100
**Cover (%)**	90	100	100	90	90
**Altitude (m)**	150	196	198	235	200
**Exposure**	SE	-	-	-	E
**Slope (°)**	40	-	-	-	30
**Diagnostic species**					
*Salvia fruticosa*	4	4	5	5	5
*Ruta chalepensis*	3	3	2	2	3
*Aurinia saxatilis* subsp. *megalocarpa*	+	+	.	.	.
*Coronilla valentina*	.	+	1	1	.
***Cisto eriocephali-Ericion multiflorae***					
*Phagnalon rupestre* subsp. *illyricum*	1	1	1	+	+
***Cisto-Ericetalia manipuliflorae* and *Cisto-Micromerietea***					
*Micromeria graeca* subsp. *graeca*	1	+	.	1	+
*Cistus creticus* subsp. *creticus*	1	.	.	.	1
*Rosmarinus officinalis*	.	.	.	+	.
**Other species**					
*Olea europaea*	2	2	2	2	+
*Squilla pancration*	+	.	2	1	1
*Ferula communis*	.	.	1	+	+
*Trachynia distachya*	+	+	+	.	.
*Pistacia lentiscus*	+	+	.	.	2
*Stachys major*	1	1	.	1	.
*Oloptum miliaceum*	+	.	.	.	1
*Dasypyrum villosum*	.	+	.	.	+
*Muscari comosum*	+	.	1	.	.
*Petrorhagia saxifraga* subsp. *saxifraga*	+	.	.	+	.
*Phillyrea latifolia*	+	.	.	+	.
*Pinus halepensis* subsp. *halepensis*	.	.	1	+	.
*Allium subhirsutum* subsp. *subhirsutum*	.	.	.	.	+
*Rhamnus alaternus* subsp. *alaternus*	.	.	.	.	+
*Asparagus acutifolius*	.	.	.	.	+
*Asphodeline lutea*	+	.	.	.	.
*Asphodelus ramosus* subsp. *ramosus*	+	.	.	.	.
*Convolvulus cantabrica*	.	.	.	+	.
*Eryngium campestre*	.	.	.	+	.
*Juniperus oxycedrus*	.	1	.	.	.
*Lagurus ovatus*	.	.	.	.	+
*Petrosedum rupestre*	+	.	.	.	.
*Pistacia terebinthus* subsp. *terebinthus*	.	.	.	.	+
*Scorzonera villosa* subsp. *columnae*	+	.	.	.	.
*Stipa austroitalica* subsp. *austroitalica*	.	.	.	+	.
*Stipellula capensis*	.	+	.	.	.
*Bituminaria bituminosa*	.	.	.	.	1

**Table A13:** *Chamaecytiso spinescentis-Cistetum eriocephali* Biondi & Guerra 2008.

Relevé Number	1	2	3	4	5	6
**Typus**				X		
**Surface (m^2^)**	100	200	80	80	20	50
**Cover (%)**	100	90	100	100	80	90
**Altitude (m)**	300	335	290	340	310	300
**Exposure**	NNE	-	N	OSO	OSO	OSO
**Slope (°)**	5	-	10	10	5	3
**Diagnostic species**						
*Cytisus spinescens*	+	3	4	1	1	2
***Cisto eriocephali-Ericion multiflorae***						
*Cistus creticus* subsp. *eriocephalus*	5	1	3	5	4	4
*Lotus hirsutus*	.	2	+	+	.	.
***Cisto-Ericetalia manipuliflorae* and *Cisto-Micromerietea***						
*Micromeria graeca* subsp. *graeca*	.	.	+	1	+	+
*Cistus monspeliensis*	.	.	.	1	1	2
*Teucrium capitatum* subsp. *capitatum*	.	.	.	+	+	1
*Helianthemum jonium*	+	.	.	.	+	.
*Cistus creticus* subsp. *creticus*	.	1	.	.	.	.
*Hippocrepis glauca*	.	+	.	.	.	.
*Fumana thymifolia*	.	.	.	1	.	.
*Thymbra capitata*	.	.	.	+	.	.
**Other species**						
*Pistacia lentiscus*	1	.	+	1	+	+
*Thapsia asclepium*	1	+	.	+	.	.
*Helictotrichon convolutum*	.	.	.	+	1	+
*Dactylis glomerata* subsp. *hispanica*	.	.	+	.	+	.
*Linum strictum*	.	.	.	.	+	+
*Gastridium ventricosum*	.	.	.	.	+	+
*Asparagus acutifolius*	.	+	.	.	.	.
*Rhamnus saxatilis*	.	1	.	.	.	.
*Quercus trojana* subsp. *trojana*	.	.	+	.	.	.
*Asphodelus ramosus* subsp. *ramosus*	.	1	.	.	.	.
*Macrobriza maxima*	.	.	.	.	+	.
*Allium subhirsutum* subsp. *subhirsutum*	.	+	.	.	.	.
*Ferula communis*	.	1	.	.	.	.
*Osyris alba*	.	.	1	.	.	.
*Convolvulus cantabrica*	.	.	.	+	.	.
*Pistacia terebinthus* subsp. *terebinthus*	.	.	.	.	+	.
*Stachys major*	+	.	.	.	.	.
*Clematis flammula*	.	.	1	.	.	.
*Rubia peregrina*	.	.	+	.	.	.
*Helichrysum italicum* subsp. *italicum*	.	.	+	.	.	.
*Poterium sanguisorba*	+	.	.	.	.	.
*Bromopsis erecta* subsp. *erecta*	.	.	.	.	+	.
*Centaurea subtilis*	1	.	.	.	.	.
*Rhamnus alaternus* subsp. *alaternus*	.	.	.	.	1	.
*Anthyllis vulneraria* subsp. *rubriflora*	.	.	.	.	+	.
*Cynanchica aristata* subsp. *aristata*	.	.	.	+	.	.
*Centaurea brulla*	.	.	.	+	.	.
*Centaurium erythraea*	.	.	+	.	.	.
*Cytisus infestus*	1	.	.	.	.	.
*Daucus carota* s.l.	.	.	+	.	.	.
*Jurinea mollis* subsp. *mollis*	.	.	.	+	.	.
*Linum trigynum*	.	.	.	.	+	.
*Petrosedum rupestre*	.	.	.	.	+	.
*Reichardia picroides*	.	.	+	.	.	.

**Table A14:** *Erico multiflorae-Halimietum halimifolii* Taffetani & Biondi 1992.

Relevé Number	1	2	3	4	5	6	7	8	9	10	11	12	13	14	15	16	17	18
**Typus**					X													
**Surface (m^2^)**	20	30	20	20	40	40	150	100	150	20	150	150	120	100	100	50	100	100
**Cover (%)**	90	95	100	100	100	100	90	100	95	50	95	90	80	90	90	90	90	90
**Altitude (m)**	4	3	4	3	3	3	4	3	4	4	5	4	9	3	4	3	3	3
**Exposure**	-	-	-	-	-	-	S	SE	-	-	-	-	-	-	-	-	-	-
**Slope (°)**	-	-	-	-	-	-	10	10	-	-	-	-	-	-	-	-	-	-
**Average vegetation height (cm)**	120	130	140	120	130	150	120	140	120	120	150	120	120	-	-	-	-	-
**Diagnostic species**																		
*Halimium halimifolium* subsp. *halimifolium*	4	4	4	1	2	3	4	4	5	3	5	4	4	2	2	2	3	4
***Cisto eriocephali-Ericion multiflorae***																		
*Erica multiflora*	1	1	1	2	2	2	1	+	1	1	2	1	2	2	3	3	3	2
*Cistus creticus* subsp. *eriocephalus*	.	.	.	2	2	2	1	1	+	+	+	+	1	3	3	2	2	2
*Lotus hirsutus*	.	.	.	.	.	.	1	.	1	.	.	.	.	1	+	+	.	.
***Cisto-Ericetalia manipuliflorae* and *Cisto-Micromerietea***																		
*Rosmarinus officinalis*	3	3	3	4	4	3	2	3	2	3	2	2	3	3	1	+	1	1
*Cistus salviifolius*	2	1	2	.	.	.	2	1	1	2	2	1	1	2	2	1	1	1
*Fumana thymifolia*	.	.	.	.	.	.	1	+	+	1	+	1	1	3	2	2	2	2
*Helianthemum jonium*	.	.	.	+	.	.	+	.	+	+	+	+	+	2	1	2	1	2
*Teucrium capitatum* subsp. *capitatum*	.	+	.	.	.	.	.	.	+	1	+	+	1	2	1	2	1	1
*Micromeria graeca* subsp. *graeca*	+	+	.	.	.	.	.	.	.	+	.	.	.	1	+	1	1	+
**Other species**																		
*Phillyrea latifolia*	+	+	+	1	+	+	.	+	+	1	+	+	.	1	+	+	+	1
*Pistacia lentiscus*	+	+	+	1	+	.	+	1	.	+	+	+	+	1	1	1	1	1
*Asparagus acutifolius*	+	+	1	.	+	+	+	+	+	1	+	+	1	.	.	+	+	+
*Petrorhagia saxifraga* subsp. *saxifraga*	.	.	.	.	.	.	+	+	.	1	+	+	.	2	1	2	1	2
*Osyris alba*	.	.	.	.	+	+	+	.	+	.	.	.	.	3	1	+	1	1
*Silene vulgaris* subsp. *tenoreana*	+	+	.	.	.	+	+	.	+	1	+	.	+	.	.	.	.	.
*Lonicera implexa* subsp. *implexa*	.	.	.	.	.	.	1	1	+	.	.	.	+	1	.	.	+	+
*Lotus creticus*	.	.	.	.	.	.	+	+	1	+	.	+	+	.	.	.	.	.
*Smilax aspera*	.	.	.	.	.	.	.	+	+	.	.	.	.	1	+	.	+	+
*Silene colorata*	.	.	.	.	.	.	+	.	+	+	.	+	+	.	.	.	.	.
*Arbutus unedo*	.	.	.	.	.	.	.	+	.	+	+	.	+	.	+	.	.	.
*Rhamnus alaternus* subsp. *alaternus*	.	.	+	1	.	.	.	.	.	.	.	.	+	.	.	+	+	.
*Pinus halepensis* subsp. *halepensis*	.	.	.	.	.	.	.	+	+	+	1	.	.	.	.	.	.	.
*Emerus major* subsp. *emeroides*	.	.	.	.	.	.	+	+	.	.	.	.	+	.	.	.	.	.
*Odontites luteus* subsp. *luteus*	1	+	1	.	.	.	.	.	.	.	.	.	.	.	.	.	.	.
*Linum tenuifolium*	1	+	.	+	.	.	.	.	.	.	.	.	.	.	.	.	.	.
*Dactylis glomerata* subsp. *hispanica*	.	.	.	.	.	.	.	.	.	.	.	.	.	.	.	+	+	+
*Crepis vesicaria*	.	.	.	.	.	.	.	.	.	.	+	+	.	.	.	.	.	.
*Silene italica* subsp. *italica*	.	.	.	.	.	.	.	.	.	+	+	.	.	.	.	.	.	.
*Teucrium flavum* subsp. *flavum*	.	.	.	.	.	.	.	.	.	.	.	.	.	+	+	.	.	.
*Sulla capitata*	.	.	.	.	.	.	.	.	.	.	.	.	1	.	.	.	.	.
*Juniperus macrocarpa*	.	.	+	.	.	.	.	.	.	.	.	.	.	.	.	.	.	.
*Sonchus bulbosus* subsp. *bulbosus*	.	.	.	.	.	.	.	.	.	.	.	.	1	.	.	.	.	.
*Quercus ilex* subsp. *ilex*	.	.	.	.	.	.	.	.	.	.	.	.	.	.	+	.	.	.
*Sixalix atropurpurea* subsp. *maritima*	+	.	.	.	.	.	.	.	.	.	.	.	.	.	.	.	.	.

**Table A15:** *Helianthemo jonii-Fumanetum thymifoliae* Taffetani & Biondi 1992.

Relevé Number	1	2	3	4	5	6	7	8	9	10	11	12	13	14	15	16	17	18	19
**Typus**		X																	
**Surface (m^2^)**	10	12	10	10	12	20	15	20	30	25	30	20	30	20	40	20	30	20	30
**Cover (%)**	80	50	80	70	70	100	80	50	60	50	60	50	60	50	65	40	80	60	70
**Altitude (m)**	-	-	-	-	-	-	-	3	3	3	4	4	6	4	8	7	8	8	10
**Average vegetation height (cm)**	-	-	-	-	-	-	-	-	30	20	20	25	25	20	25	25	20	25	20
**Diagnostic species**																			
*Helianthemum jonium*	+	+	+	1	2	1	.	2	2	1	1	2	2	3	1	+	1	1	+
*Lotus creticus*	+	+	1	1	1	.	+	+	1	+	+	.	1	.	.	.	.	.	.
*Verbascum niveum* subsp. *garganicum*	.	+	.	+	+	+	+	.	.	.	.	.	.	.	.	.	.	.	.
***Cisto eriocephali-Ericion multiflorae***																			
*Cistus creticus* subsp. *eriocephalus*	.	.	+	+	.	.	.	.	.	1	+	.	+	.	+	1	1	+	2
*Erica multiflora*	.	.	.	.	.	.	.	+	+	+	.	+	+	.	.	.	.	.	.
*Lotus hirsutus*	.	.	.	.	.	.	.	.	.	.	.	.	.	.	.	.	.	.	+
***Cisto-Ericetalia manipuliflorae* and *Cisto-Micromerietea***																			
*Fumana thymifolia*	4	4	3	3	3	2	3	3	3	3	3	2	3	2	4	4	4	3	3
*Teucrium capitatum* subsp. *capitatum*	1	1	+	1	2	2	2	1	1	2	1	1	2	.	2	1	2	1	2
*Rosmarinus officinalis*	+	.	+	+	.	.	.	1	1	+	1	1	+	+	+	+	1	1	1
*Cistus salviifolius*	+	+	.	.	.	+	+	+	1	.	2	1	1	1	.	.	.	.	.
*Halimium halimifolium* subsp. *halimifolium*	.	.	.	+	+	+	.	.	+	.	.	+	.	.	.	.	.	.	.
*Micromeria graeca* subsp. *graeca*	.	.	.	.	.	+	.	.	.	.	.	.	.	.	+	+	.	.	.
**Other species**																			
*Petrorhagia saxifraga* subsp. *saxifraga*	.	.	.	.	.	.	.	+	.	+	+	+	1	.	1	+	+	1	1
*Sixalix atropurpurea* subsp. *maritima*	.	+	1	1	+	.	1	.	.	+	.	+	.	.	+	.	.	.	.
*Asparagus acutifolius*	+	.	.	.	.	+	+	.	.	.	+	.	.	.	+	+	.	+	+
*Silene colorata*	.	.	.	.	.	.	.	1	1	1	+	1	+	1	.	.	.	.	.
*Medicago littoralis*	.	.	.	.	.	.	.	+	+	+	1	1	+	.	.	.	.	.	.
*Sonchus bulbosus* subsp. *bulbosus*	.	.	.	.	.	.	.	.	+	+	1	+	+	+	.	.	.	.	.
*Plantago albicans*	.	.	.	.	.	.	.	.	.	.	.	.	.	.	+	2	+	1	+
*Coronilla scorpioides*	.	.	.	.	.	.	.	.	+	1	1	1	1	.	.	.	.	.	.
*Sulla capitata*	.	.	.	.	.	.	.	.	1	2	1	2	1	.	.	.	.	.	.
*Linum tenuifolium*	+	.	.	+	1	1	.	.	.	.	.	.	.	.	.	.	.	.	.
*Herniaria hirsuta* subsp. *hirsuta*	.	.	.	.	.	.	.	.	.	.	.	.	.	.	+	+	+	1	.
*Linum trigynum*	.	.	.	.	.	.	.	+	.	+	+	.	+	.	.	.	.	.	.
*Alkanna tinctoria* subsp. *tinctoria*	.	.	.	.	.	.	.	.	1	1	.	+	.	.	.	.	.	.	.
*Bellardia trixago*	.	.	.	.	.	.	.	.	.	.	.	.	.	.	.	.	2	1	1
*Poa bulbosa* subsp. *bulbosa*	.	.	.	.	.	.	.	.	.	.	.	.	.	.	.	.	1	+	+
*Silene vulgaris* subsp. *tenoreana*	.	.	.	.	1	.	.	.	.	.	.	.	.	+	.	.	.	.	.
*Silene otites*	.	.	.	.	.	.	.	.	.	.	.	.	.	.	.	+	+	.	.
*Odontites luteus* subsp. *luteus*	.	.	.	.	.	1	1	.	.	.	.	.	.	.	.	.	.	.	.
*Osyris alba*	.	.	.	.	+	.	.	.	.	.	+	.	.	.	.	.	.	.	.
*Pinus halepensis* subsp. *halepensis*	.	.	.	.	.	.	.	.	.	+	.	.	+	.	.	.	.	.	.
*Thapsia asclepium*	.	.	.	.	.	.	.	.	.	.	.	.	.	.	+	.	+	.	.
*Thinopyrum acutum*	.	+	.	.	.	.	.	.	.	.	.	.	.	.	.	.	.	.	.
*Allium sphaerocephalon*	.	.	.	.	.	+	.	.	.	.	.	.	.	.	.	.	.	.	.
*Juniperus macrocarpa*	+	.	.	.	.	.	.	.	.	.	.	.	.	.	.	.	.	.	.
*Pistacia lentiscus*	.	.	.	.	.	.	.	+	.	.	.	.	.	.	.	.	.	.	.
*Petrosedum ochroleucum* subsp. *mediterraneum*	.	.	.	.	.	.	.	.	.	.	.	.	.	.	.	.	.	.	+

**Table A16:** *Cistetum salvifolio-clusii* Bartolo, Giardina, Minissale & Spampinato 1987.

Relevé Number	1	2	3	4	5	6	7
**Typus**							
**Surface (m^2^)**	200	150	20	50	50	150	100
**Cover (%)**	85	80	70	70	80	80	90
**Altitude (m)**	-	-	-	-	-	-	-
**Diagnostic species**							
*Cistus clusii*	1	2	+	1	1	1	1
***Cisto eriocephali-Ericion multiflorae***							
*Cistus creticus* subsp. *eriocephalus*	1	1	2	1	1	1	1
*Erica multiflora*	+	1	1	1	1	1	+
***Cisto-Ericetalia manipuliflorae* and *Cisto-Micromerietea***							
*Rosmarinus officinalis*	1	2	2	3	2	2	2
*Cistus salviifolius*	1	1	+	1	1	1	+
*Fumana thymifolia*	+	+	+	1	+	+	+
*Helianthemum jonium*	+	+	+	+	+	+	+
*Halimium halimifolium* subsp. *halimifolium*	+	+	+	+	+	.	.
*Teucrium capitatum* subsp. *capitatum*	+	+	.	+	+	+	.
**Other species**							
*Phillyrea latifolia*	+	+	1	+	+	+	+
*Sulla capitata*	1	1	+	+	1	1	+
*Pistacia lentiscus*	+	.	+	+	+	+	+
*Juniperus macrocarpa*	+	+	.	+	+	+	+
*Anthyllis vulneraria* subsp. *maura*	+	+	+	+	+	+	.
*Asparagus acutifolius*	+	+	+	+	+	.	+
*Osyris alba*	+	.	+	+	+	+	.
*Crepis vesicaria*	.	+	.	+	+	+	+
*Sonchus bulbosus* subsp. *bulbosus*	+	+	1	.	.	+	+
*Poa bulbosa* subsp. *bulbosa*	+	+	.	+	+	+	.
*Quercus ilex* subsp. *ilex*	+	+	+	+	+	.	.
*Lagurus ovatus*	+	.	+	+	+	.	+
*Petrorhagia saxifraga* subsp. *saxifraga*	+	+	.	+	.	+	.
*Arbutus unedo*	.	+	.	+	.	+	+
*Sixalix atropurpurea* subsp. *maritima*	+	+	+	+	.	.	.
*Carex distachya*	+	+	.	.	+	+	.
*Lonicera implexa* subsp. *implexa*	+	.	+	.	.	.	+
*Silene colorata*	+	.	+	.	.	+	.
*Medicago minima*	+	.	.	+	.	+	.
*Alkanna tinctoria* subsp. *tinctoria*	+	+	.	.	.	+	.
*Silene vulgaris* subsp. *tenoreana*	+	.	+	.	.	.	.
*Silene italica* subsp. *italica*	.	.	.	+	.	+	.
*Linum strictum*	.	.	.	.	.	+	+
*Reichardia picroides*	.	.	.	+	+	.	.
*Lotus creticus*	.	.	+	.	.	.	.
*Emerus major* subsp. *emeroides*	.	.	.	.	+	.	.
*Lotus hirsutus*	.	.	+	.	.	.	.
*Medicago littoralis*	.	.	.	.	.	.	+
*Odontites luteus* subsp. *luteus*	.	.	.	.	.	+	.
*Macrobriza maxima*	.	.	.	.	.	.	+
*Clematis flammula*	.	+	.	.	.	.	.
*Dactylis glomerata* subsp. *hispanica*	.	.	+	.	.	.	.
*Rubia peregrina*	.	.	+	.	.	.	.
*Urospermum dalechampii*	+	.	.	.	.	.	.
*Dasypyrum villosum*	.	.	.	.	.	+	.
*Hypochaeris achyrophorus*	.	.	.	.	.	.	+

**Table A17:** *Centaureo subtilis-Thymetum capitati* Terzi & D’Amico 2006.

Relevé Number	1	2	3	4	5	6	7	8	9	10	11	12	13	14	15	16	17	18	19
**Typus**	X																		
**Surface (m^2^)**	30	30	30	30	30	30	30	30	30	30	30	30	50	80	50	50	80	80	50
**Cover (%)**	80	70	60	60	40	60	50	70	70	80	80	80	70	70	60	60	70	80	80
**Altitude (m)**	410	370	370	420	360	410	360	360	415	400	370	420	358	365	385	418	415	415	410
**Exposure**	0	350	320	5	340	310	35	30	10	330	20	350	-	-	-	-	-	-	-
**Slope (°)**	5	10	5	12	5	30	10	10	15	30	30	25	-	-	-	-	-	-	-
**Rockiness**	5	30	30	20	45	40	30	15	20	10	20	10	-	-	-	-	-	-	-
**Stoniness**	5	20	15	10	5	30	40	50	30	30	40	50	-	-	-	-	-	-	-
**Diagnostic species**																			
*Fumana procumbens*	2	1	+	1	1	2	2	1	2	2	2	1	1	1	1	2	1	2	2
*Lomelosia crenata* subsp. *crenata*	+	2	1	3	.	2	+	2	.	.	+	1	+	+	1	+	1	2	.
*Matthiola fruticulosa* subsp. *fruticulosa*	.	+	+	.	+	.	.	.	.	.	.	.	+	2	2	2	3	+	2
*Scabiosa holosericea*	.	.	.	.	.	.	+	+	+	+	+	+	.	.	.	.	.	.	.
*Anthemis hydruntina* subsp. *hydruntina*	.	.	.	.	.	.	.	.	.	.	.	.	2	2	+	+	.	1	+
***Cytiso spinescentis-Saturejion montanae***																			
*Centaurea subtilis*	1	3	1	+	2	3	3	3	3	3	4	4	2	3	3	2	2	2	1
*Helianthemum oleandicum* subsp. *incanum*	+	1	1	1	1	+	2	1	1	1	1	+	+	+	1	+	+	+	+
*Cytisus spinescens*	2	1	+	+	+	.	+	+	+	+	+	+	1	1	1	1	1	1	1
*Satureja montana* subsp. *montana*	+	+	.	+	+	+	+	+	+	.	.	.	3	3	1	2	2	1	2
*Rhamnus saxatilis*	+	+	+	+	+	.	.	+	.	+	+	.	+	+	.	2	1	2	2
*Alyssum diffusum* subsp. *garganicum*	+	+	+	.	+	.	.	.	+	+	.	.	1	+	1	+	2	1	2
***Cisto-Ericetalia manipuliflorae* and *Cisto-Micromerietea***																			
*Thymbra capitata*	3	2	4	2	2	3	2	3	1	3	2	1	2	2	3	3	2	3	3
*Linum tommasinii*	+	+	+	+	.	+	+	+	+	+	+	+	2	1	1	2	2	+	2
*Teucrium capitatum* subsp. *capitatum*	+	+	+	+	+	+	+	+	+	+	+	+	+	.	.	+	+	+	+
*Leontodon apulus*	+	+	+	+	+	+	.	.	.	+	+	+	1	1	+	1	+	1	1
*Hippocrepis glauca*	+	+	1	1	+	.	.	.	.	+	+	+	+	1	1	1	1	1	+
*Asyneuma limonifolium* subsp. *limonifolium*	+	+	+	.	.	.	.	+	+	.	.	.	1	+	+	1	1	+	1
*Fumana thymifolia*	+	1	2	+	+	1	1	2	2	1	2	1	.	.	.	.	.	.	.
*Helianthemum jonium*	.	.	.	.	.	.	.	.	.	.	.	.	2	2	1	2	3	1	2
*Dianthus tarentinus*	.	.	.	.	.	.	.	.	.	+	+	.	.	.	.	+	+	1	.
*Ononis pusilla*	+	.	+	+	+	.	.	.	.	+	.	.	.	.	.	.	.	.	.
*Fumana ericifolia*	.	.	.	.	.	.	.	.	.	.	.	.	.	.	+	.	.	+	.
*Cistus creticus* subsp. *eriocephalus*	.	.	.	1	.	.	.	.	.	.	.	.	.	.	.	.	.	.	.
*Micromeria graeca* subsp. *graeca*	.	.	.	.	.	.	.	.	.	.	.	.	.	.	.	.	.	.	+
**Other species**																			
*Stipa austroitalica* subsp. *austroitalica*	+	+	+	+	+	+	+	+	+	+	+	+	2	1	1	2	2	2	2
*Koeleria subcaudata*	+	+	+	+	+	+	1	+	+	+	+	+	.	.	+	1	+	1	+
*Onobrychis alba* subsp. *echinata*	+	+	+	+	1	.	.	+	+	.	.	.	+	+	+	+	+	+	.
*Carex flacca* subsp. *erythrostachys*	1	+	+	1	.	+	2	1	1	2	1	2	.	.	.	.	.	.	.
*Centaurium erythraea*	+	+	+	+	+	+	.	+	+	+	+	+	.	.	.	.	.	.	.
*Cynanchica aristata* subsp. *aristata*	.	+	.	.	.	.	+	+	+	+	+	+	.	.	+	+	.	+	.
*Festuca circummediterranea*	+	+	.	.	.	.	.	.	.	.	.	.	+	+	1	+	+	1	2
*Sabulina attica*	+	+	.	.	1	.	.	.	+	.	.	.	+	+	+	+	+	.	.
*Odontites luteus* subsp. *luteus*	+	+	+	+	.	+	.	+	+	.	+	+	.	.	.	.	.	.	.
*Anthyllis vulneraria* subsp. *rubriflora*	+	+	.	.	+	.	.	+	+	+	+	+	.	.	.	.	.	.	.
*Thesium humifusum*	+	+	+	+	+	+	.	.	.	+	.	.	.	.	.	.	.	.	.
*Petrosedum ochroleucum* subsp. *mediterraneum*	.	.	.	.	.	.	.	.	.	.	.	.	+	1	1	1	1	1	+
*Helianthemum croceum*	.	.	.	.	.	.	.	.	.	.	.	.	1	1	2	1	1	+	1
*Linum strictum*	+	+	+	+	+	+	.	.	.	+	.	.	.	.	.	.	.	.	.
*Poterium sanguisorba*	+	.	.	.	.	.	.	.	.	.	.	.	+	.	+	+	+	+	+
*Asphodelus ramosus* subsp. *ramosus*	.	.	.	.	.	.	.	.	.	.	.	.	1	+	+	+	+	+	1
*Allium sphaerocephalon*	.	.	.	.	.	.	.	.	.	.	.	.	+	+	+	+	+	+	+
*Reseda phyteuma*	.	.	.	.	.	.	.	.	.	.	.	.	1	1	1	1	1	1	1
*Muscari* sp.	.	.	.	.	.	.	.	.	.	.	.	.	+	+	+	+	+	+	+
*Ranunculus millefoliatus*	.	.	.	.	.	.	.	.	.	.	.	.	+	+	+	+	1	+	+
*Jurinea mollis* subsp. *mollis*	+	+	+	.	.	+	.	.	.	.	.	.	.	.	.	.	.	+	+
*Reichardia picroides*	.	.	.	.	.	.	.	.	.	.	.	.	+	+	.	+	+	+	+
*Alkanna tinctoria* subsp. *tinctoria*	.	.	.	.	.	.	.	.	.	.	.	.	+	+	.	+	+	+	+
*Poa bulbosa*	.	.	.	.	.	.	.	.	.	.	.	.	1	2	.	1	2	1	1
*Ophrys fusca*	.	.	.	.	.	.	.	.	.	.	.	.	+	+	+	+	.	+	+
*Euphorbia nicaeensis* subsp. *japygica*	+	+	+	+	+	.	.	.	.	.	.	.	.	.	.	.	.	.	.
*Centaurea deusta* s.l.	.	.	.	.	.	.	.	.	.	.	.	.	+	+	.	+	.	+	+
*Asphodeline lutea*	.	.	.	.	.	.	.	.	.	.	.	.	1	+	+	.	+	1	.
*Eryngium amethystinum*	+	.	.	.	.	.	.	.	.	.	.	.	.	.	.	.	+	1	+
*Carex caryophyllea*	.	.	.	.	.	.	.	.	.	.	.	.	.	.	1	+	+	+	.
*Neotinea tridentata*	.	.	.	.	.	.	.	.	.	.	.	.	+	+	.	.	+	+	.
*Stachys romana*	+	+	.	.	+	.	.	.	.	.	.	.	.	.	.	.	.	.	.
*Carduus nutans*	.	.	.	.	.	.	.	.	.	.	.	.	+	+	+	.	.	.	.
*Clinopodium suaveolens*	.	+	.	.	.	.	.	.	+	.	.	.	.	.	.	.	.	.	.
*Ononis reclinata*	.	+	.	.	.	.	.	.	+	.	.	.	.	.	.	.	.	.	.
*Thymus spinulosus*	.	+	.	+	.	.	.	.	.	.	.	.	.	.	.	.	.	.	.
*Bupleurum baldense*	+	.	+	.	.	.	.	.	.	.	.	.	.	.	.	.	.	.	.
*Hippocrepis ciliata*	+	+	.	.	.	.	.	.	.	.	.	.	.	.	.	.	.	.	.
*Juniperus oxycedrus*	.	+	+	.	.	.	.	.	.	.	.	.	.	.	.	.	.	.	.
*Bromopsis erecta* subsp. *erecta*	.	.	.	.	.	.	+	+	.	.	.	.	.	.	.	.	.	.	.
*Helianthemum nummularium*	.	.	.	.	.	.	.	.	.	.	.	.	.	.	.	.	.	+	+
*Anchusa undulata* subsp. *hybrida*	.	.	.	.	.	.	.	.	.	.	.	.	+	.	.	.	.	+	.
*Reseda alba*	.	.	.	.	.	.	.	.	.	.	.	.	.	.	.	1	+	.	.
*Crepis vesicaria*	.	.	.	.	.	.	.	.	.	.	.	.	.	.	.	.	.	+	+
*Crupina crupinastrum*	+	.	.	.	.	.	.	.	.	.	.	.	.	.	.	.	.	.	.

**Table A18:** *Fumano ericifoliae-Centaureetum subtilis* Tomaselli & Costanzo ass. nova.

Relevé Number	1	2	3	4	5	6	7	8	9	10	11	12	13	14	15	16	17
**Typus**						X											
**Surface (m^2^)**	60	50	50	45	60	50	50	60	80	80	15	40	60	70	50	15	15
**Cover (%)**	80	90	75	80	85	80	45	50	50	80	60	50	80	50	90	55	55
**Altitude (m)**	677	650	626	668	696	670	335	460	210	240	680	468	658	542	670	670	690
**Exposure**	SSO	S	-	SSO	S	S	O	S	SO	O	OSO	O	NE	S	OSO	O	SO
**Slope (°)**	10	5	-	15	50	10	40	25	45	40	20	15	30	10	20	20	20
**Rockiness**	10	5	30	20	10	10	40	25	40	5	20	5	30	40	5	20	25
**Stoniness**	80	90	50	70	90	80	15	25	10	15	70	40	5	10	10	70	60
**Average vegetation height (cm)**	-	-	-	-	-	-	25	20	25	60	20	20	20	25	50	20	20
**Diagnostic species**																	
*Fumana ericifolia*	1	+	.	1	2	2	1	1	1	+	3	2	2	+	1	2	2
*Hippocrepis comosa*	+	1	.	+	+	+	+	1	.	+	+	.	+	+	+	.	+
*Scabiosa garganica*	.	+	+	1	1	+	.	.	.	.	.	.	.	.	.	.	.
**Diff. subassociation**																	
*Genista michelii*	.	.	.	.	.	.	.	.	.	.	.	+	1	1	1	2	1
***Cytiso spinescentis-Saturejion montanae***																	
*Centaurea subtilis*	1	1	+	+	2	2	1	2	2	+	2	2	2	2	+	1	2
*Helianthemum oelandicum* subsp. *incanum*	2	1	1	+	1	2	1	2	.	.	1	+	1	1	+	+	1
*Rhamnus saxatilis*	+	+	.	+	.	.	.	+	+	.	.	.	.	+	+	+	+
*Satureja montana* subsp. *montana*	+	1	+	+	2	1	.	.	.	.	.	.	+	+	.	.	.
*Cytisus spinescens*	.	.	.	.	.	.	+	+	+	.	.	.	.	+	.	.	.
*Alyssum diffusum* subsp. *garganicum*	+	.	.	.	.	+	.	.	.	.	.	.	.	.	.	.	.
**Trasgr. *Cisto eriocephali-Ericion multflorae***																	
*Cistus creticus* subsp. *eriocephalus*	+	.	+	.	.	.	.	.	.	1	+	.	+	.	2	+	.
*Dianthus tarentinus*	.	.	.	.	.	+	.	.	+	.	.	.	.	.	.	.	.
***Cisto-Ericetalia manipuliflorae* and *Cisto-Micromerietea***																	
*Thymbra capitata*	4	4	4	3	3	3	3	2	1	3	1	3	2	2	4	+	1
*Fumana thymifolia*	2	1	2	3	2	1	1	.	+	+	2	+	1	.	3	1	+
*Leontodon apulus*	+	+	1	+	1	.	+	+	.	+	.	.	+	+	1	.	+
*Satureja cuneifolia*	.	+	.	.	+	.	2	3	2	+	3	.	2	3	+	3	3
*Teucrium capitatum* subsp. *capitatum*	+	.	+	+	+	.	.	+	.	+	+	.	+	.	+	.	+
*Helianthemum jonium*	+	1	+	+	+	+	+	+	.	.	+	.	.	.	1	+	1
*Micromeria graeca* subsp. *graeca*	+	1	+	.	+	+	.	+	.	+	.	.	.	+	.	.	.
*Rosmarinus officinalis*	.	.	2	.	.	.	2	+	2	2	.	1	.	2	.	.	.
*Micromeria juliana*	+	+	.	+	+	+	+	.	.	.	.	.	.	+	.	.	.
*Helichrysum italicum* subsp. *italicum*	1	1	+	+	+	+	.	.	.	.	.	.	.	.	.	.	.
*Ononis pusilla*	+	+	.	+	.	.	.	.	.	.	.	.	.	.	.	+	+
*Euphorbia spinosa*	.	2	1	+	.	.	.	.	.	.	+	.	.	.	2	.	.
**Other species**																	
*Koeleria subcaudata*	2	2	1	1	3	2	+	1	1	+	1	1	+	1	2	+	1
*Festuca circummediterranea*	2	1	1	2	2	1	+	+	+	.	+	+	+	1	1	.	+
*Thesium humifusum*	+	+	.	+	+	.	+	+	+	.	1	+	1	+	1	+	1
*Cynanchica aristata* subsp. *aristata*	1	+	.	+	+	+	+	1	.	.	+	+	+	+	.	1	1
*Stipa austroitalica* subsp. *austroitalica*	.	+	.	.	+	.	+	+	+	+	2	1	.	+	2	1	1
*Linum strictum*	+	1	.	.	.	+	+	+	+	.	+	+	+	+	+	.	+
*Onobrychis alba* subsp. *echinata*	+	1	+	1	1	+	.	.	.	.	+	.	+	.	1	1	1
*Sabulina attica*	+	.	1	+	1	+	.	.	.	+	+	.	.	+	1	1	1
*Galium corrudifolium*	+	1	+	+	+	+	.	.	.	.	.	.	.	.	+	+	.
*Linum tenuifolium*	.	.	.	.	.	.	.	+	.	+	+	1	.	+	.	+	1
*Hypochaeris achyrophorus*	+	+	.	.	.	.	+	.	.	.	+	.	+	+	+	.	.
*Crupina crupinastrum*	+	+	+	.	+	.	.	+	.	.	.	.	+	.	.	.	.
*Brachypodium retusum*	2	1	1	+	1	2	.	.	.	.	.	.	.	.	.	.	.
*Carlina corymbosa*	+	1	+	1	+	+	.	.	.	.	.	.	.	.	.	.	.
*Stachys italica*	+	1	+	+	1	+	.	.	.	.	.	.	.	.	.	.	.
*Petrosedum ochroleucum* subsp. *mediterraneum*	.	.	.	.	.	.	+	+	+	+	.	.	.	.	.	+	.
*Centaurium erythraea*	.	.	.	.	.	.	+	.	.	.	+	+	+	.	.	.	+
*Clinopodium suaveolens*	+	+	+	.	+	+	.	.	.	.	.	.	.	.	.	.	.
*Ononis reclinata*	.	.	.	.	.	.	.	.	+	.	.	.	+	.	+	+	+
*Blackstonia perfoliata* subsp. *perfoliata*	+	+	+	+	1	.	.	.	.	.	.	.	.	.	.	.	.
*Macrobriza maxima*	+	+	+	.	+	.	.	.	.	.	.	.	.	.	+	.	.
*Reichardia picroides*	+	+	.	+	.	.	.	+	.	.	.	.	.	+	.	.	.
*Centaurium pulchellum* subsp. *pulchellum*	+	.	+	+	.	.	.	.	.	.	.	.	.	.	.	+	+
*Anthyllis vulneraria* subsp. *rubriflora*	.	+	.	+	+	.	.	.	.	.	.	.	+	.	.	.	.
*Sixalix atropurpurea* subsp. *maritima*	+	+	.	.	+	.	.	.	.	.	.	.	.	+	.	.	.
*Juniperus oxycedrus*	+	+	.	+	.	.	.	.	.	.	.	.	.	.	+	.	.
*Anthyllis vulneraria* subsp. *maura*	.	.	.	.	.	.	.	.	.	.	+	.	.	.	1	+	+
*Lomelosia crenata* subsp. *dallaportae*	.	.	.	.	.	.	+	.	+	.	.	.	2	.	.	.	.
*Carex flacca* subsp. *erythrostachys*	.	.	.	.	.	.	.	.	.	.	.	.	.	.	+	+	+
*Odontites luteus* subsp. *luteus*	.	.	.	.	.	.	.	.	.	.	+	.	.	.	+	.	+
*Hippocrepis ciliata*	.	.	.	.	.	.	.	+	+	+	.	.	.	.	.	.	.
*Xeranthemum inapertum*	+	+	.	+	.	.	.	.	.	.	.	.	.	.	.	.	.
*Trifolium scabrum*	.	.	.	.	.	.	.	.	.	.	.	.	.	.	+	+	+
*Allium apulum*	.	.	.	.	.	.	.	.	+	.	.	.	+	+	.	.	.
*Lagurus ovatus*	+	.	+	.	+	.	.	.	.	.	.	.	.	.	.	.	.
*Pistacia lentiscus*	.	.	.	.	.	.	+	.	+	+	.	.	.	.	.	.	.
*Inula verbascifolia*	.	.	.	.	.	.	+	.	.	.	.	.	+	.	.	.	.
*Thymus spinulosus*	.	.	.	.	.	+	+	.	.	.	.	.	.	.	.	.	.
*Jurinea mollis* subsp. *mollis*	.	.	.	.	.	.	.	.	.	+	.	.	+	.	.	.	.
*Bellardia trixago*	.	.	.	.	.	.	.	.	.	.	.	.	.	.	+	+	.
*Onobrychis caput-galli*	+	.	+	.	.	.	.	.	.	.	.	.	.	.	.	.	.
*Linum usitatissimum* subsp. *angustifolium*	.	.	.	.	.	.	.	.	.	.	.	.	+	.	+	.	.
*Petrorhagia saxifraga* subsp. *saxifraga*	.	.	.	.	.	.	+	.	.	.	.	.	+	.	.	.	.
*Pinus halepensis* subsp. *halepensis*	.	.	.	.	.	.	.	+	.	.	.	+	.	.	.	.	.
*Dactylis glomerata* subsp. *hispanica*	+	.	.	.	+	.	.	.	.	.	.	.	.	.	.	.	.
*Onobrychis aequidentata*	.	.	.	.	.	.	.	+	.	.	.	.	+	.	.	.	.

**Table A19:** *Chamaecityso spinescentis-Genistetum michelii* De Faveri & Nimis ex Biondi 2000.

Relevé Number	1	2	3	4	5	6	7	8	9	10	11	12	13	14	15	16
**Typus**		X														
**Surface (m^2^)**	-	-	-	-	-	-	-	-	-	-	80	50	50	50	50	50
**Cover (%)**	-	-	-	-	-	-	-	-	-	-	80	70	80	60	70	70
**Altitude (m)**	-	-	-	-	-	-	-	-	-	-	667	620	636	630	680	650
**Exposure**	-	-	-	-	-	-	-	-	-	-	NE	O	O	O	NO	NO
**Slope (°)**	-	-	-	-	-	-	-	-	-	-	25	10	30	30	40	40
**Rockiness**	-	-	-	-	-	-	-	-	-	-	15	20	-	-	-	-
**Stoniness**	-	-	-	-	-	-	-	-	-	-	5	5	-	-	-	-
**Average vegetation height (cm)**	-	-	-	-	-	-	-	-	-	-	25	30	-	-	-	-
**Diagnostic species**																
*Genista michelii*	1	1	3	1	1	2	1	1	1	1	2	2	3	3	3	2
***Cytiso spinescentis-Saturejion montanae***																
*Cytisus spinescens*	2	2	+	2	2	2	3	3	3	2	.	1	.	.	2	3
*Helianthemum oelandicum* subsp. *incanum*	+	+	.	+	+	1	.	+	+	+	2	1	2	1	2	2
*Satureja montana* subsp. *montana*	.	.	.	.	.	.	.	.	.	.	1	+	1	1	1	+
*Rhamnus saxatilis*	.	.	.	.	.	.	.	.	.	.	+	+	+	.	.	.
*Scabiosa garganica*	.	.	.	.	.	.	.	.	.	.	.	.	2	2	2	1
*Centaurea subtilis*	.	.	.	.	.	.	.	.	.	.	.	.	.	.	1	2
***Cisto-Ericetalia manipuliflorae* and *Cisto-Micromerietea***																
*Satureja cuneifolia*	1	+	+	+	1	1	.	.	.	+	2	2	2	3	2	2
*Rosmarinus officinalis*	+	3	2	3	3	2	2	2	2	2	.	.	.	.	3	2
*Euphorbia spinosa*	1	+	+	+	.	.	+	.	.	1	.	1	.	.	.	.
*Helianthemum jonium*	+	+	+	.	.	.	1	.	.	.	+	.	.	.	2	2
*Fumana thymifolia*	.	.	.	+	1	+	.	+	+	.	2	+	.	.	.	.
*Teucrium capitatum* subsp. *capitatum*	.	.	.	.	.	.	+	.	.	+	.	.	+	+	+	1
*Thymbra capitata*	.	.	.	.	.	.	.	.	.	.	3	3	2	1	2	1
*Dianthus tarentinus*	.	.	.	.	.	.	.	.	.	.	+	.	+	+	+	+
*Fumana ericifolia*	.	.	.	.	.	.	.	.	.	.	2	1	.		1	+
*Cistus creticus* subsp. *eriocephalus*	.	.	.	.	.	.	.	.	.	.	+	+	.	.	.	.
*Hippocrepis comosa*	.	.	.	.	.	.	.	.	.	.	+	+	.	.	.	.
*Leontodon apulus*	.	.	.	.	.	.	.	.	.	.	+	+	.	.	.	.
*Micromeria graeca* subsp. *graeca*	.	.	.	.	.	.	.	.	.	.	.	.	.	.	+	1
*Micromeria juliana*	.	.	.	.	.	.	.	.	.	.	.	+	.	.	.	.
**Other species**																
*Koeleria subcaudata*	+	+	+	+	+	+	+	.	+	+	1	1	+	+	2	1
*Leontodon crispus*	+	+	+	+	+	+	+	+	+	+	.	.	1	+	1	+
*Galium lucidum*	+	+	+	+	+	+	+	.	.	+	.	.	2	1	1	1
*Polygala nicaeensis*	+	+	+	+	+	+	+	+	+	+	.	.	.	.	.	.
*Dactylis glomerata* subsp. *hispanica*	+	.	.	.	.	+	+	+	+	+	.	.	1	+	+	+
*Carex liparocarpos* subsp. *liparocarpos*	+	+	.	.	.	+	+	+	.	.	.	.	1	+	1	2
*Festuca circummediterranea*	+	.	.	+	+	+	.	.	+	.	.	.	3	2	1	1
*Acinos alpinus*	.	+	.	+	.	.	1	.	.	+	.	.	1	1	1	+
*Stipa austroitalica* subsp. *austroitalica*	+	.	.	.	+	.	.	.	.	.	+	1	3	1	2	3
*Brachypodium retusum*	+	+	+	+	+	+	.	.	.	+	.	.	.	.	.	.
*Cynanchica garganica*	.	.	.	.	.	.	+	+	.	.	.	.	1	+	+	1
*Carex flacca* subsp. *erythrostachys*	+	+	.	1	.	+	.	.	.	.	.	.	.	.	+	+
*Anthyllis vulneraria* subsp. *rubriflora*	.	.	.	.	.	.	.	.	.	.	+	1	1	+	+	+
*Pimpinella tragium*	+	.	+	+	.	+	.	.	.	+	.	.	.	.	.	.
*Linum strictum*	.	.	.	.	.	.	.	.	.	.	+	+	+	+	+	.
*Marrubium incanum*	.	+	.	+	.	+	+	.	.	.	.	.	.	.	.	.
*Centaurium erythraea*	.	.	.	.	.	+	.	.	+	.	.	.	.	.	+	+
*Carlina corymbosa*	.	.	.	.	.	.	.	+	.	+	.	.	+	+	.	.
*Bromopsis ramosa*	.	.	.	.	.	.	.	.	.	.	.	.	+	+	1	+
*Picris hieracioides* subsp. *hieracioides*	.	.	.	.	.	.	.	.	.	.	.	.	+	+		
*Echinops ritro*	.	.	.	.	.	.	.	.	.	.	.	.	1	1	2	1
*Centaurea deusta* s.l.	.	.	.	.	.	.	.	.	.	.	.	.	1	+	1	+
*Bromopsis erecta* subsp. *erecta*	.	.	.	.	.	.	.	.	.	.	+	+	1	1	.	.
*Allium apulum*	.	.	.	.	.	.	.	.	.	.	.	.	+	+	+	+
*Phleum* sp.	.	.	.	.	.	.	.	.	.	.	.	.	1	+	1	1
*Erysimum* sp.	.	.	.	.	.	.	.	.	.	.	.	.	+	+	1	+
*Onobrychis alba* subsp. *echinata*	.	.	.	.	.	.	.	.	.	.	+	+	.	.	+	+
*Poa bulbosa* subsp. *bulbosa*	.	.	+	.	+	.	+	.	.	.	.	.	.	.	.	.
*Hypochaeris achyrophorus*	.	.	+	.	.	.	+	.	.	.	.	+	.	.	.	.
*Convolvulus elegantissimus*	.	.	.	.	.	.	.	.	.	+	.	.	+	+	.	.
*Petrorhagia saxifraga* subsp. *saxifraga*	.	.	.	.	.	.	.	.	.	.	+	.	1	+	.	.
*Lomelosia crenata* subsp. *dallaportae*	.	.	.	.	.	.	.	.	.	.	+	.	.	.	+	+
*Thesium humifusum*	.	.	.	.	.	.	.	.	.	.	1	+	.	.	.	.
*Inula verbascifolia*	.	.	.	.	.	.	.	.	.	.	+	+	.	.	.	.
*Cynanchica aristata* subsp. *aristata*	.	.	.	.	.	.	.	.	.	.	+	+	.	.	.	.
*Ononis reclinata*	.	.	.	.	.	.	.	.	.	.	+	+	.	.	.	.
*Verbascum niveum* subsp. *garganicum*	.	.	.	.	.	.	.	.	.	.	.	.	1	+	.	.
*Sideritis italica*	.	.	.	.	.	.	.	.	.	.	.	.	2	2	.	.
*Silene vulgaris* subsp. *tenoreana*	.	.	.	.	.	.	.	.	.	.	.	.	1	1	.	.
*Eryngium amethystinum*	.	.	.	.	.	.	.	.	.	.	.	.	2	2	.	.
*Koeleria subcaudata*	.	.	.	.	.	.	.	.	.	.	.	.	1	+	.	.
*Pallenis spinosa* subsp. *spinosa*	.	.	.	.	.	.	.	.	.	.	.	.	+	+	.	.
*Hyoseris radiata*	.	.	.	.	.	.	.	.	.	.	.	.	+	+	.	.
*Reichardia picroides*	.	.	.	.	.	.	.	.	.	.	.	.	+	+	.	.
*Plantago lanceolata*	.	.	.	.	.	.	.	.	.	.	.	.	+	+	.	.
*Sixalix atropurpurea* subsp. *maritima*	.	.	.	.	.	.	.	.	.	.	.	.	+	+	.	.
*Thymus spinulosus*	.	.	.	.	.	.	.	.	.	.	.	.	1	+	.	.
*Nonea echioides*	.	.	.	.	.	.	.	.	.	.	.	.	+	+	.	.
*Thesium humifusum*	.	.	.	.	.	.	.	.	.	.	.	.	1	1	.	.
*Teucrium montanum*	.	.	.	.	.	.	.	.	.	.	.	.	.	.	1	2
*Brachypodium retusum*	.	.	.	.	.	.	.	.	.	.	.	.	.	.	2	2
*Leontodon intermedius*	.	.	.	.	.	+	.	.	.	.	.	.	.	.	.	.

**Table A20:** *Centaureo tenacissimae-Euphorbietum spinosae* Minissale, Giusso & Brullo ass. nova.

Relevé Number	1	2	3	4
**Typus**		X		
**Surface (m^2^)**	30	20	20	20
**Cover (%)**	70	70	80	90
**Altitude (m)**	480	440	470	480
**Diagnostic species**				
*Euphorbia spinosa*	2	3	2	1
*Centaurea tenacissima*	1	2	1	1
*Aurinia sinuata*	2	2	1	.
*Onosma echioides* subsp. *angustifolia*	.	1	+	2
***Cytiso spinescentis-Saturejion montanae***				
*Rhamnus saxatilis*	2	1	+	+
*Cytisus spinescens*	.	+	1	2
*Centaurea subtilis*	.	.	+	1
**Trasgr. *Cisto eriocephali-Ericion multiflorae***				
*Phagnalon rupestre* subsp. *illyricum*	+	+	.	.
*Cistus creticus* subsp. *eriocephalus*	.	.	.	1
*Dianthus tarentinus*	.	.	+	.
***Cisto-Ericetalia manipuliflorae* and *Cisto-Micromerietea***				
*Satureja cuneifolia*	3	3	3	3
*Fumana ericifolia*	2	1	1	2
*Linum tommasinii*	+	1	+	1
*Fumana thymifolia*	1	1	+	+
*Thymbra capitata*	1	+	1	+
*Micromeria graeca* subsp. *graeca*	1	1	1	1
*Micromeria juliana*	.	.	+	.
**Other species**				
*Stipa austroitalica* subsp. *austroitalica*	2	1	2	2
*Petrosedum ochroleucum* subsp. *mediterraneum*	1	+	1	+
*Allium apulum*	+	+	+	1
*Allium sardoum*	+	+	+	+
*Thesium humifusum*	+	+	1	1
*Allium subhirsutum* subsp. *subhirsutum*	1	1	+	+
*Asparagus acutifolius*	1	+	+	+
*Asphodelus ramosus* subsp. *ramosus*	1	1	1	+
*Convolvulus altheoides*	+	+	1	+
*Eryngium amethystinum*	+	1	+	1
*Galium lucidum*	1	2	2	2
*Scorzonera villosa* subsp. *columnae*	+	1	+	1
*Jurinea mollis* subsp. *mollis*	+	+	1	2
*Silene vulgaris* subsp. *tenoreana*	2	1	1	+
*Stachys recta* subsp. *subcrenata*	+	1	1	2
*Stachys romana*	+	+	+	+
*Anagyris foetida*	1	2	1	.
*Andropogon distachyos*	+	.	3	1
*Anthyllis vulneraria* subsp. *maura*	+	+	.	1
*Bellardia trixago*	+	+	+	.
*Trachynia distachya*	+	1	+	.
*Euphorbia characias*	+	2	+	.
*Festuca circummediterranea*	.	1	2	3
*Hyparrhenia hirta*	+	+	.	1
*Iris pseudopumila*	1	+	+	.
*Koeleria subcaudata*	1	+	.	1
*Melica ciliata*	+	+	+	.
*Pallenis spinosa* subsp. *spinosa*	+	+	+	.
*Petrorhagia saxifraga* subsp. *saxifraga*	1	+	.	+
*Rhamnus alaternus* subsp. *alaternus*	1	1	1	.
*Stachys major*	+	1	+	.
*Verbascum* sp.	.	1	+	+
*Anethum piperitum*	.	.	+	1
*Brachypodium retusum*	.	.	+	+
*Centaurea sicula*	+	1	.	.
*Hyoseris radiata*	+	+	.	.
*Melilotus* sp.	.	+	.	1
*Olea europaea*	1	2	.	.
*Ononis mitissima*	.	+	+	.
*Pistacia lentiscus*	+	.	+	.
*Reichardia picroides*	.	.	+	+
*Sedum acre*	+	.	+	.
*Teucrium montanum*	.	+	.	1
*Thapsia garganica*	+	.	+	.
*Asphodeline lutea*	.	+	.	.
*Coronilla valentina*	.	1	.	.
*Crepis vesicaria*	.	+	.	.
*Daucus carota* s.l.	.	+	.	.

**Table A21:** Rhamno saxatili-Saturejetum montanae Tomaselli, Silletti, Forte 2021; typicum (1–13), fumanetosum procumbentis (14–20), asyneumetosum limonifolium (21–27).

Relevé Number	1	2	3	4	5	6	7	8	9	10	11	12	13	14	15	16	17	18	19	20	21	22	23	24	25	26	27
**Typus**																					X						
**Surface (m^2^)**	30	20	20	T	50	80	70	70	80	30	25	50	50	50	40	40	70	50	50	60	10	15	50	40	30	60	80
**Cover (%)**	60	60	50	60	60	70	75	80	75	80	70	65	70	80	70	80	70	75	65	60	75	80	70	70	60	65	70
**Altitude (m)**	560	562	550	560	475	495	550	510	500	480	500	495	510	515	485	485	485	430	470	470	340	330	435	445	348	345	365
**Exposure**	O	ONO	O	NO	OSO	O	SO	E	O	SO	S	SO	SO	S	S	S	E	NE	SO	NE	SSE	S	NNO	-	O	O	-
**Slope (°)**	40	45	50	45	25	40	25	20	25	15	25	30	25	-	-	-	10	10	10	10	5	20	20	-	15	25	-
**Rockiness**	25	30	20	25	30	30	25	20	25	20	25	30	25	5	5	5	10	20	5	5	-	-	-	-	30	20	25
**Stoniness**	5	5	10	10	5	5	10	10	5	5	5	10	5	50	60	50	70	50	80	80	-	-	-	-	5	10	10
**Average vegetation height (cm)**	30	25	25	25	25	30	30	25	25	30	30	25	30	40	30	40	30	35	25	25	-	-	-	-	20	20	25
**Diagnostic species**																											
*Euphorbia nicaeensis* subsp. *japygica*	1	+	+	+	+	.	+	+	+	+	+	+	+	1	1	1	+	+	+	1	.	.	.	.	.	.	.
*Ruta graveolens*	.	1	+	.	1	+	1	+	.	1	2	1	+	.	.	.	.	.	+	+	.	.	.	.	.	.	.
*Allium apulum*	.	+	.	+	.	2	1	2	1	.	.	+	.	+	+	+	.	.	.	.	.	.	.	.	.	.	.
*Centaurea brulla*	.	.	.	+	+	+	.	.	.	.	.	.	.	+	+	.	.	.	.	.	.	.	.	.	+	+	.
**Diff. subassociation**																											
*Fumana procumbens*	.	.	.	.	.	.	.	.	.	.	.	.	.	2	2	1	2	3	2	3	.	.	.	.	.	.	.
*Odontites luteus*	.	.	.	.	.	.	.	.	.	.	.	.	.	+	+	.	+	+	+	+	.	.	.	.	.	.	.
*Ornithogalum gussonei*	.	.	.	.	.	.	.	.	.	.	.	.	.	.	.	.	+	+	+	+	.	.	.	.	.	.	.
*Asyneuma limonifolium* subsp. *limonifolium*	.	.	.	.	.	.	.	.	.	.	.	.	.	.	.	.	+	+	.	.	1	1	+	1	+	1	+
*Phagnalon rupestre* subsp. *illyricum*	.	.	.	.	.	.	.	.	.	.	.	.	.	.	.	.	.	.	.	.	+	1	1	+	1	1	.
*Cistus creticus* subsp. *eriocephalus*	.	.	.	.	.	.	.	.	.	.	.	.	.	.	.	.	.	.	.	.	.	.	.	.	+	1	+
***Cytiso spinescentis-Saturejion montanae***																											
*Satureja montana* subsp. *montana*	3	4	4	3	3	4	4	4	4	4	4	4	4	4	3	4	4	4	3	3	4	4	4	5	3	4	4
*Rhamnus saxatilis*	2	2	2	3	3	2	3	3	3	4	3	3	2	.	1	2	+	+	+	1	.	.	.	.	1	+	.
**Trasgr. *Cisto eriocephali-Ericion multiflorae***																											
*Dianthus tarentinus*	1	1	+	1	1	1	+	1	1	.	+	.	.	.	.	.	.	.	.	.	.	.	.	.	.	.	.
***Cisto-Ericetalia manipuliflorae* and *Cisto-Micromerietea***																											
*Teucrium capitatum* subsp. *capitatum*	+	1	+	+	1	+	1	+	+	1	+	2	1	1	1	1	1	+	+	+	+	.	1	1	1	1	+
*Micromeria graeca* subsp. *graeca*	2	1	1	1	2	2	1	2	2	1	1	1	+	.	+	.	.	.	.	.	+	.	.	+	1	1	.
*Fumana thymifolia*	.	.	.	.	.	.	.	.	2	.	.	.	1	2	1	2	2	1	2	2	1	.	.	+	2	1	1
*Helianthemum jonium*	.	.	.	.	.	.	.	.	1	.	.	.	2	1	2	+	+	1	+	+	.	.	.	.	1	1	+
*Hippocrepis glauca*	.	.	.	.	.	.	.	.	.	.	.	.	.	2	.	1	+	1	1	+	.	.	.	.	.	.	.
*Euphorbia spinosa*	.	.	.	.	.	+	2	.	.	1	1	.	.	.	1	+	.	.	.	.	.	.	.	.	.	.	.
*Linum tommasinii*	+	+	+	+	.	.	.	.	.	.	.	.	.	.	.	.	.	+	.	.	.	.	.	.	.	.	.
*Leontodon apulus*	.	.	.	.	.	.	.	.	.	.	.	.	.	.	.	.	+	+	1	1	.	.	.	.	.	.	.
*Hippocrepis comosa*	.	.	.	.	.	.	.	.	.	.	.	.	.	.	.	.	.	.	.	.	.	.	+	.	1	2	1
*Ononis pusilla*	.	.	.	.	.	.	.	.	.	.	.	.	.	+	.	.	.	+	.	+	.	.	.	.	.	.	.
*Fumana ericifolia*	.	.	.	.	.	.	.	.	.	.	.	.	1	.	.	.	.	.	.	.	.	.	.	.	1	+	.
*Fumana scoparia*	.	.	.	.	.	.	.	.	.	.	.	.	.	.	.	.	.	.	.	.	.	.	.	.	1	1	1
*Thymbra capitata*	.	.	.	.	.	.	.	.	.	.	.	.	.	.	.	.	.	.	.	.	.	.	.	.	.	.	+
**Other species**																											
*Stipa austroitalica* subsp. *austroitalica*	+	1	1	1	+	2	3	3	2	1	2	2	2	1	1	2	2	1	2	2	+	.	+	+	1	1	+
*Convolvulus cantabrica*	1	+	+	.	1	+	+	1	+	+	+	+	1	+	.	+	+	.	+	+	.	.	.	+	+	+	.
*Petrosedum ochroleucum* subsp. *mediterraneum*	1	+	+	1	2	+	1	1	+	1	+	1	+	+	.	.	+	+	.	+	.	.	.	.	1	+	+
*Cynanchica aristata* subsp. *aristata*	+	+	.	+	2	1	2	1	1	+	+	2	1	.	+	1	+	+	.	+	.	1	+	.	.	.	.
*Koeleria subcaudata*	1	1	+	+	.	1	1	+	1	2	1	2	1	1	1	1	1	+	1	+	.	.	.	.	.	.	.
*Scorzonera villosa* subsp. *columnae*	2	2	1	1	.	1	1	.	.	+	1	+	1	1	1	1	+	1	+	.	+	.	.	.	1	+	.
*Thymus spinulosus*	+	+	+	.	1	.	1	.	.	2	1	1	1	1	2	+	1	+	.	+	.	.	+	.	1	.	.
*Festuca circummediterranea*	2	1	1	1	2	1	+	1	1	2	1	1	3	+	.	.	+	+	.	.	.	.	.	.	.	.	.
*Anthyllis vulneraria* subsp. *rubriflora*	+	+	+	.	.	.	.	.	.	+	1	+	1	2	2	1	1	1	1	1	.	.	.	.	.	.	1
*Petrorhagia saxifraga* subsp. *saxifraga*	+	+	1	1	1	1	.	.	.	.	.	1	+	+	+	+	+	.	.	.	.	.	+	+	.	.	+
*Stachys romana*	+	+	+	.	+	+	.	+	+	.	.	.	.	+	+	.	+	+	.	.	+	+	+	.	+	.	.
*Linum strictum*	.	+	+	.	1	.	1	.	.	.	.	+	+	.	.	.	+	1	+	.	+	.	.	+	+	+	.
*Reichardia picroides*	1	.	+	+	+	1	1	+	1	.	1	.	1	.	+	.	.	.	.	.	.	.	.	.	.	+	+
*Thapsia asclepium*	+	.	1	1	.	.	2	.	.	+	+	+	.	.	+	.	.	+	.	.	+	.	+	.	+	+	.
*Allium sphaerocephalon*	.	+	+	+	+	+	+	1	2	.	.	.	.	.	.	.	+	+	+	+	.	.	.	.	.	.	.
*Crupina crupinastrum*	.	1	+	+	.	.	.	+	+	.	+	.	+	+	+	.	.	.	+	.	.	.	.	.	+	+	.
*Helianthemum salicifolium*	+	+	+	.	1	1	+	+	1	.	.	.	.	.	.	.	.	+	.	.	.	.	.	.	1	1	.
*Hypochaeris achyrophorus*	.	.	.	.	+	+	1	.	+	+	+	1	+	.	.	.	.	.	.	.	.	.	.	.	1	1	+
*Teucrium chamaedrys* subsp. *chamaedrys*	.	+	+	.	+	+	+	+	+	2	.	.	+	.	.	.	.	.	+	.	+	.	.	.	.	.	.
*Clinopodium suaveolens*	+	+	.	.	+	+	.	+	.	2	+	1	2	.	.	.	+	.	.	.	.	.	.	.	.	.	.
*Bellardia trixago*	.	.	.	.	.	.	.	.	.	+	.	+	+	+	+	.	+	+	.	.	.	.	.	+	.	.	.
*Leontodon crispus*	1	+	+	+	.	.	.	.	.	.	.	.	.	2	1	1	.	.	.	.	.	.	.	.	.	.	1
*Linum trigynum*	.	.	.	.	1	1	.	1	1	.	.	.	.	.	.	.	+	1	+	+	.	.	.	.	.	.	.
*Sabulina attica*	.	.	.	.	.	.	1	+	1	.	.	.	1	+	.	.	+	.	+	+	.	.	.	.	.	.	.
*Stachys germanica* subsp. *salviifolia*	+	+	.	+	+	+	.	1	.	1	.	+	.	.	.	.	.	.	.	.	.	.	.	.	.	.	.
*Trifolium scabrum*	.	.	.	.	.	.	.	.	.	+	.	+	+	+	+	.	+	+	.	.	.	.	.	.	+	.	.
*Asphodelus ramosus* subsp. *ramosus*	.	.	.	.	.	.	.	.	.	.	.	+	1	+	1	+	+	.	.	+	.	.	.	.	.	.	.
*Asparagus acutifolius*	.	.	.	.	+	.	.	.	+	+	+	.	.	.	+	.	.	.	.	.	.	.	.	.	.	.	+
*Carex flacca* subsp. *erythrostachys*	.	.	.	.	.	.	.	.	.	.	.	.	.	1	+	1	.	.	.	.	.	.	.	.	+	+	+
*Eryngium amethystinum*	.	.	.	.	.	.	.	.	.	.	.	.	.	+	+	+	+	+	.	+	.	.	.	.	.	.	.
*Osyris alba*	.	.	.	.	.	.	1	+	1	.	.	.	.	.	+	.	.	.	1	1	.	.	.	.	.	.	.
*Centaurium erythraea*	.	.	.	.	+	.	+	.	+	.	.	.	.	.	.	.	.	.	.	.	.	.	.	.	.	+	+
*Centaurium pulchellum* subsp. *pulchellum*	.	.	.	.	.	.	.	.	.	.	.	.	.	+	+	+	.	.	+	+	.	.	.	.	.	.	.
*Hippocrepis ciliata*	+	.	.	.	.	.	.	.	.	.	.	.	.	.	.	.	+	+	+	+	.	.	.	.	.	.	.
*Onobrychis aequidentata*	.	.	.	.	.	.	.	.	.	.	.	.	.	.	.	.	+	+	.	.	.	.	.	.	+	+	1
*Onobrychis caput-galli*	.	.	.	.	.	.	.	.	.	.	.	.	.	.	+	+	+	.	.	.	.	.	.	.	+	.	+
*Ononis reclinata*	+	.	+	.	.	.	.	.	.	.	.	.	.	.	.	.	+	.	.	.	.	.	.	.	+	+	.
*Aurinia saxatilis* subsp. *megalocarpa*	+	+	+	+	.	.	.	.	.	.	.	.	.	.	.	.	.	.	.	.	.	.	.	.	.	.	.
*Trachynia distachya*	.	.	.	.	+	+	+	.	.	.	.	.	.	.	.	.	.	.	.	.	1	.	.	.	.	.	.
*Bromopsis erecta* subsp. *erecta*	.	.	.	.	.	.	.	.	.	.	2	1	.	+	.	.	.	1	.	.	.	.	.	.	.	.	.
*Squilla pancration*	.	.	.	.	.	.	.	.	.	.	.	.	.	.	+	+	.	.	.	.	.	+	1	.	.	.	.
*Eryngium campestre*	.	.	.	.	+	+	.	.	.	.	.	.	.	.	.	.	+	+	.	.	.	.	.	.	.	.	.
*Linum usitatissimum* subsp. *angustifolium*	.	.	.	.	.	.	.	.	.	.	.	.	.	.	.	.	+	+	.	.	.	.	.	.	.	+	+
*Macrobriza maxima*	.	.	.	.	+	+	.	+	.	.	.	.	.	.	.	.	.	.	.	.	.	.	.	.	.	.	.
*Bupleurum baldense*	.	.	.	.	.	.	.	.	.	.	.	.	.	+	.	.	.	+	+	.	.	.	.	.	.	.	.
*Convolvulus altheoides*	.	.	.	.	.	.	.	.	.	+	1	+	.	.	.	.	.	.	.	.	.	.	.	.	.	.	.
*Melica ciliata*	.	.	.	.	1	.	.	.	+	.	.	.	.	.	.	.	.	.	.	.	+	.	.	.	.	.	.
*Olea europaea*	.	+	.	.	.	.	.	.	.	.	.	.	.	.	.	.	.	.	.	.	+	.	.	.	+	.	.
*Petrosedum sediforme*	.	.	.	.	.	.	.	.	.	.	.	.	.	.	.	.	.	.	.	.	+	1	.	1	.	.	.
*Pistacia lentiscus*	.	.	.	.	.	.	.	.	.	.	.	.	.	.	.	.	.	.	.	.	.	.	.	.	+	+	+
*Sedum album* subsp. *micranthum*	1	.	1	1	.	.	.	.	.	.	.	.	.	.	.	.	.	.	.	.	.	.	.	.	.	.	.
*Thesium humifusum*	.	.	.	.	.	.	.	.	.	.	1	1	1	.	.	.	.	.	.	.	.	.	.	.	.	.	.
*Urospermum dalechampii*	.	.	.	.	.	.	.	.	.	.	.	.	.	+	.	.	+	+	.	.	.	.	.	.	.	.	.
*Ajuga chamaepitys*	+	+	.	.	.	.	.	.	.	.	.	.	.	.	.	.	.	.	.	.	.	.	.	.	.	.	.
*Anthyllis vulneraria* subsp. *maura*	.	.	.	.	.	.	.	1	1	.	.	.	.	.	.	.	.	.	.	.	.	.	.	.	.	.	.
*Carlina corymbosa*	.	.	.	.	.	.	.	.	.	.	.	.	.	+	+	.	.	.	.	.	.	.	.	.	.	.	.
*Convolvulus elegantissimus*	.	.	.	.	.	.	.	.	.	.	.	.	.	+	.	.	.	.	.	.	.	+	.	.	.	.	.
*Dactylis glomerata* subsp. *hispanica*	.	.	.	.	.	.	.	.	.	+	.	.	.	.	.	.	+	.	.	.	.	.	.	.	.	.	.
*Galium corrudifolium*	.	.	.	.	.	.	.	.	.	+	.	1	.	.	.	.	.	.	.	.	.	.	.	.	.	.	.
*Helictotrichon convolutum*	.	.	.	.	.	.	.	.	.	.	.	.	.	.	.	.	.	.	.	.	.	.	.	.	1	1	.
*Juniperus oxycedrus*	.	.	.	.	.	.	.	.	.	.	.	.	.	.	.	.	.	.	.	.	.	.	.	.	+	.	+
*Jurinea mollis* subsp. *mollis*	.	.	.	.	.	.	.	.	.	.	.	.	.	.	.	.	.	.	.	.	.	.	.	.	+	+	.
*Lagurus ovatus*	.	.	.	.	.	.	.	.	.	.	.	.	.	+	+	.	.	.	.	.	.	.	.	.	.	.	.
*Ononis ornithopodioides*	+	+	.	.	.	.	.	.	.	.	.	.	.	.	.	.	.	.	.	.	.	.	.	.	.	.	.
*Polygala monspeliaca*	.	.	.	.	.	.	.	.	.	.	.	.	.	.	.	.	.	.	.	.	.	.	.	.	+	.	+
*Silene conica*	.	.	.	.	.	.	.	.	.	.	.	.	.	+	.	.	+	.	.	.	.	.	.	.	.	.	.
*Silene vulgaris* subsp. *tenoreana*	.	.	.	.	.	.	.	.	.	.	.	.	.	.	+	.	.	.	.	.	.	.	.	.	+	.	.
*Sixalix atropurpurea* subsp. *maritima*	.	.	.	+	.	.	.	.	.	.	.	.	.	.	.	.	.	.	.	.	.	.	.	.	.	+	.
*Stachys major*	.	.	.	.	.	.	.	.	.	.	.	.	.	.	.	.	.	.	.	.	1	+	.	.	.	.	.
*Alkanna tinctoria* subsp. *tinctoria*	.	+	.	.	.	.	.	.	.	.	.	.	.	.	.	.	.	.	.	.	.	.	.	.	.	.	.
*Allium subhirsutum* subsp. *subhirsutum*	.	.	.	.	.	.	.	.	.	.	.	.	.	.	.	.	.	.	.	.	.	.	.	.	.	+	.
*Asphodeline liburnica*	.	.	.	.	.	.	.	.	.	.	.	.	.	+	.	.	.	.	.	.	.	.	.	.	.	.	.
*Coronilla valentina*	.	.	.	.	.	.	.	.	.	.	.	.	.	.	.	.	.	.	.	.	+	.	.	.	.	.	.
*Crepis vesicaria*	.	.	.	.	.	.	.	.	.	.	.	.	.	.	.	.	.	.	.	.	.	.	.	.	.	+	.
*Cytisus infestus*	.	.	.	.	.	.	.	.	.	.	.	.	.	.	.	.	.	.	.	.	.	.	.	.	.	.	+
*Galium lucidum*	.	.	.	.	.	.	.	.	.	.	.	.	.	+	.	.	.	.	.	.	.	.	.	.	.	.	.
*Onobrychis alba subsp. echinata*	.	.	.	.	.	.	.	.	.	.	.	.	.	.	.	.	2	.	.	.	.	.	.	.	.	.	.
*Petrosedum rupestre*	.	.	.	.	.	.	.	.	.	.	.	.	.	.	.	.	.	.	.	.	.	+	.	.	.	.	.
*Phillyrea latifolia*	.	.	.	.	.	.	.	.	.	.	.	.	.	.	.	.	.	.	.	.	+	.	.	.	.	.	.
*Poterium sanguisorba*	.	.	.	.	.	.	.	.	.	.	.	.	.	.	.	.	.	+	.	.	.	.	.	.	.	.	.
*Silene otites*	.	.	.	.	.	.	.	.	.	.	.	.	.	.	+	.	.	.	.	.	.	.	.	.	.	.	.
*Thesium humile*	.	.	.	.	.	.	.	.	+	.	.	.	.	.	.	.	.	.	.	.	.	.	.	.	.	.	.
*Trifolium campestre*	.	.	.	.	.	.	.	.	.	.	.	.	.	+	.	.	.	.	.	.	.	.	.	.	.	.	.
*Xeranthemum inapertum*	.	.	.	.	.	.	.	.	.	.	.	.	.	.	.	.	+	.	.	.	.	.	.	.	.	.	.

## Appendix C

Summary of the Kruskal–Wallis test applied to the difference in altitudinal distribution between plant communities.

## Appendix D

Summary table with the identified associations with some main ecological features and distribution.

**Association**	**Bioclimate**	**Lithology**	**Geographic Area**
*Loto commutati-Thymetum capitati*	upper thermomediterranean—upper dry to lower dry	sands	Salento, mostly western side
*Dauco gummiferis-Thymelaeetum hirsutae*	upper thermomediterranean—lower dry to lower subhumid	calcarenites	Salento, mostly eastern side
*Cisto monspeliensis-Sarcopoterietum spinosi*	upper thermomediterranean—upper dry	calcarenites, sands	Salento
*Thymbro capitatae-Anthyllidetum japygicae*	upper thermomediterranean—upper dry	limestones	Salento, south of Gallipoli
*S* *aturejo cuneifoliae-Ericetum manipuliflorae*	upper thermomediterranean—dry to lower subhumid	calcarenites	Eastern side of Salento
*Vicio giacominianae-Helianthemetum jonii*	upper thermomediterranean lower subhumid	calcarenites	Salento, south of Otranto
*Cisto eriocephali-Phlomidetum fruticosae*	upper thermomediterranean lower subhumid	calcarenites	Salento
*Plantago holostei-Thymbretum capitatae*	upper thermomediterranean lower subhumid	marly limestones	Salento, near Otranto
*Sileno otitis-Helianthemetum lippii*	upper thermomediterranean—lower dry	sands	Ionian arc
*Helianthemo jonii-Thymetum capitati*	mesomediterranean—upper dry	limestones	Ionian arc
*Phagnalo annotici-Fumanetum thymifoliae*	lower to upper mesomediterranean—upper dry to lower subhumid	limestones	Gargano, Ionian arc
*Ruto chalepensis-Salvietum trilobae*	lower mesomediterranean—upper dry	limestones	Ionian arc
*Chamaecytiso spinescentis-Cistetum eriocephali*	upper mesomediterranean—lower subhumid	calcarenites	Ionian arc at Laterza
*Erico multiflorae-Halimietum halimifolii*	lower mesomediterranean—upper dry	sands	N Apulia
*Cistetum salvifolio-clusii*	lower mesomediterranean—upper dry	sands	N Apulia
*Helianthemo jonii-Fumanetum thymifoliae*	lower mesomediterranean—lower dry	sands	N Apulia; Ionian arc
*Centaureo subtilis-Thymetum capitati*	upper mesomediterranean—upper dry	limestones	Murgia materana
*Fumano ericifoliae-Centaureetum subtilis*	upper mesomediterranean to lower supramediterranean—lower subhumid	limestones	Gargano
*Chamaecityso spinescentis-Genistetum michelii*	upper mesomediterranean to lower supramediterranean—lower subhumid	limestones	Gargano
*Centaureo tenacissimae-Euphorbietum spinosae*	upper mesomediterranean—lower subhumid	limestones	Gargano
*Rhamno saxatili-Saturejetum montanae*	upper mesomediterranean—lower subhumid	limestones	Alta Murgia

## Appendix E

Dates and localities (UTM WGS84) of the phytosociological relevés.

Table A1—Rels. 1–6: Gehu et al. (1984), Table 1, rels. 1–6; Rel. 7: 20 April 2016, Marina di Lizzano, near Taranto, 33T 710653E, 4465464N; Rels. 8–9: Gehu et al. (1984), Table 1, rels. 8, 15; Rels. 10–12: 20 April 2016, La Torretta, near Taranto, 33T 703732E, 4467678N; 703760E, 4467685N; 704329E, 4467403N; Rels. 13–16: 20 April 2016, Campomarino (TA), 33T 714056E, 4463623N; 714032E, 4463570N; 714437E, 4463616N; 730276E, 4464835N; Rels. 17–19: 20 April 2016, sand coast between Campomarino and Monaco Mirante (TA), 33T 718970E, 4464145N; 718860E, 4464224N; 718452E, 4464138N; Rel. 20: 20 April 2016, sand coast between Monaco Mirante and S.P. Bevagna (TA), 33T 722349E, 4464503N; Rels. 21–24: 20 April 2016, Marina di Pulsano, near Taranto, 33T 702996E, 4468078N; 702964E, 4468139N; 702962E, 4468180N; 703432E, 4467725N; Rels. 25–27: 16 July 2007 Torre Guaceto (BR), 33T 735043E, 4511134N; 734833E, 4511173N; 734727E, 4511202N; Rel. 28: 11 March 2008 Torre Guaceto (BR), 33T 735082E, 4511137N.

Table A2—Rel. 1: 5 June 2008, Canale Giancola, near Brindisi, 33T 742459E, 4507982N; Rels. 2–3: 1 July 2008, Pantanagianni, near Brindisi, 33T 730104E, 4514470N; 730135E, 4514476N; Rels. 4–5: 30 April 2015, Lido Bruno, Taranto, 33T 689928E, 4474799N; 690041E, 4474668N; Rels. 6–7: 4 June 2015, Canale Giancola, near Brindisi, 33T 742390E, 4507846N; 742459E, 4507987N; Rels. 8: 23 May 2018, Punta Palascia, near Otranto (LE), 34T 287745E 4442116N; Rels. 9–10: 23 May 2018, Residence La Torre, Otranto (LE), 34T 287667E, 4446082N; 287684E, 4446078N.

Table A3—Rels. 1–5: Caniglia et al. (1975), Table 1, rels. 3–6, 9; Rels. 6–7: 23 April 2015, Palude del Capitano (LE), 33T 748585E, 4454277N; 748670E, 4454284N; Rels. 8–12: 12 May 2017, Punta penna, near Brindisi, 33T 748435E, 4507421N; 748209E, 4507619N; 748081E, 4507718N; 748427E, 4507413N; 748350E, 4507532N; Rel. 13: 2 May 2002, Palude del Capitano (LE).

Table A4—Rels. 1–8: 14 May 2015, Punta Pizzo (LE), 34S 244318E, 4430198N; 244286E, 4430246N; 244263E, 4430358N; 244277E, 4430385N; 244210E, 4430517N; 243900E, 4430797N; 243902E, 4430862N; 243864E, 4430897N; Rels. 9–14: 23 April 2002, Punta Pizzo (LE).

Table A5—Rels. 1–10: Brullo et al. (1987), Table 1; Rel. 11: 21 November 2007, Apani, Torre Guaceto (BR), 33T 738336E, 4509133N; Rels. 12–17: 25 October 2007, Le Cesine (LE), 34T 271009E, 4471559N; 271012E, 4471515N; 271026E, 4471408N; 275525E, 4467213N; 275709E, 4467221N; 275226E, 4467110N.

Table A6—Rels. 1–5: 15 April 2016, Porto Badisco (LE), 34T 285174E, 4439813N; 285216E, 4439756N; 285215E, 4439629N; 285198E, 4439778N; 285151E, 4439847N; Rels. 6–13: 2 May 2002, Porto Badisco (LE).

Table A7—Rels. 1–3: 15 April 016, Porto Badisco (LE), 34T 285174E, 4439813N; 285216E, 4439756N; 285215E, 4439629N; Rels. 4–5: 12 March 2023, Porto Badisco (LE), 34T 285496E, 4439964N; 285521E, 4439960N.

Table A8—Rels. 1–5: 5 May 2020, Selva del Turchese, Giurdignano (LE), 34T 284472E, 4444974N; 284473E, 4444978N; 284475E, 4444985N; 284475E, 4445101N; 284482E, 4445007N; Rel. 6: 5 May 2020, Torre S. Emiliano, Uggiano la Chiesa (LE), 34T 285604E, 4441936N; Rels. 7–16: 03/05/2018, Selva del Turchese, Giurdignano (LE), 34T 284488E, 4444692N; 284499E, 4444634N; 284464E, 4444681N; 284459E, 4444707N; 284452E, 4444723N; 284431E, 4444837N; 284431E, 4444893N; 285607E, 4441825N; 285573E, 4441847N; 285525E, 4441913N.

Table A9—Rels. 1–6: Biondi & Guerra (2008), Table 17, rels. 3–8; Rels. 7–11: Di Pietro & Misano (2010) Table XIII rels 5–8,10; Rel. 12: 23 November 2007, Laterza-Castellaneta (Giacoia), 33T 658244E, 4494865N; Rel. 13: 11 May 2007, Laterza (TA), 33T 653403E, 4494861N; Rel. 14: 30 May 2007, Palagianello (TA), 33T 666337E, 4498608N; 666723E, 4498346N; Rel. 15: 30 May 2007, Laterza-Castellaneta (TA), 33T 659592E, 4496765N; Rel. 16: 7 June 2007, Montecamplo (TA), 33T 659686E, 4497702N; Rel. 17: 7 June 2007, Laterza (TA), 33T 658281E, 4495060N; Rel. 18: 18/06/2007, Laterza (TA), 33T 655762E, 4493402N; Rel. 19: 29 June 2007, Laterza (TA), 33T 655242E, 4493781N; Rel. 20: 10 July 2007, Laterza (TA), 33T 654996E, 4494994N; Rel. 21: 12 July 2007, Gravina Madonna della Scala, Massafra (TA), 33T 679858E, 4498004N; Rel. 22: 12 July 2007, Crispiano (TA), 33T 685768E, 4498391N; Rel. 23: 12/07/2007, Statte (TA), 33T 686880E, 4494572N; Rel. 24: 14 September 2007, Gravina di Ginosa (TA), 33T 649596E, 4493475N; Rel. 25: 8 November 2007, Laterza (TA), 33T 655366E, 4495938N; Rels. 26–28: 26/08/2008, Grottaglie (TA), 33T 708470E, 4487140N; 708293E, 4487094N; 708205E, 4487085N; 709397E, 4485525N; Rel. 29: 26 August 2008, Grottaglie (TA), 33T 705127E, 4493887N; Rels. 30–31: 06/05/2020, Cave di Pietra, Gravina di Fantiano, Grottole (TA), 33T 704042E, 4492707N; 704104E, 4492646N.

Table A10—Rels. 1–6: Biondi (2000), Table 3; Rels. 7–10: Biondi & Guerra (2008), Table 22 rels. 1–2, 4–5; Rels. 11–12: 11 May 2018, Gravina di Laterza (TA), 33T 653308E, 4496594N; 653278E, 4496581N; Rels. 13–14: 6 May 2020, Gravina di Mottola (TA), 33T 674367E, 4499146N; 674364E, 4499134N.

Table A11—Rels. 1–6: 12 May 2015, Chiatona (TA), 33T 675602E, 4487471N; 675510E, 4487408N; 675421E, 4487397N; 675473E, 4487392N; 678568E, 4487680N; 678575E, 4487654N; Rels. 7–9: 28 May 2015, Borgo Pineto, Castellaneta (TA), 33T 662345E, 4479059N; 662335E, 4479028N; 662223E, 4478928N; Rels. 10: 18 May 2020, Chiatona lido (TA), 33T 675923E, 4487490N; Rels. 11–12: 18 May 2020, Borgo Pineto, Castellaneta (TA), 33T 661759E, 4478415N; 661627E, 4478283N.

Table A12—Rels. 1–5: Biondi & Guerra (2008), Table 18, rels. 1–5.

Table A13—Rels. 1–6: Biondi & Guerra (2008), Table 19, rels. 1–6.

Table A14—Rels. 1–6: Taffetani & Biondi (1989), Table 6, rels. 2–4, 7–9; Rels. 7–12: 6 May 2015, Bosco Isola di Lesina, Lesina (FG), 33T 529303E, 4639226N; 529284E, 4639155N; 529248E, 4639194N; 532378E, 4638283N; 532440E, 4638370N; 532707E, 4638151N; Rel. 13: 17 April 2018, Bosco Isola di Lesina, Lesina (FG), 33T 529757E, 4638416N; Rels. 14–18: 6 July 2002, Bosco Isola di Lesina, Lesina (FG).

Table A15—Rels. 1–7: Taffetani & Biondi (1989), Table 5, rels. 1–7; Rel. 8: 6 May 2015, Bosco Isola di Lesina, Lesina (FG), 33T 531223E, 4632151N; Rels. 9–14: 17 April 2018, Bosco Isola di Lesina, Lesina (FG), 33T 529781E, 4638419N; 529783E, 4638419N; 530058E, 4638388N; 530120E, 4638373N; 530162E, 4638377N; 532347E, 4638545N; Rels. 15–18: 28 May 2015, Borgo Pineto, Castellaneta (TA), 33T 662566E, 4479704N; 662484E, 4479786N; 662479E, 4479836N; 662499E, 4479974 N; Rel. 19: 28 May 2015, Riserva Stornara, Palagiano (TA), 33T 668483E, 4485188N.

Table A16—Rels. 1–7: Caniglia et al. (1976), Table 1.

Table A17—Rels. 1–12: Terzi M., D’Amico S. (2006), Table 3, res. 5–16; Rels. 13–19: 7 July 2002, Murge materane, Matera.

Table A18—Rels. 1–6: 196 2015, Monte S. Angelo (FG), 33T 576847E, 4616387N; 576793E, 4616074N; 576770E, 4615902N; 576838E, 4616533N; 576844E, 4616569N; 576784E, 4614514N; Rels. 7–13: 26 May 2016, Monte S. Angelo (FG), 33T 580539E, 4615157N; 580890E, 4615209N; 578893E, 4613802N; 578849E, 4614045N; 582526E, 4617157N; 580801E, 4615224N; 575601E, 4616131N; Rels. 14–17: 29 May 2018, Vallone di Petrulo (FG), 33T 577097E, 4616368N; 577097E, 4616368N; 577144E, 4616351N; 576991E, 4616575N.

Table A19—Rels. 1–10: De Faveri & Nimis (1982), Table 1, rels. 21–24, 26–30, 32; Rels. 11–12: 26 May 2016, Monte S. Angelo (FG), 33T 575568E, 4616164N; 575513E, 4615701N; Rels. 13–16: 7 June 1982, Monte S.Angelo (FG).

Table A20—Rels. 1–4: 20 May 1992, Gargano (FG), southern slope.

Table A21—Rels. 1–20: Tomaselli et al. (2021), Table 1, rels. 9–28; Rels. 21–24: Biondi & Guerra (2008), Table 20, rels. 1–2, 4–5; Rels. 25–27: 27 April 2016, Gravina di Laterza (TA), 33T 653300E, 4496988N; 653300E, 4496926N; 654479E, 4499780N.
